# *Operando* spectroelectrochemical identification of peroxide intermediate in molten carbonate CO_2_-to-carbon electroreduction

**DOI:** 10.1038/s41467-026-70977-0

**Published:** 2026-04-21

**Authors:** Sander Ratso, Michael L. Whittaker, Kätlin Kaare, Raluca O. Scarlat

**Affiliations:** 1 Department of Nuclear Engineering, University of California, Berkeley, CA USA; 2 National Institute of Chemical Physics and Biophysics, Akadeemia tee 23, Tallinn, Estonia; 3 Energy Geosciences Division, Lawrence Berkeley National Laboratory, Berkeley, CA USA; 4 Department of Earth & Planetary Sciences, UC Berkeley, Berkeley, CA USA

**Keywords:** Electrochemistry, Surface spectroscopy, Raman spectroscopy, Sustainability, Reaction kinetics and dynamics

## Abstract

Electrolysis of CO_2_ in molten salts promises efficient carbon capture, but the underlying reaction mechanisms remain incompletely understood, as research thus far has been limited by a lack of tools for *operando* investigations. Here, we use a high-temperature *operando* Raman spectroelectrochemical system to look for signatures of reaction intermediates and study the evolution of carbon structures over electrolysis time. The analysis reveals the existence of O_2_^2−^ concurrently with the deposition of carbon on Au, W, Inconel, and Ni electrode materials, pointing to a common reaction mechanism with O_2_^2−^ as an intermediate. Secondly, the G peak of the as-deposited carbon experiences a noticeable blue-shift as the material is cooled down and purified, suggesting either a growth in crystallite size even after the electrolysis is stopped or lithium deintercalation. Elucidating the cathodic carbon deposition mechanism could help create greater value-added products and increase the economic viability of carbon capture.

## Abstract (web summary)

Electrochemical conversion of carbon dioxide in molten carbonates is proposed to proceed through multiple reaction pathways. Here, the authors report *operando* Raman spectroelectrochemistry reveals spectral features consistent with a peroxide intermediate forming concurrently with carbon deposition.

## Introduction

Direct air capture with carbon dioxide reduction consumes an industrial waste product, CO_2_, from the atmosphere and has the desirable products of carbon and organic molecules, and the potentially valuable O_2_ gaseous by-product. For the production of carbon products, a lifecycle CO2e (CO_2_ equivalent) of −0.890 to −0.971 can be achieved (depending on the renewable or nuclear source of energy used)^1^, as compared to a positive CO2e for the mining of carbon products^2,3^.

The carbon reduction reaction by itself ${{\rm{C}}}{{{\rm{O}}}}_{2}\left({{\rm{g}}}\right)={{\rm{C}}}+{{{\rm{O}}}}_{2}\left({{\rm{g}}}\right)$ is not spontaneous $(\Delta {G}_{{rx},\,{25}^{\circ }C}^{\circ }=\,+394.3\frac{kJ}{mol})$, and requires heat input $(\triangle {H}_{{rx},{25}^{\circ }C}^{\circ }=\,+393.5\frac{{kJ}}{{mol}})$. Thus, chemically driving the reduction of CO_2_ requires severe reducing agents (e.g., hydrogen, amines, or hydroxides) and catalysts (e.g., Pt, Ni, and Ru^4,5^), which have their corresponding energy and cost inputs for their production^6,7^. Electrochemically driving the reduction reaction of CO_2_ (CO_2_RR) instead uses electrons as the reducing agent. Across its lifecycle, combustion of hydrocarbons followed by direct air capture and utilization of CO_2_ is exothermic and thermodynamically spontaneous: ${{\rm{C}}}{{{\rm{H}}}}_{4}\left({{\rm{g}}}\right)+{{{\rm{O}}}}_{2}\left({{\rm{g}}}\right)={{\rm{C}}}+{2{{\rm{H}}}}_{2}{{\rm{O}}}$, $\triangle {G}_{{rx},\,{25}^{\circ }C}^{\circ }=-423.7\frac{{kJ}}{{mol}}; \triangle {H}_{{rx},{25}^{\circ }C}^{\circ }= -497.0\frac{{kJ}}{{mol}}.$Industrially, this is executed as hydrocarbon combustion to CO_2_ for energy production, followed by the energy-consuming conversion of CO_2_ to solid carbon products. For combined cycle gas power plants with efficiencies upwards of 60% for conversion of heat to electricity^8^, the overall process is energy positive ($> 140\frac{{kJ}}{{mol\; C}{H}_{4}}$), with the added benefit of solid carbon products and a potentially extractable oxygen gas side-product.

A convenient electrochemical medium to work with is water. Aqueous CO_2_ electroreduction technologies struggle to reach selectivity >90% and current densities >0.15 A cm^−2^^6,9^ because aqueous solutions are restricted by low operating temperature (<100 ^o^C and unpressurized) and hence slow reaction kinetics, and low CO_2_ solubility (0.033 mol CO_2_ L^−1^ water at 25 ^o^C^10^), which also limits the rate of reaction (see current densities above). Furthermore, the competing hydrogen evolution reaction (standard reduction potential, ${{{\rm{E}}}}_{{25}^{\circ }C}^{\circ }\,=\,-1.23{{\rm{V}}}$ vs. oxygen evolution reaction, OER) limits the overpotential that can be applied to overcome the kinetic limit of the CO_2_ reduction reaction (${{{\rm{E}}}}_{{25}^{\circ }C}^{\circ }\,=\,-1.02{{\rm{V}}}$ vs OER)^7^ to less than 160 mV (and even lower, at temperatures higher than 25 ^o^C), and also limits the selectivity of the carbon reduction reaction (40 to 80% carbon conversion efficiency^11^).

Molten salts are a compelling alternative electrochemical medium. CO_2_ electrolysis in molten salt (CO2MSE) enables operating temperatures of 400–750 °C and has a wide electrochemical window (>3 V). Fast reaction kinetics that are enabled by high reaction temperatures in the ionic melts alongside high ionic conductivity have allowed for Faraday efficiencies of >95% and current densities >2 A cm^−2^^12^, with ~100% selectivity to be reached at <800 °C.

The current understanding of the reduction mechanism of CO_2_ to carbon in molten carbonates includes the rapid, exothermic reaction of gas phase CO_2_ with oxide in the electrolyte to form carbonate, with a simultaneous reduction of cathodic carbonate to form carbon and anodic oxidation of oxide to form oxygen. By CO2MSE, the synthesis of varied nanocarbon products, including various types of carbon nanotubes^13^, nano-platelets and graphene^14^, doped carbons^15,16^ and even oxide-carbon composites^17^ has been demonstrated. Transition metal nucleation at the cathode can drive the formation of specific graphene nanocarbon morphologies, with both root and tip growth mechanisms having been proposed. High concentrations of iron or nickel can drive carbide formation, leading to magnetic CNTs^18^. Conversely, inhibiting transition metal nucleation suppresses 1D CNT growth and facilitates the formation of 0D carbon nano-onions^19^, 2D graphene and carbon nano-platelets^14^, and 3D carbon nano-scaffolds^20^. Correlations between output products, deposition current, alkalinity of the electrolyte, temperature, and electrode materials have been made^21–25^, while progress in looking at the mechanistic details of this process remains limited. Previous operando studies have employed electrochemistry in tandem with gas analysis in real time to monitor CO_2_ reduction in molten carbonates^26^. While these electrochemical and gas-evolution measurements provide important operando insights, the mechanistic details of intermediate species have remained limited due to the lack of spectroscopic operando investigation.

Four main reaction pathways for the cathode reaction in a molten carbonate electrolyzer have been proposed^21^:

An electrochemical-chemical pathway, where the first step is the deposition of alkali metal:

1 $${{{\rm{M}}}}^{+}+{{{\rm{e}}}}^{-}={{\rm{M}}}$$

where (M=Li,Na,K) and the second step is a chemical reaction of the alkali metal with the carbonate anion^27^:

2 $$4{{\rm{M}}}+{{\rm{C}}}{{{{\rm{O}}}}_{3}}^{2-}=2{{{\rm{M}}}}_{2}{{\rm{O}}}+{{\rm{C}}}+\,{{{\rm{O}}}}^{2-}$$

In melts containing only alkali carbonate and oxide anions, this pathway for cathodic deposition could only possibly occur in lithium-free melts, where the deposition potentials of alkali metals are close to that of carbon deposition (see Supplementary Tables 1 and 2). However, no carbon deposits have been witnessed in pure Na-K carbonates with no lithium^21^.

A 2 + 2e^–^ pathway, where in the first step, hypothetical CO_2_^2–^ anions are formed:

3 $${{\rm{C}}}{{{{\rm{O}}}}_{3}}^{2-}+2\,{{{\rm{e}}}}^{-}={{\rm{C}}}{{{{\rm{O}}}}_{2}}^{2-}+\,{{{\rm{O}}}}^{2-}$$

after which these anions are in turn reduced to solid carbon and oxide:

4 $${{\rm{C}}}{{{{\rm{O}}}}_{2}}^{2-}+2\,{{{\rm{e}}}}^{-}={{\rm{C}}}+\,{2{{\rm{O}}}}^{2-}$$

The evidence for this mechanism comes from an electrochemical study in molten Li-Na-K carbonate on a gold flag electrode^28^. However, the CO_2_^2–^ is not a stable anion, and it should at least partially decompose to CO and O_2_^2–^, which have not been seen in any study to date.

A pathway involving a peroxide intermediate:

5 $$2{{\rm{C}}}{{{{\rm{O}}}}_{3}}^{{2}{-}}+2\,{{{\rm{e}}}}^{-}=2{{\rm{C}}}+{3{{{\rm{O}}}}_{2}}^{{2}{-}}$$

6 $${{{{\rm{O}}}}_{2}}^{{2}{-}}+2\,{{{\rm{e}}}}^{-}={2{{\rm{O}}}}^{{2}{-}}$$

Notably, the standard potential for reaction (7) is very negative at $-$5.98 V, however, peroxide anions have been detected in separate electrochemical and spectroscopic studies, leading to a hypothesis that they take part in the reaction as adsorbed intermediates^29,30^. Itoh et al. also noted an appearance of peroxide-related Raman peaks in Li- and (Li-K)_2_CO_3_ melts^31,32^.

A direct four-electron process in which the carbonate anions are then split into solid carbon and oxide anions on the cathode:

7 $${{\rm{C}}}{{{{\rm{O}}}}_{3}}^{{2}{-}}+4\,{{{\rm{e}}}}^{-}={{\rm{C}}}+\,{3{{\rm{O}}}}^{{2}{-}}\,$$

Due to the lack of direct evidence of intermediates of other pathways and the observation of no solid carbon being formed even in pure Na-K carbonate, combined with electrochemical evidence for a four-electron reduction process^33^, most authors have agreed that reaction (9) is the most probable reaction for cathodic carbon deposition in molten carbonates. Additionally, it has been suggested that CO_2_ can also be chemically dissolved in the carbonate to form dicarbonate anions (C_2_O_5_^2–^), which could then also take part in the reaction mechanism as an intermediate for CO_2_ capture^34^:

8 $${{{\rm{CO}}}}_{2}+{{\rm{C}}}{{{{\rm{O}}}}_{3}}^{{2}{-}}\leftrightharpoons \,{{{\rm{C}}}}_{2}{{{{\rm{O}}}}_{5}}^{{2}{-}}$$

In conditions of high oxide activity (and thus low *p*CO_2_), however, the primary mechanism for CO_2_ uptake is the direct, exothermic reaction with oxide ions. This simpler pathway is thermodynamically favored and serves as the fundamental regeneration step for the carbonate electrolyte in low *p*CO_2_ environments such as air^35^. In addition to studies of the fundamental reaction mechanism, some recent advances have been made in looking at the mechanisms which govern the deposition of different carbon structures. Wang et al. proposed three mechanisms of carbon deposition on different electrodes based on the Gibbs free energies of formation of the respective carbides^24^:

9 $$x{{\rm{M}}}+\,y{{\rm{C}}}={{{\rm{M}}}}_{x}{{{\rm{C}}}}_{y}$$

and the subsequent decomposition of formed carbide:

10 $${{{\rm{M}}}}_{x}{{{\rm{C}}}}_{y}={{\rm{C}}}+{{{\rm{M}}}}_{x}{{{\rm{C}}}}_{y-1}$$

According to this theory, a moderate Δ*G*_M–C_ is necessary for the formation of *sp*^2^ carbons. This theory does not explain the full reaction mechanism for carbon deposition, and was posited on ex situ measurements, but does offer an interesting explanation to what happens with the carbon that is being deposited and explains why different electrode materials exhibit different structures for the purified deposits.

Raman spectroscopy stands out as a relevant tool for *operando* studies, specifically for molten carbonate studies due to its sensitivity to vibrational modes of carbon and oxygen bonds, which makes it able to detect carbonates, oxides and related species such as C_2_O_5_^2–^, CO_4_^2–^, peroxides and superoxides^36^. In situ studies of molten carbonates have thus far concentrated on the speciation of the melt at temperature.

Raman spectroscopy is also among the most potent tools for the study of solid carbons and their structure^37^; each carbon material has their own Raman fingerprint, which makes it possible to analyze their structure and changes in it during electrolytic deposition from molten carbonate.

Here, we demonstrate an *operando* Raman spectroelectrochemical system to detect reaction intermediates and the evolution of the structure of the deposited carbon for elucidating the reaction mechanism of direct air capture of CO_2_ in a molten carbonate melt.

## Results

### Redox behavior of Inconel 600, Ni, Au, and W electrodes

To better mimic direct air-capture conditions, unpressurized air was used as the working atmosphere. Although the deposition process itself can approach coulombic efficiencies up to 100%^38^, during product removal at temperatures above 500 °C in oxygen-rich environments (such as air), amorphous carbon can start to be oxidized in a mixture of carbonates and solid carbon^39^. The working temperature of the salt must be somewhat above the melting point of the salt to induce faster mass transfer, which is why the working temperature was set to be as high as possible without potentially oxidizing the deposited carbon. For each working electrode material, a cyclic voltammogram was recorded in tandem with microscopic observation of carbon deposition and oxygen evolution. This process on W electrode is shown in Supplementary Movie 1, where the potential is first swept at 100 mV s^−1^ towards positive potentials, with visible gas evolution (00:04 to 00:16), and then back to negative potentials, where the deposition of carbon appears (00:32 to 00:42). The calculated theoretical potentials of carbon deposition and oxygen evolution in the cell are given in Supplementary Fig. 1.

**Fig. 1:**
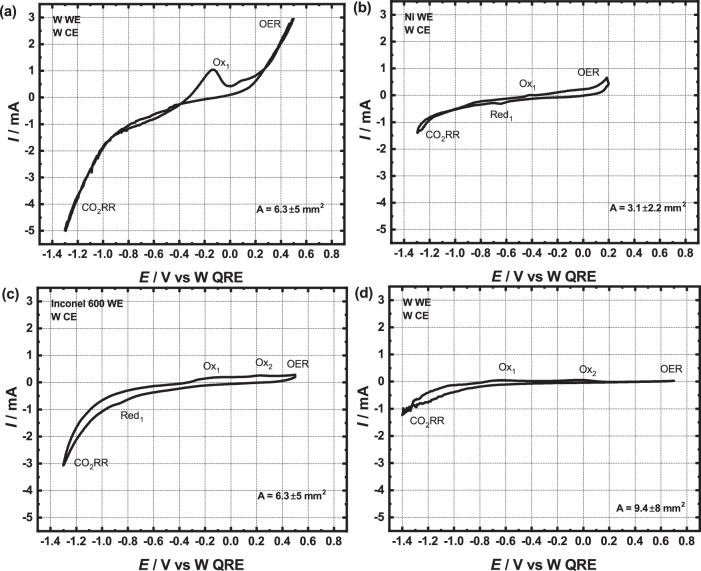
Cyclic voltammetry of different electrode materials in the *operando* cell. Cyclic voltammograms recorded in a molten eutectic mixture of Li, Na, and K carbonate on working electrodes of different materials: **a** W, **b** Ni, **c** Inconel 600, **d** Au. Temperature: 500 °C, sweep rate: 100 mV s^−1^, electrode surface areas were approximated from their circumference and likely depth of immersion in the molten salt. The CVs are non-iR-corrected. Source data are provided as a Source Data file.

In each case, after determining the potential range, three cyclic voltammograms (CVs) were recorded, and the third cycle is given in Fig. 1. The CV at W working electrode appeared as relatively featureless except for the carbon deposition (starting at −1 V vs W QRE) and oxygen evolution (starting at 0.2 V vs W QRE, Fig. 1a). There is an additional oxidation peak, Ox_1_, centered at −134 mV, which is likely related to the formation of a WO_3_ layer on the W electrode. On both Ni and Inconel 600 (Fig. 1b, c), a pair of oxidation/reduction peaks appears in addition to this, which have previously been attributed to the formation and dissolution of oxide scales^40^. In the case of Inconel 600, the oxidation peak can be seen to be made up of multiple sub-peaks due to the existence of both Fe, Cr and Ni in this alloy, which form a mixed oxide layer^40,41^. Cyclic voltammetry on the gold electrode (Fig. 1d) also revealed some peaks in addition to CO_2_RR and OER. Previous work on gold electrodes has studied the evolution of CO from CO_2_, which was visible in this system as the bubbling and the noise on the CV^42^. Here, we observe both gas evolution, which we attribute to the reduction of CO_2_ to CO, and carbon deposition on the working electrode. Because of this, the reduction peak is shifted to more negative potentials when compared to the W RE. We also observe two oxidation peaks, Ox_1_ and Ox_2_; previous electrochemical studies on gold electrodes with 100% CO_2_ cover gas, also observed the Ox_1_ and Ox_2_ peaks and attributed them to the oxidation of CO (dissolved and electrode-adsorbed) to CO_2_^42–44^.

### Evolution of carbon and intermediate species during CO_2_ electrolysis derived from *operando* Raman

**Fig. 2:**
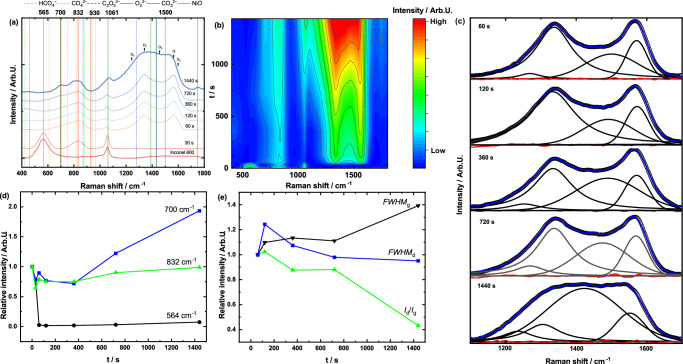
Time-dependent evolution of the carbon structure. **a**, **b** Stacked Raman spectra of the Inconel 600 and (Li,Na,K)_2_CO_3_ interface during electrolysis, **c** deconvoluted carbon regions of the Raman spectra, **d** relative intensities of Raman peaks at specific shifts and **e** *I*_D_/*I*_G_, FWHM_D_ and FWHM_G_ derived from the deconvoluted carbon spectra. The experimental spectrum is given as the dotted line, while the fit is the blue line, and the black lines represent the fitting components. The residuals are given as red lines. Source data are provided as a Source Data file.

After recording CVs and determining the useful working range of each electrode, to study the changes in the structure of the electrode-electrolyte-gas triple boundary layer and the structure of the deposited carbon phase, a potentiostatic electrolysis at −1.3 V was undertaken with each electrode, recording Raman spectra during the process (0.63 mA for 1440 s). Measuring the pristine Inconel surface immersed in molten carbonate and prior to electrolysis (Fig. 2a) revealed two main features: a peak at 1061 cm^−1^, which corresponds to the symmetrical stretching of O-C-O bonds in CO_3_^2−^ ions^36,45^, and a peak at 564 cm^−1^, which can be related either to lithium oxide species^45^ or lithiated NiO^46^. The referred literature for each discussed species is given in Supplementary Table 3. After 30 s of electrolysis, there is no signal yet arising from the carbon region at 1100–1700 cm^−1^, but notably, there is a change in the region near 700–850 cm^−1^ (centered at 832 cm^−1^), where a wide band appears. To better visualize the changes in peak intensities during electrolysis, the Raman spectra are shown as a contour plot in Fig. 2b and the changes in intensity at specific shifts in Fig. 2d. As the band centered at 832 cm^−1^ is intensified during the carbon deposition, we can relate this band to an intermediate product; adsorbed C-O_2_^2−^ is the most likely candidate with peaks in that region (peak positions for other possible carbonate species that could take part in the reaction or exist in carbonates in an oxygen-rich environment are also shown in Fig. 2a^36,46^). As electrolysis proceeds, the carbonate peak at 1061 cm^−1^ is again intensified along with the appearance of a peak at 700 cm^−1^ (from peroxide species or in-plane bending of C-O^36^), both of which are likely due to the co-deposition of carbonate into the solid carbon product. As the changes in the feature at 832 and 700 cm^−1^ are uniform prior to the final measurement at 1440 s, the origin of these features seems to be the same, but another process (such as the co-deposition of carbonate into the carbon product) further increases the intensity of the peak at 700 cm^−1^ at the final measurement point. A signal from the carbon deposit appears in the region between 1100 and 1700 cm^−1^, with the deconvoluted spectra (fitting details given in Supplementary Table 4) shown in Fig. 2c and the characteristics (*I*_D_/*I*_G_, FWHM_D_ and FWHM_G_) in Fig. 2e. During electrolysis, the *I*_D_/*I*_G_ ratio increases at first, but is then decreased somewhat, while FWHM_D_ also increases between 30 to 60 s, but is then decreased; FWHM_G_ shows a near uniform increase during electrolysis. It is important to note that the analysis of the carbon region of the Raman spectrum at 1440 s is impeded by the appearance of weak peaks of O-C-O or C_2_O_5_^2–^ species^36,47^ in the same region as carbonates are co-deposited with the carbon, which adds to the intensity of the D3 peak of carbon and make determination of *I*_D_/*I*_G_ ratios difficult. Inconel, containing Cr and Fe, would be expected to form both Fe_3_C and Cr_3_C_2_ according to their negative Δ*G*_M–C_ and the theory postulated by Wang et al. ^24^ and deposit a mixture of amorphous and *sp*^2^ carbon (since Ni should be a good substrate for the deposition of *sp*^2^ carbon). No Raman peaks specific to Fe-C, Cr-C, and Ni-C have been noted in the literature previously, so no signal would be expected even if they were present during electrolysis.^48–50^ However, the results corresponding to an identical analysis of Ni, Au and W electrodes are given in Supplementary Figs. 2–4, with overall similar observations. This means that the peroxide intermediate is present on very different electrode surfaces. Importantly, no signal from W-C bonds (at Raman shifts of 693 and 807 cm^−1^ ^51^) was seen at any time during the deposition process on a W electrode (Supplementary Fig. 4), making a carbide-mediated deposition reaction mechanism unlikely, at least for direct air capture (low partial pressure of CO_2_).

### Potential-dependent Raman spectra of the W electrode

**Fig. 3:**
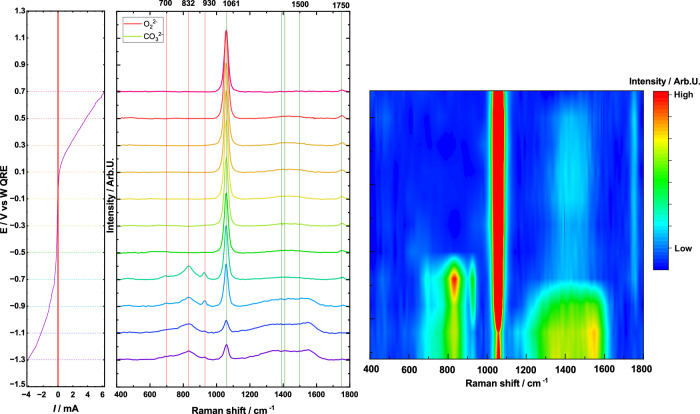
Potential-dependent *operando* Raman measurements. Potential-dependent Raman spectra of the interface between a W electrode and the eutectic (Li,Na,K)_2_CO_3_ salt mixture during a linear sweep from 0.7 to −1.3 V vs W QRE. Source data are provided as a Source Data file. The LSV is presented as-measured and is not iR-corrected.

To further confirm that no tungsten carbides are formed and that the presence of peroxide-related features around 830 cm^−1^ precedes the deposition of carbon, linear sweep voltammetry (LSV) was performed at a sweep rate of 10 mV s^−1^, with Raman spectra measured at every 200 mV from 0.7 V vs W QRE to −1.3 V vs W QRE (Fig. 3). The LSV from 0.7 to −0.5 V vs QRE is largely dominated by the oxygen evolution current, with signal visible only from the carbonate peak near 1061 cm^−1^. At −0.7 V, reduction current appears on the LSV and the features around 830 cm^−1^ consistent with O_2_^2−^ become visible simultaneously with the onset of reduction current, but no C signal from 1100 to 1800 cm^−1^ is seen yet, likely due to the low magnitude of reduction current at this potential (0.46 mA). As the potential becomes even more negative, the signal from carbon increases as it is gradually deposited. No signal from W-C bonds is visible at any point on the Raman spectra, and no other intermediate-related features are seen either.

### Effect of purification and temperature on the Raman spectra of CO_2_-derived carbon

**Fig. 4:**
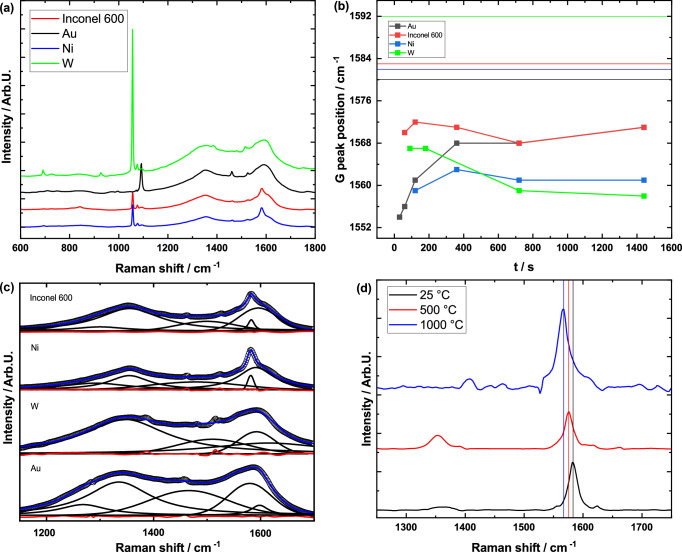
Effects of cooling and purification to the carbon deposit. **a** Raman spectra of the deposits after cooling to 25 °C, **b** G peak position over electrolysis time, with the peak position at 25 °C shown as reference lines with corresponding colors, **c** deconvoluted carbon regions of the 25 °C Raman spectra and **d** G peak position shift of a graphite crucible. Source data are provided as a Source Data file.

Raman spectra of purified deposits (Fig. 4a and deconvolution on Fig. 4c), revealed a much higher content of *sp*^2^ carbon when compared to the high-temperature Raman spectra, which is apparent in the shifted and more intense G peak. The G peak is also significantly blue-shifted. To elucidate the effect of temperature and other possible factors, the G peak position (*ω*_G_) was plotted against electrolysis time (Fig. 4b), with the 25 °C positions given as reference lines of corresponding colors. As can be seen, for each of the materials there is a significant shift when the material is cooled down and washed, with the Au electrode also exhibiting a blue-shift of the peak with increasing electrolysis time. Some of this shift can be explained by temperature, as the G peak experiences a shift of 0.016 cm^−1^ K^−1^ due to a lengthening of the C-C bonds with temperature^52^. A temperature change from 773 to 298 K would be expected to lead to a blue-shift of 7.6 cm^−1^, which is insufficient to explain the shifts demonstrated here. This effect was confirmed using the exact setup used for *operando* studies, except the laser was focused onto the surface of a graphite crucible at different temperatures (Fig. 4d). Going from 1000 to 500 °C shifted the G peak for the graphite crucible from 1567 to 1575.5 cm^−1^ and then to 1583 cm^−1^ at 25 °C, following the shift as reported by ref. ^52^. This temperature-induced shift has been proposed for standardized materials such as graphene and CNTs, however. In the case of pyrocarbons, which would be more comparable to the carbon deposited from CO_2_, Mallet-Ladeira et al. reported no change in spectra going from 300 to 3.8 K^53^. To confirm the magnitude of temperature effects directly on the as-deposited carbon, the purified material was reheated to 500 °C and the Raman spectra remeasured (Supplementary Fig. 5). An increase in crystallite size and decrease in disorder can also lead to a blue-shift of the G peak^54^ up to a maximum of 4 nm, after which larger crystallites will again red-shift the G peak^55^ and this effect is likely to be present here when considering the other changes in the spectra. Finally, a possible reason for part of the red-shift can be lithiation of the *sp*^2^ crystallites, as lithiation has been shown to have this effect in previous *operando* battery studies^56^. This is another effect likely at play here due to the presence of a high concentration of lithium and the co-deposition of lithium carbonate.

## Discussion

The observations made of the intermediate species, possible due to the *operando* spectroelectrochemical method demonstrated here, provided mechanistic details about the CO2MSE:

The deposition mechanism of carbon in direct air capture conditions includes a peroxide intermediate on all electrodes (W, Ni, Inconel 600, Au), exhibited as an increase in the Raman band centered at 830 cm^−1^; the intensity of the band increases with electrolysis time, and varies with applied reduction potential. The likely reaction mechanism is therefore a two-step pathway:

11 $$2{{\rm{C}}}{{{{\rm{O}}}}_{3}}^{2-}\left({{\rm{salt}}}\right)+2\,{{{\rm{e}}}}^{-}\leftrightharpoons 2{{\rm{C}}}({{\rm{s}}})+{3{{{\rm{O}}}}_{2}}^{2-}({{\rm{ads}}})$$

12 $${{{{\rm{O}}}}_{2}}^{2-}({{\rm{ads}}})+2\,{{{\rm{e}}}}^{-}={2{{\rm{O}}}}^{2-}({{\rm{salt}}})$$

**Fig. 5:**
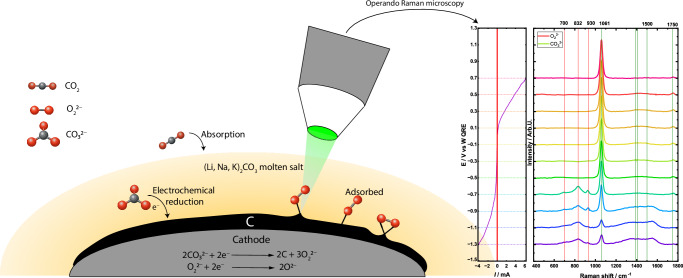
Mechanism for CO2MSE in molten carbonate.

This mechanism was first proposed by ref. ^29^, who postulated it on the basis of refs. ^57,58^ work from the 1970s on ionic complexes, but considered it unlikely due to the very negative standard potential for reaction (13) of $-$5.98 V. They did theorize, however, that strong interactions between the deposited carbon and peroxide could stabilize it enough for this pathway to be viable due to their finding of lithium peroxide in the carbon deposits. As peroxide-related bands in the Raman spectra were intensified concurrently with carbon deposition (Fig. 5), this indeed seems to be the case. Among the oxygen species possible in molten carbonates, peroxide (O_2_^2−^) is the only intermediate evidenced by operando Raman spectroscopy. Bands characteristic of superoxide (1047–1160 cm⁻^1^) were absent under all conditions, ruling out its involvement. While the concentration of oxide ions (O^2−^), which have no Raman signal, is expected to increase during carbon deposition as they are the second final product, this does not explain the sudden increase in peroxide content. In contrast, the peroxide band at ~830 cm^−1^ emerges reproducibly and increases concurrently with carbon deposition, providing direct evidence that O_2_^2−^ is the mechanistic intermediate driving the CO_2_-to-carbon pathway under our conditions.

Whereas a carbon-adsorbed peroxide intermediate does explain the reaction mechanism at high oxygen concentrations (direct air capture conditions), it does not explain the differences in the structure of carbon seen on different electrodes. The reaction seems to be proceeding via the same mechanism as peroxide-related features are seen on Raman spectra for all of the electrodes and intensify as the reaction proceeds, but the resulting carbons are very different, both in their surface morphology and structure (showing a mixture of amorphous/graphitic domains on Supplementary Figs. 6–10), Raman spectra at high temperatures and even more interestingly, in how the signal changes as the material is cooled down and purified. The apparent differences in the carbon region Raman spectra between the high-temperature as-deposited materials and the purified 25 °C spectra point to lithium intercalation playing an important role in the deposition process. Notable effects are also present from temperature and change in disorder going from high-temperature to low-temperature, which is witnessed as the shift in the G peak, but the exact contribution from these effects is still unknown and requires further study.

We have performed spectroelectrochemical *operando* observations of CO_2_ reduction in molten carbonate, and shown that the most likely mechanism is two reaction steps, both two-electrons, with the formation of a carbon-adsorbed peroxide intermediate. Among the four previously hypothesized mechanisms for this reaction, the peroxide route was not expected to be accessible; we demonstrate here the contrary. We emphasize that further proof, including kinetic studies determining the electron transfer number during the rate-limiting step and chemical quenching experiments of peroxide are necessary to definitively confirm this mechanism. These findings provide a basis for investigating the role of dissolved additives and structure of the molten salt solvent in accessing specific carbon structures, such as nanotubes, and for understanding the role of gas composition on the structure of the resulting product. This provides additional understanding of carbon reduction process from direct air capture and industrial waste streams, and enables design of carbon materials from CO_2_ with control of structure and real-time process monitoring, but systematic studies at other CO_2_ partial pressures, current densities, temperatures, electrodes and electrolyte compositions are essential to widen these prospects.

## Methods

### Materials and samples

Lithium carbonate (Li_2_CO_3_, 99.999%, Aldrich), Na_2_CO_3_ (≥99.5%, Aldrich), and K_2_CO_3_ (≥99%, Aldrich) were used as-received and not purified further. The salts were mixed together in a glovebox (<3 ppm O_2_, <1 ppm H_2_O) and ground up in an agate mortar and pestle before being stored under argon. About 2-mm diameter Gold (99.999%, Goodfellow), 1 mm Inconel 600, and 0.5 mm Ni wire, and a 1 mm diameter W probe were used as working electrodes. The quasi-reference electrode and counter electrode were W probes in all tests, except for two tests done to eliminate potential shift effects from the W QRE (Supplementary Information section 7). High purity Al_2_O_3_ crucibles (volume of 0.226 cm^3^, AdValue Technology) were used as molten salt containers except for the test looking at the temperature dependence of the G peak for graphite.

### Spectroelectrochemical cell details

**Fig. 6:**
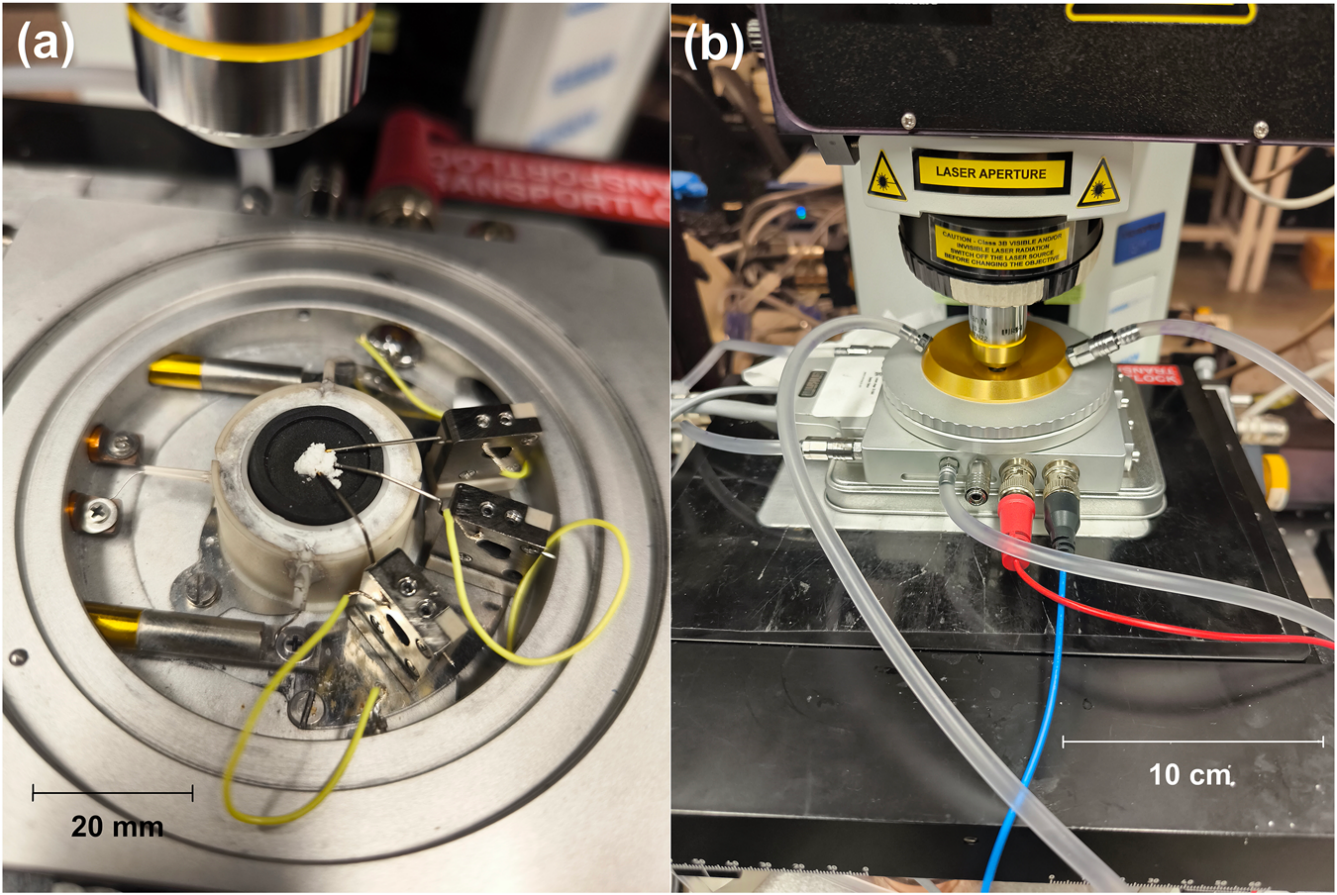
Spectroelectrochemical setup used in the study. **a** View of the internals of the Linkam Stage. **b** View of the setup connected to the confocal Raman microscope.

*Operando* Raman spectroelectrochemical experiments were conducted using a Linkam TS1000E-PB4 sample stage (herein called stage) with sapphire windows and three probe connectors connecting to a Gamry 1010e potentiostat for the working, counter, and reference electrodes, respectively. A view of the insides of the reactor showing the electrode connections is shown in Fig. 6. The outer shell and top window of the stage was constantly water-cooled by a water circulation pump. The salt samples were inserted into Al_2_O_3_ crucibles on top of sapphire sample plates to protect the stage and dried for at least 1 h at 300 °C to remove moisture from the salt. The cell temperature was calibrated using the melting point of the salt, which was determined by microscopic observation.

### Spectroelectrochemical measurements

For investigating the details of the deposition mechanism of carbon from CO_2_ in molten carbonate, the electrode assembly was heated at 300 °C for at least 1 h to dry the salt and then taken to the working temperature, which was selected as 500 °C. The heating rate was 50 °C min^−1^ and the cell was open to the surrounding atmosphere during drying with no forced ventilation. After drying the salt and calibration of the cell temperature, the cell was centered under a Horiba Jobin-Yvon LabRAM HR Evolution Confocal Raman microscope, which was calibrated using a Si wafer prior to measurements. The overall setup is shown in Fig. 6.

The 532 nm excitation laser (80 mW) was focused through the top sapphire window onto the triple boundary layer between the molten salt, gas environment and electrode surface. The Raman spectra were collected using a 600 grating, 1000 µm hole size and 200 µm slit size using a 10x microscope lens from 100 to 1800 cm^–1^. After melting the salt and waiting at least 30 min for the salt to equilibrate with the surrounding atmosphere, the laser was refocused to the triple boundary layer and a background Raman measurement of the electrode surface prior to deposition was done. Potentiostatic deposition of carbon at −1.3 V vs W QRE (−1.4 V on the gold electrode) was then undertaken for periods of 30, 60, 120, 360, 720, and 1440 s prior to which it was confirmed that carbon deposition begins before that potential on each of the electrodes by running a cyclic voltammetry (CV) and visually confirming OER and CO_2_RR (for W, these periods were 90, 180, 720, and 1440 s, respectively). To confirm concurrent Raman signal from carbon and peroxide and eliminate potential shift effects from the W QRE, LSVs were measured in the *operando* cell at a sweep rate of 10 mV s^−1^ using both W, Ni and Ag QREs (Supplementary Figs. 11 and 12). W oxidation was eliminated as an alternative anodic limit via chronoamperometry and determination of W in the melt (Supplementary Figs. 13 and 14). Due to the difficulties in measuring and controlling the real surface area of electrodes, potentiostatic deposition was preferred over a constant current mode. No surface enhancement effects were used for collecting Raman signals. Raman spectra were collected simultaneously with the deposition. Finally, the sample was cooled down, and a 25 °C spectrum was collected from the same spot. The samples were exposed to room air via open electrode ports to mimic direct air capture. All data presented is as-measured and non-iR-corrected.

To study the effect of temperature and purification on the as-deposited CO_2_-derived carbon, the materials were removed from the electrode and purified in a 1 M HCl solution, washed with water, and dried. These materials were then used for further spectroscopic experiments.

## Supplementary information

Supplementary Information

Description of Additional Supplementary Files

Supplementary Movie 1

Transparent Peer Review file

## Source data

Source Data

## Data Availability

Additional data on the determination of the electrochemical window, Raman signatures and their determination, deconvolution of the carbon region of the Raman spectra, additional Raman spectroelectrochemical data on Ni, Au, and W electrodes and data on the temperature-dependant G peak shift in CO_2_-derived carbon, surface morphology of the deposited carbons, validation of the quasi-reference electrode potential scale and determination of tungsten content in the electrolyte after electrolysis are given in the Supplementary information. Source data are provided as a Source Data file. Source data are provided with this paper.
