# Supplementary material for: Operando spectroelectrochemical identification of peroxide intermediate in molten carbonate CO2-to-carbon electroreduction
Source: Nat Commun. 2026 Apr 21;17:5513. doi: 10.1038/s41467-026-70977-0 (PMC13287478; doi:10.1038/s41467-026-70977-0)
Supplement: Supplementary file 1 — Supplementary Information [file 41467_2026_70977_MOESM1_ESM.pdf]

Supplementary Information

Operando spectroelectrochemical identification of peroxide intermediate in molten carbonate CO_2-to-carbon electroreduction

Sander Ratso,[1,2*] Michael L. Whittaker,[3,4] Kätlin Kaare,[2] Raluca O. Scarlat [1]

[1] *Department of Nuclear Engineering, University of California, Berkeley, CA 94720, USA*

[2] *National Institute of Chemical Physics and Biophysics, Akadeemia tee 23, Tallinn 12618, Estonia*

[3] *Energy Geosciences Division, Lawrence Berkeley National Laboratory, Berkeley, California 94720, United States*

[4] *Department of Earth & Planetary Sci., UC Berkeley, Berkeley, California 94720, United States*

[*] Corresponding authors: sander.ratso@kbfi.ee (S. Ratso).

Table of Contents

Table of Contents 2
1 Deposition potentials of alkali metals compared to the deposition potential of carbon in pure carbonate electrolytes 3
2 Determination of the theoretical electrochemical window 5
3 Raman signatures of proposed species in carbonate melts 6
4 *Operando* Raman spectroelectrochemistry on Ni, Au and W electrodes 8
4.1 Ni 8
4.2 Au 9
4.3 W 11
5 Determination of the temperature-dependent G peak shift in CO_2-derived carbon 11
6 Surface morphology of the deposited carbons 12
7 Validation of the quasi-reference electrode potential scale 18
8 Determination of tungsten content in the electrolyte after electrolysis 20
9 References 23

1 Deposition potentials of alkali metals compared to the deposition potential of carbon in pure carbonate electrolytes

For carbon to deposit via reaction mechanism 1 (Eq. S1), the metal deposition potential in the melt must be more positive than the deposition potential for carbon (otherwise, carbon will deposit directly before metal deposition happens via Eq. S2, making reaction 1 unlikely)[1].

The thermodynamic carbon deposition potentials are thus defined as the potentials for the reaction:

$$3M_2CO_3 = C + 3M_2O + 2CO_2 + O_2 \quad (S1);$$

and the deposition potentials of metals as the potentials for the reaction:

$$4M_2CO_3 = 4M + 2CO_2 + O_2 \quad (S2).$$

For alkali earth metals, the reactions are defined as follows:

$$2MCO_3 = C + 2MO + CO_2 + O_2 \quad (S3);$$

$$2MCO_3 = 2M + 2CO_2 + O_2 \quad (S4).$$

Supplementary Table 1. Deposition potentials of alkali and alkali earth metals compared to the deposition potential of carbon in pure liquid carbonate electrolytes at 500 °C (according to reactions S1-S4), the melting points of the pure carbonates as well as the potential of direct carbon electroreduction calculated using the HSC Chemistry software. All the potentials are reported vs the $CO_3^{2-}/CO_2–O_2$ redox couple.

**Carbonate salt**	**Metal deposition potential at 500 °C**	**Carbon deposition potential at 500 °C**	**Melting point (°C)**	**Carbon deposition potential at melting point/decomposition temperature**
**Alkali metal carbonates**				
Li_2CO_3	−3.11 V	−1.83 V	723	−1.59 V
K_2CO_3	−2.61 V	−3.19 V	891	−2.74 V
Na_2CO_3	−2.69 V	−2.65 V	851	−2.30 V
**Alkali earth metal carbonates**				

$BeCO_3$	−2.30 V	−0.55 V	54	−0.95 V
$MgCO_3$	−2.52 V	−0.85 V	Decomposes < M.P. (350 °C)	−0.98 V
$CaCO_3$	−3.16 V	−1.31 V	Decomposes < M.P. (850 °C)	−1.05 V
$SrCO_3$	−3.23 V	−1.59 V	Decomposes < M.P. (1100 °C)	−0.70 V
$BaCO_3$	−3.20 V	−1.75 V	Decomposes < M.P. (1740 °C)	−0.94 V
**Direct carbon electroreduction ($CO_2 = C + O_2$)**				
CO_2	-	−1.02 V	-	−1.03 V (723 °C)

The second requirement is that the oxide being formed in the carbon deposition reaction needs to be soluble (for example, successful deposition has been reported in Na-Ba melts, where Ba facilitates the deposition)[2,3]. An alternative reaction has been provided by Licht et al.[4] to explain the apparent deposition of carbon at less negative potentials than expected from eq. S1 in pure lithium carbonate melts:

$$Li_2O + 2CO_2 = C + Li_2CO_3 + O_2 \quad (S5),$$

which was proven to be more consistent with their observed deposition potentials <1 V at increased contents of lithium oxide in the melt.

The third option for carbon deposition is a direct single-carbonate reduction reaction:

$$M_2CO_3 = C + M_2O + O_2 \quad (S6),$$

Supplementary Table 2. Deposition potentials of carbon in pure liquid carbonate electrolytes at 500 °C (according to reaction S6) calculated using the HSC Chemistry software. All the potentials are reported vs the $CO_3^{2-}/CO_2–O_2$ redox couple.

Carbonate salt	Metal deposition potential at 500 °C	Carbon deposition potential at 500 °C	Carbon deposition potential at melting point/decomposition temperature
**Alkali metal carbonates**			
Li_2CO_3	−3.11 V	−1.29 V	−1.21 V
K_2CO_3	−2.61 V	−1.75 V	−1.59 V
Na_2CO_3	−2.69 V	−1.57 V	−1.45 V

2 Determination of the theoretical electrochemical window

In addition to determining the available electrochemical window in the spectroelectrochemical cell via observation of carbon deposition and bubbling, the electrochemical window and electrode potentials were calculated to confirm the source of the bubbling was oxygen evolution. The standard potential for O_2 evolution from oxide oxidation in molten Li,Na,K carbonate via reaction S7 is 2.47 V vs Li^+/Li[5]:

$$2O^{2-} = O_2 + 4e^- \quad \text{(S7).}$$

The Nernst equation to express the potential in this cell would thus be:

$$E_{O^{2-}/O_2} = E^0_{O^{2-}/O_2} + \frac{R*500}{2F}\, ln\frac{a_{O^{2-}}}{\sqrt{PO_2}} \quad \text{(S8).}$$

The upper and lower bounds for the oxide anion activity is directly dependent on the partial pressure of CO_2 due to the equilibrium of autodissociation in carbonate melts:

$$CO_2 + O^{2-} \leftrightharpoons {CO_3}^{2-} \quad \text{(S9).}$$

Over the electrolysis time, it is expected that the CO_2 partial pressure might change somewhat as it is consumed near the surface of the electrolyte, but as the cell is open to the atmosphere via an open

electrode port 1 cm in diameter, there will be a steady supply of CO_2 into the cell. The lower bound for CO_2 partial pressure was thus set to be 0.01% (100 ppm in comparison to the 422 ppm measured in the atmosphere during the time the experiments were conducted[6]) with the upper bound set as 0.1% (1000 ppm). This translates to an oxide anion activity of 0.0219 to 0.0917[7].

The standard potential for C deposition from carbonate decomposition in molten Li,Na,K carbonate is 1.171 V vs Li^+/Li[1], with the potential at 500 °C thus given:

$$E_{CO_3^{2-}/O^{2-}} = E^0_{CO_3^{2-}/O^{2-}} + \frac{R*500}{4F} ln \frac{1}{a_{O^{2-}}} \quad \text{(S10).}$$

With no dissolved oxide anions (low oxygen conditions), it is also possible for the anode reaction to be carbonate-mediated oxygen evolution rather than oxide-based as this reaction normally takes place at a more positive potential of 2.89 V vs Li^+/Li [5]:

$$CO_3{}^{2-} = CO_2 + O_2 + 4e^- \quad \text{(S11).}$$

The overall potential-oxoacidity plot for the cell is thus given in Supplementary Figure 1.

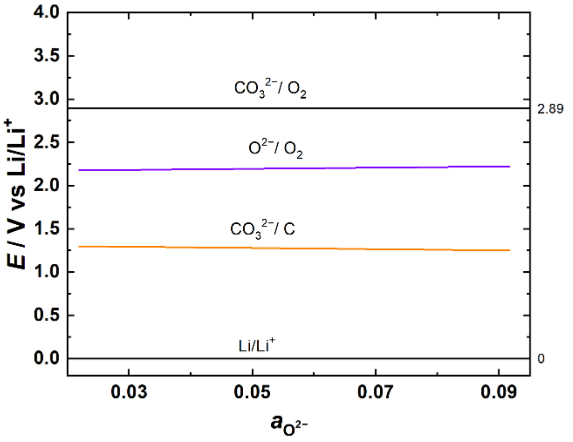

Supplementary Figure 1. Potential-oxoacidity diagram for the eutectic mixture of Li, Na and K carbonate salt at 500 °C at CO_2 partial pressures of 0.01%-0.1%.

3 Raman signatures of proposed species in carbonate melts

Identification of species in the *operando* cell near the surface of the electrode was done by comparison of measured Raman spectra with literature data on Raman spectroscopy of molten carbonates (Supplementary Table 3), specifically studies done in oxygen-rich gas environments and theoretical density functional theory (DFT) studies. In addition to the peaks noted here, the DFT study by Carper

et al.[8] provides shifts for a number of metal-carbonate and metal-peroxide species which may also be possible candidates.

Supplementary Table 3. Raman shifts of proposed species from the literature.

Species	Raman shifts (cm^{-1})	Refs.
CO_3^{2-}	707, 1061, 1390, 1410, 1500, 1762	9,10
O_2^{2-}	642, 690, 832, 919 (NaO_2^{2-}), 949 (LiO_2^{2-})	8,9,11
O_2^{-}	1047-1160	12,13
$C_2O_5^{2-}$	455, 870, 1281, 1391, 1421	10,14
HCO_4^{-}	424, 565, 751, 883, 939, 1350	10
CO_4^{2-}	609, 822, 871, 964	10
NiO	450	11
WC	693, 807	15

For deconvoluting the carbon region of the Raman spectra, a five-component fit as described by Sadezky et al. was used for deconvolution[16] in OriginPro 2024. The bounds for fitting parameters[17] used are given in Supplementary Table 4. Two main peaks dominate the first-order Raman spectra of carbon materials: the G peak near 1580 cm^{-1} and the D1 peak near 1350 cm^{-1} [18]. The G peak arises from C-C bond stretching in sp^2 carbon and the D peak from double-resonance radial breathing mode of hexagonal carbon rings near defects or grain boundaries, specifically arm-chair defects[19,20]. Additionally, three more features are commonly noted: the D2 or D' peak at ~1620 cm^{-1}, the D3 peak at ~1500 cm^{-1} and the D4 peak at ~1180 cm^{-1} [17], which are related to disorder and amorphous/impurity contributions. Several parameters are used to quantify the changes in carbon structures via these peaks: the raw intensity ratios of the D1 and G peak, the width of both the D1 and G peaks, and the position of the G peak, with a vast variety of interpretation methods described for differing allotropes[17–19].

Supplementary Table 4. Bounds for fitting parameters used to fit the experimental Raman spectra, where ω_X is the position of the given peak, FWHM is the full width at half maximum, and I is the intensity.

Parameter	ω_{D1}	ω_G	ω_{D2}	ω_{D3}	ω_{D4}	FWHM	I
Bounds	1300-1400	1550-1600	1590-1630	1400-1550	1050-1300	10-1000	0-∞

4 *Operando* Raman spectroelectrochemistry on Ni, Au and W electrodes

4.1 Ni

The results of *operando* Raman on a Ni electrode are shown in Supplementary Figure 2. The peak at 1061 cm^{-1} corresponding to O-C-O bonds has a higher intensity at no applied potential (Supplementary Figure 2a,b), likely due to the smaller diameter of this electrode and more of the signal coming from the electrolyte as a result. This also reveals nicely the O-C-O bond band at the same region as the deposited carbon materials and an overtone of the out-of-plane bending mode at 1750 cm^{-1} and intensifies the change in the region at 700-850 cm^{-1} as electrolysis is started (Supplementary Figure 2e), the carbonate is reduced, and carbon begins to be deposited. Again, the features at 700 and 832 cm^{-1} change in phase, pointing to a likely common origin. Huang et al. studied the effect of different gas atmospheres as well as the addition of water and Ba/$SrCO_3$ to $(Li\text{-}Na)_2CO_3$ and found CO_4^{2-} and HCO_4^- to be present in addition to CO_3^{2-} in low CO_2/high O_2 conditions and $C_2O_5^{2-}$ in high CO_2 conditions based on *in* situ Raman spectroscopy[10]. In another study, Huang et al. measured the speciation of carbonates during heating up to 923 K and cooling back down to room temperature in both pure argon and oxygen gas. In oxygen, they discovered the appearance of an O_2^{2-}/O_2^- peak at 830 cm^{-1} when the salt was heated, which persisted when the sample was cooled back down to room temperature. As pyrocarbonates have thus far only been seen in carbonates under high CO_2 partial pressures, peroxides are the likelier option. On the Ni electrode, it took somewhat longer (120 s) for a carbon signal to appear (Supplementary Figure 2c, again likely due to a smaller electrode and less carbon being deposited at equivalent potentials compared to the Inconel electrode). The I_D/I_G reached a minimum at 360 s, after which it kept increasing, while $FWHM_D$ and $FWHM_G$ followed a continuous upward trend. Nickel was chosen as a substrate due to its weak affinity for carbide formation ($\Delta G_{M\text{-}C}$) and high solubility of carbon[21], which should lead to a highly sp^2-rich product being formed on the surface of the electrode. However, in this case, the product is amorphous and not very different from the product on the Inconel 600 electrode.

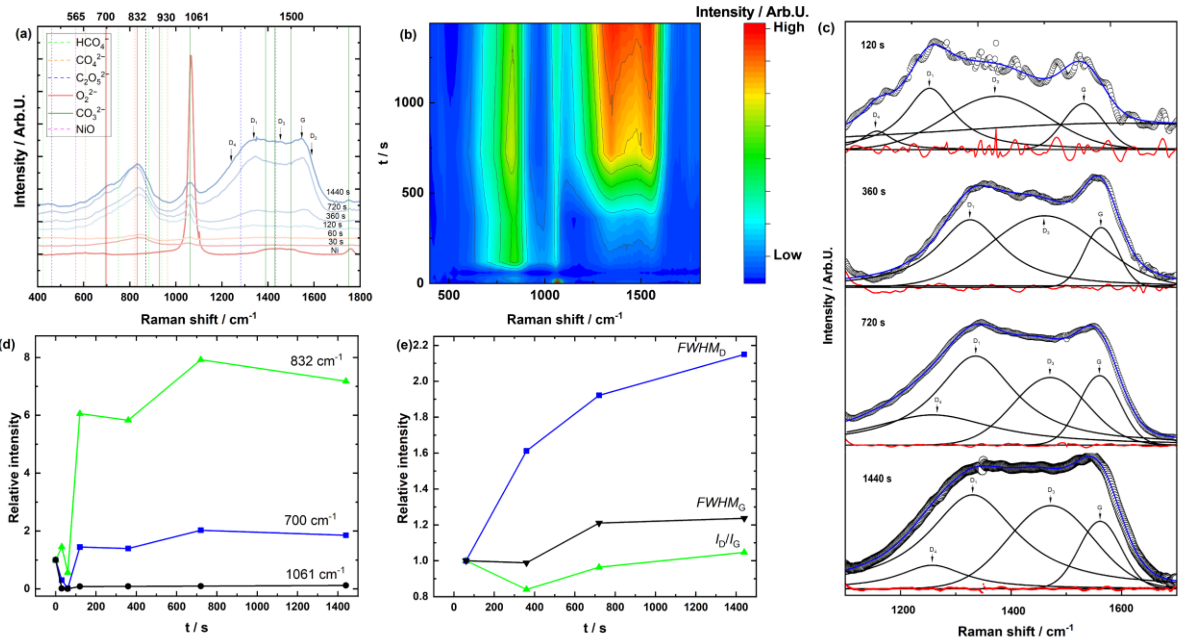

Supplementary Figure 2. (a,b) Stacked Raman spectra of the Ni and $(Li,Na,K)_2CO_3$ interface during electrolysis, (c) deconvoluted carbon regions of the Raman spectra, (d) relative intensities of Raman peaks at specific shifts and (e) I_D/I_G, $FWHM_D$ and $FWHM_G$ derived from the deconvoluted carbon spectra.

4.2 Au

As discussed above, on Au surfaces, CO is preferentially the product of CO_2RR in molten carbonates at low CO partial pressures, but at higher partial pressures C is the product as CO is further reduced. To study the effect of this on the Raman signature of the electrode surface during electrolysis, Au was used as the working electrode (Supplementary Figure 3). As seen from the Raman spectra (Supplementary Figure 3a,b), similar patterns are seen for the Au working electrode. The carbonate-related peaks are reduced (Supplementary Figure 3d) as the electrolysis proceeds and the peroxide/pyrocarbonate-related band becomes more prevalent. In addition to previously seen features, however, there is an additional peak at 930 cm^{-1} which appears only under potential and can also be ascribed either to $C_2O_5^{2-}$, peroxide, or $M\text{-}CO_4^-$ complex species. Interestingly, the feature at 930 cm^{-1} does not change in phase with the features at 700 and 832 cm^{-1} at 1440 s, meaning that there is either interference from another process with a feature at the same Raman shift, or that the origin of this peak is different from the other two. Deconvolution of the Raman spectra of the carbon region is shown in Supplementary Figure 3c. The I_D/I_G ratio in this case reached a minimum of 0.95 at 360 s, after which it increased to 1.45 at 1440 s. The $FWHM_D$ reached a minimum at 120s, while $FWHM_G$ had a maximum at the same time point. As Au also has significant solubility of carbon similarly to Ni and Co, it would be expected that the content of sp^2 carbon in the product would be higher according to the dissolution-precipitation mechanism of carbon deposition, at least at the beginning of the deposition process.

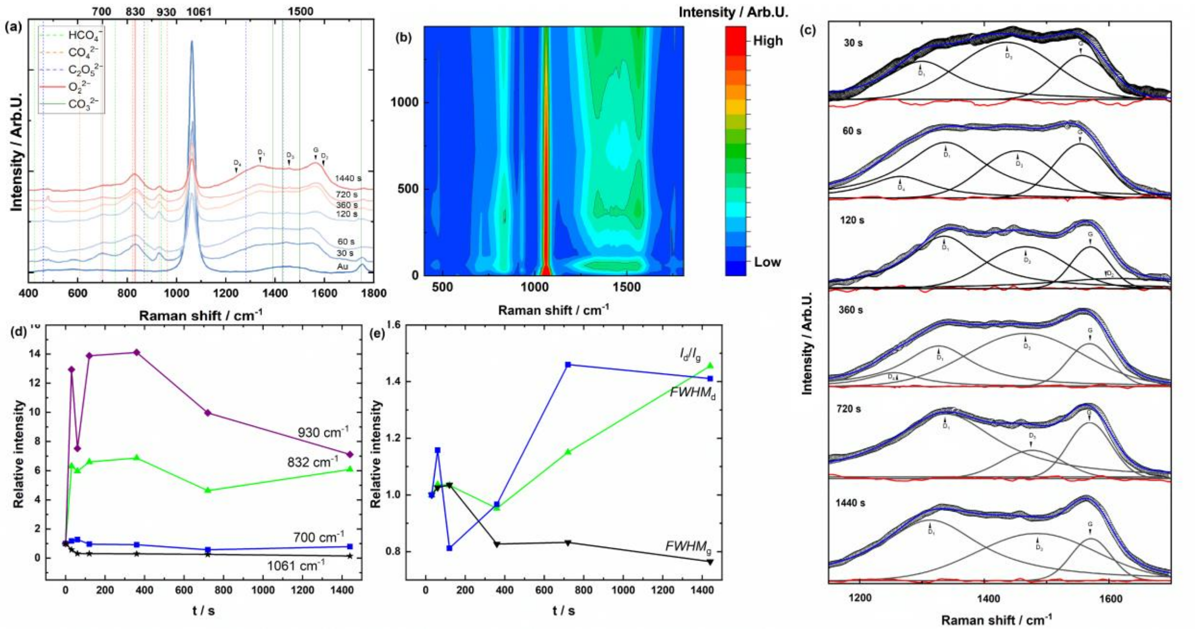

Supplementary Figure 3. (a,b) Stacked Raman spectra of the Au and $(Li,Na,K)_2CO_3$ interface during electrolysis, (c) deconvoluted carbon regions of the Raman spectra, (d) relative intensities of Raman peaks at specific shifts and (e) I_D/I_G, $FWHM_D$ and $FWHM_G$ derived from the deconvoluted carbon spectra.

4.3 W

Supplementary Figure 4 and Movie S1 show the results of *operando* Raman spectroelectrochemistry on a W electrode. The general trends remain the same, as for previous electrodes, with carbonate-related peaks decreasing in intensity as the electrolysis proceeds, and then increasing again as the carbonate is co-deposited alongside the carbon onto the electrode surface. The intensity of the features at 700 and 832 cm^{-1} increased uniformly, while the feature at 930 cm^{-1} did not follow the same trend, confirming the previous observations on Au.

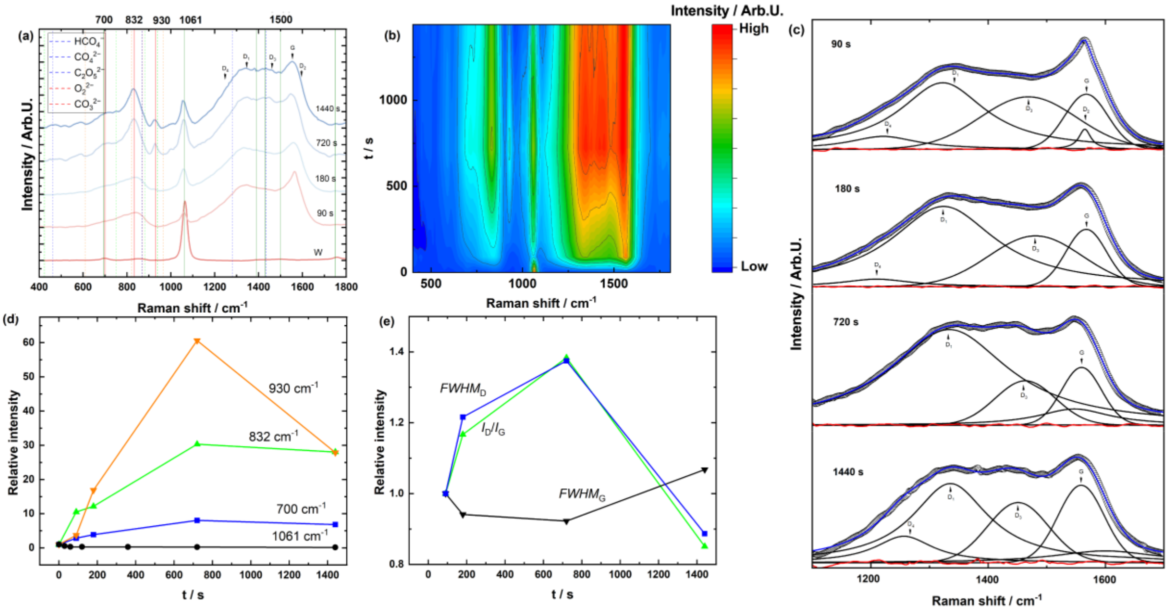

Supplementary Figure 4. (a,b) Stacked Raman spectra of the W and $(Li,Na,K)_2CO_3$ interface during electrolysis, (c) deconvoluted carbon regions of the Raman spectra, (d) relative intensities of Raman peaks at specific shifts and (e) I_D/I_G, $FWHM_D$ and $FWHM_G$ derived from the deconvoluted carbon spectra.

5 Determination of the temperature-dependent G peak shift in CO_2-derived carbon

The position of the G peak has been reported to shift in *sp*^{2}-rich carbon has previously been reported to shift by 0.016 cm^{-1} K^{-1} due to a lengthening of the C-C bonds with temperature[22]. For pyrocarbons, which are more similar in structure (a mixture of *sp*^{2} and *sp*^{3} carbon), a change in G peak Raman shift is more questionable as it has been reported that this type of material experiences no significant shift going from 300 K to 3.8 K[23]. To study the effect of temperature on purified CO_2-derived carbon materials, the as-deposited material was first purified with 1 M HCl, washed and dried. After that, the purified deposit was studied in the same spectroelectrochemical cell used for the original *operando* measurements at room temperature and at 500 °C (Supplementary Figure 5). As can be seen, G peak shifts only by 9 cm^{-1} to 1583 cm^{-1} when it is heated to 500 °C, whereas the as-synthesized material had

a G peak centered at 1558 cm^{-1}. This confirms that temperature effects alone are not responsible for the shift in G peak position.

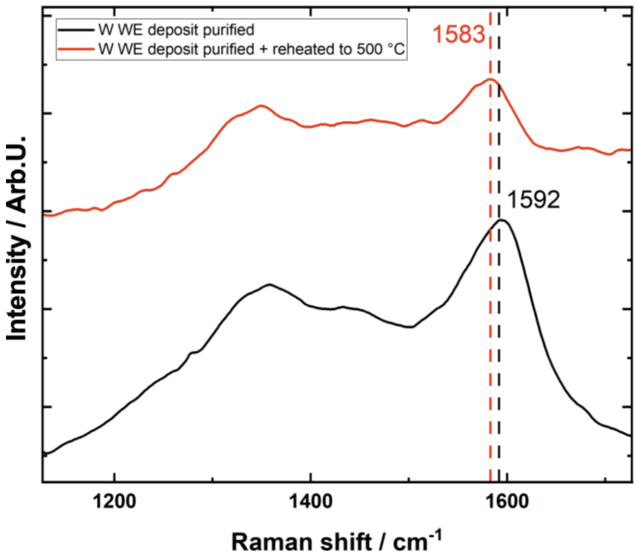

Supplementary Figure 5. Raman spectra of purified, room-temperature carbon deposited using a W electrode from CO_2, and the same material reheated to 500 °C.

6 Surface morphology of the deposited carbons

The surface morphology of the carbon deposits from different cathodes can be seen in Supplementary Figures 6-9. On W cathode, the deposit was a mixture of carbon nano-onions and amorphous carbon (Supplementary Figure 6). In the case of Ni cathode (Supplementary Figure 7), carbon nanotubes and platelet-like carbon appeared in the mixture, whereas for Inconel 600 cathode (Supplementary Figure 8) carbon platelets were the primary product. Finally, for the Au cathode (Supplementary Figure 9), purely amorphous carbon morphology was seen in the deposit via scanning electron microscopy (SEM). Transmission electron microscopy (TEM, Supplementary Figure 10) reveals the structure of the material deposited on the W electrode – a mixture of amorphous areas and graphitic structures (Supplementary Figure 10c-d), with the graphitic edges of the nano-onions especially prominent (Supplementary Figure 10a-b).

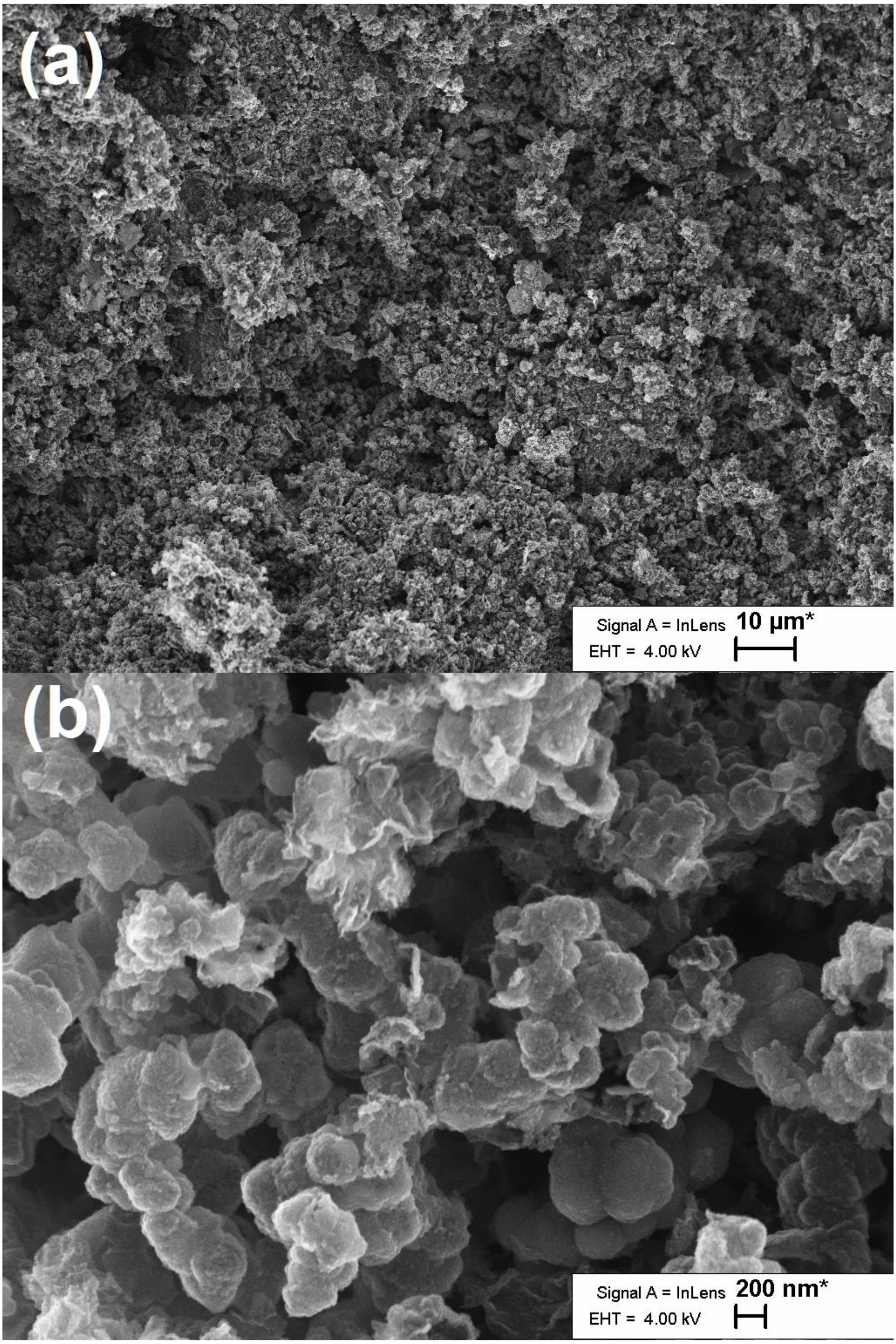

Supplementary Figure 6. Low- (a) and high-magnification (b) scanning electron microscopy images showing the surface morphology of the material deposited on a W electrode.

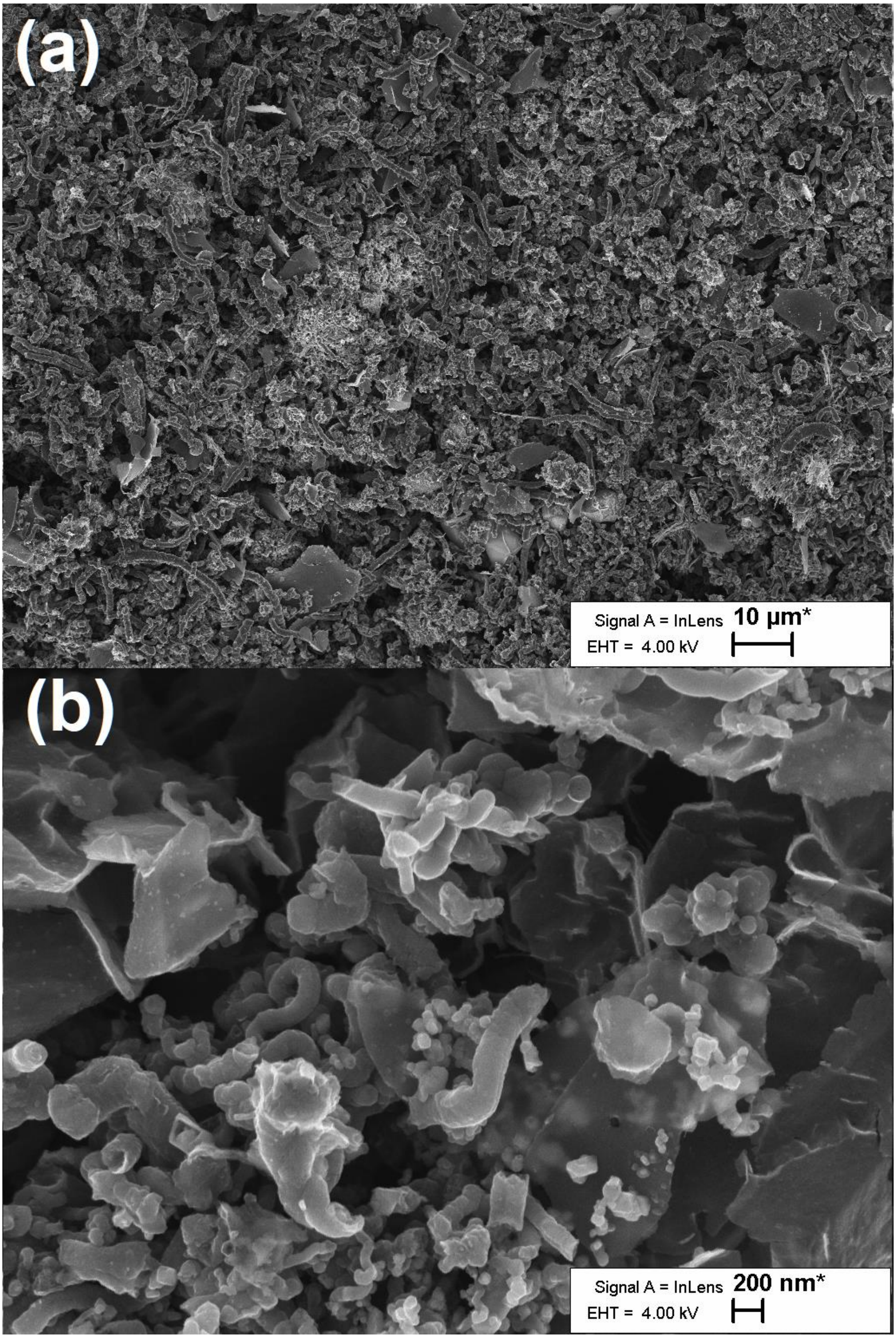

Supplementary Figure 7. Low- (a) and high-magnification (b) scanning electron microscopy images showing the surface morphology of the material deposited on a Ni electrode.

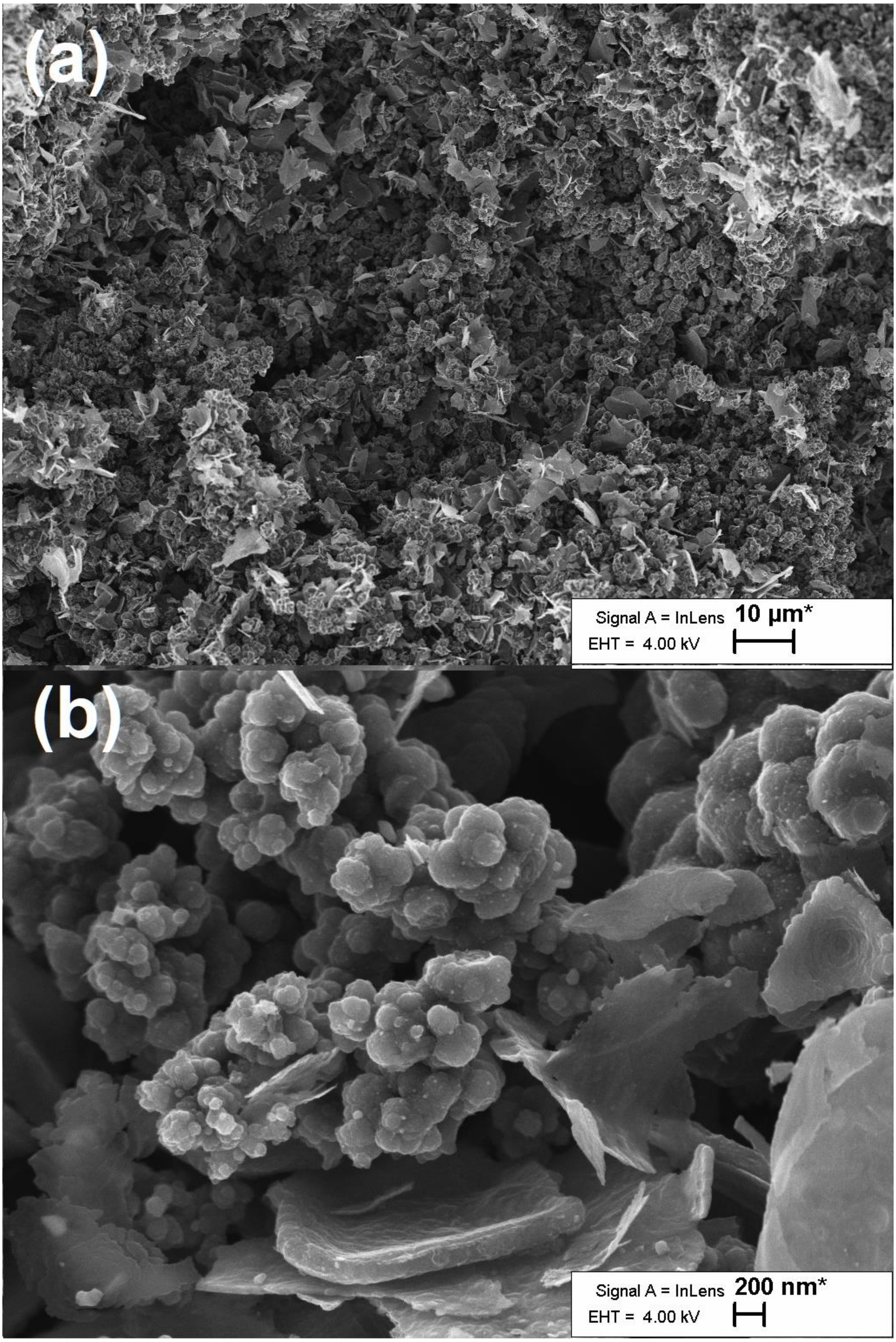

Supplementary Figure 8. Low- (a) and high-magnification (b) scanning electron microscopy images showing the surface morphology of the material deposited on an Inconel 600 electrode.

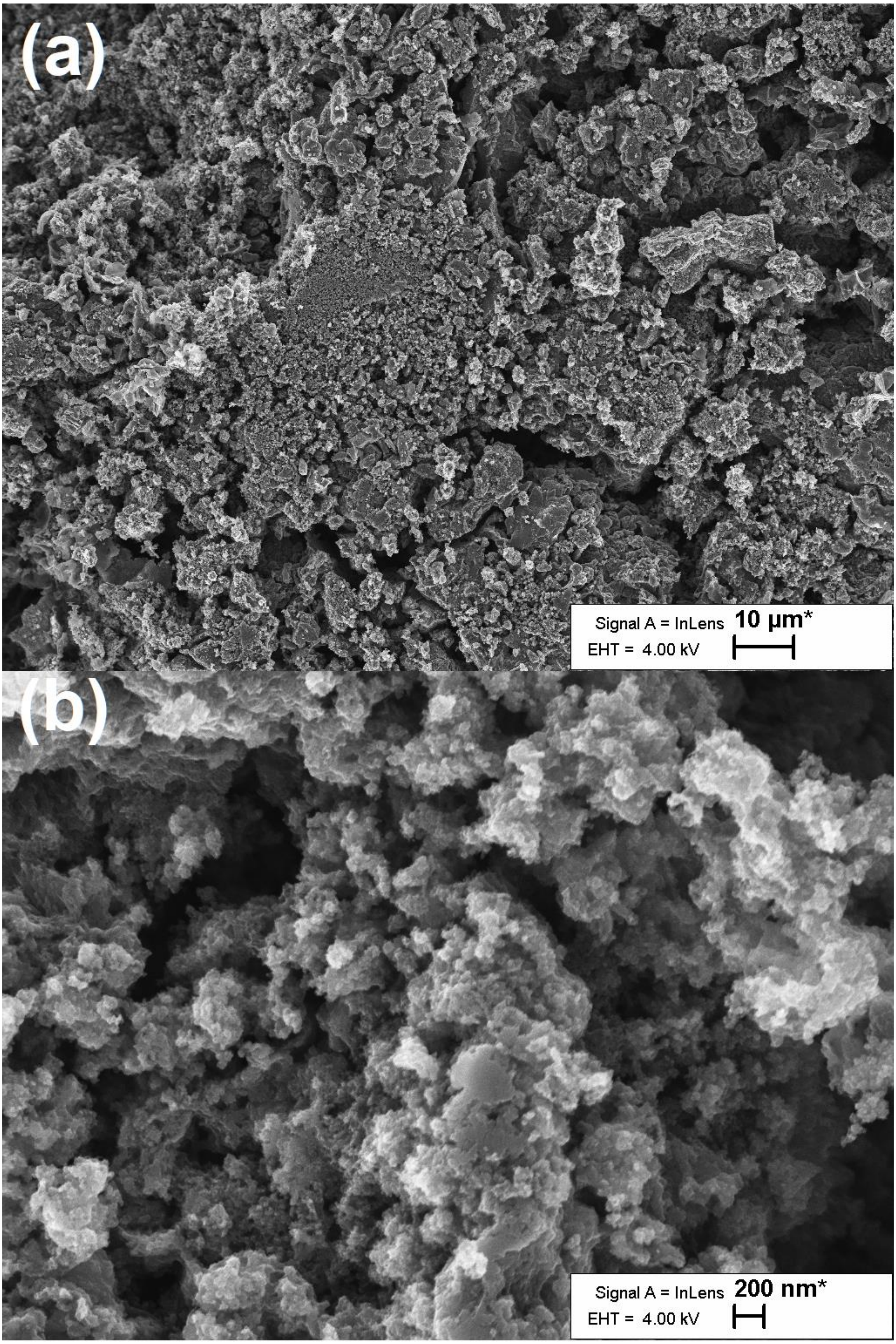

Supplementary Figure 9. Low- (a) and high-magnification (b) scanning electron microscopy images showing the surface morphology of the material deposited on an Au electrode.

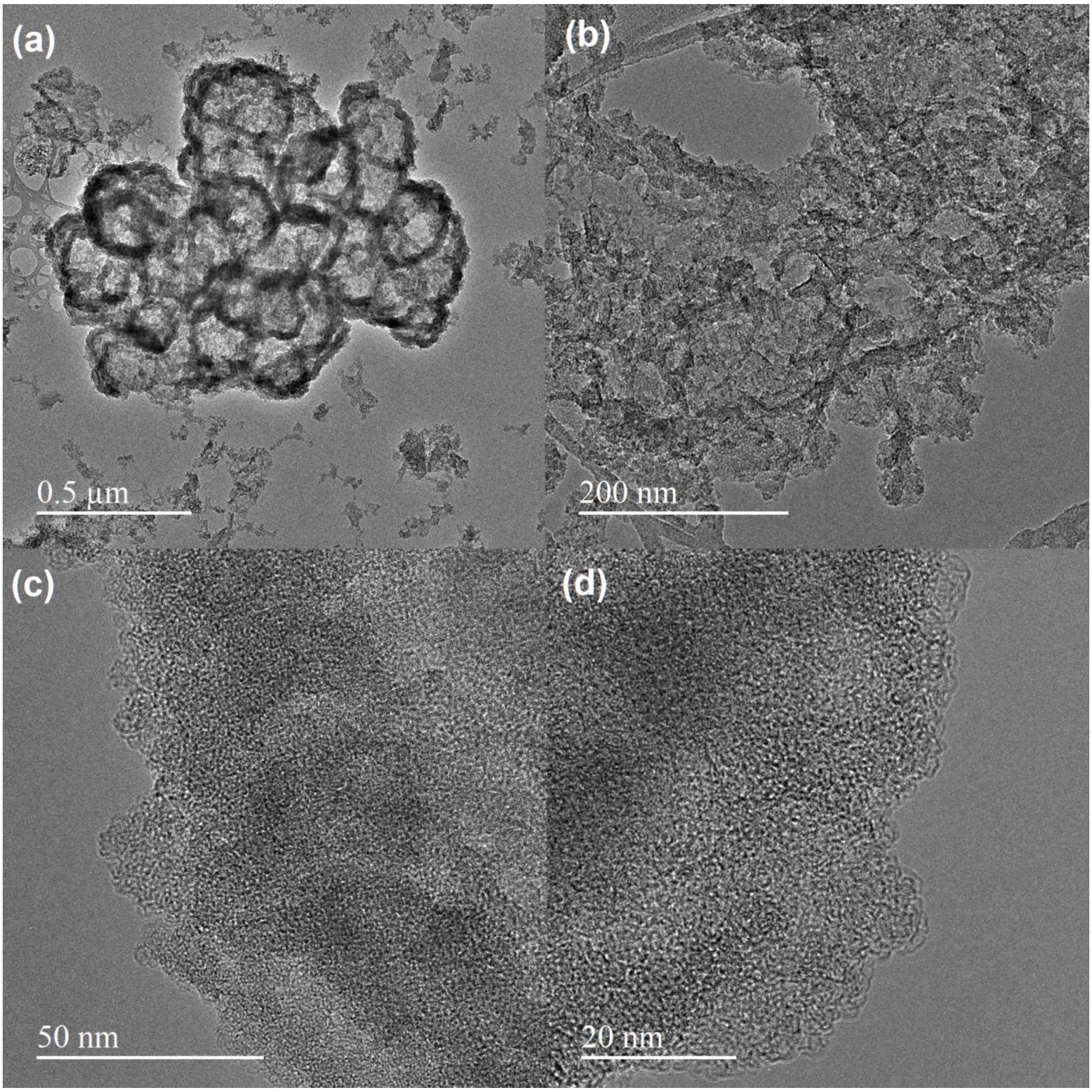

Supplementary Figure 10. Transmission electron microscopy images showing the structure of the material deposited on a W electrode.

7 Validation of the quasi-reference electrode potential scale

To confirm the validity of the W QRE and correlate it with more stable reference systems, we performed comparative experiments using both Ni and Ag as the reference electrodes (Supplementary Figures 11 and 12).

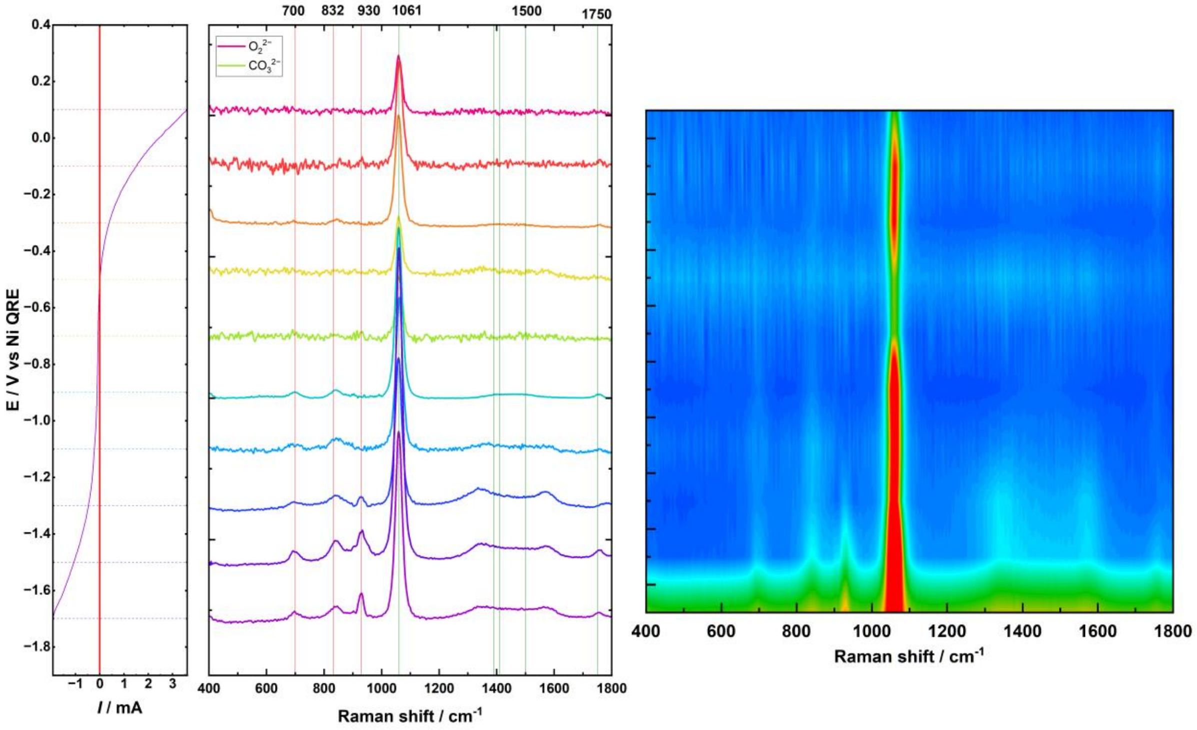

Supplementary Figure 11. Potential-dependent Raman spectra of the interface between a W electrode and the eutectic (Li,Na,K)$_2$CO$_3$ salt mixture during a linear sweep from 0.1 V to −1.7 V vs Ni QRE. The LSV is presented as-measured and is not iR-corrected.

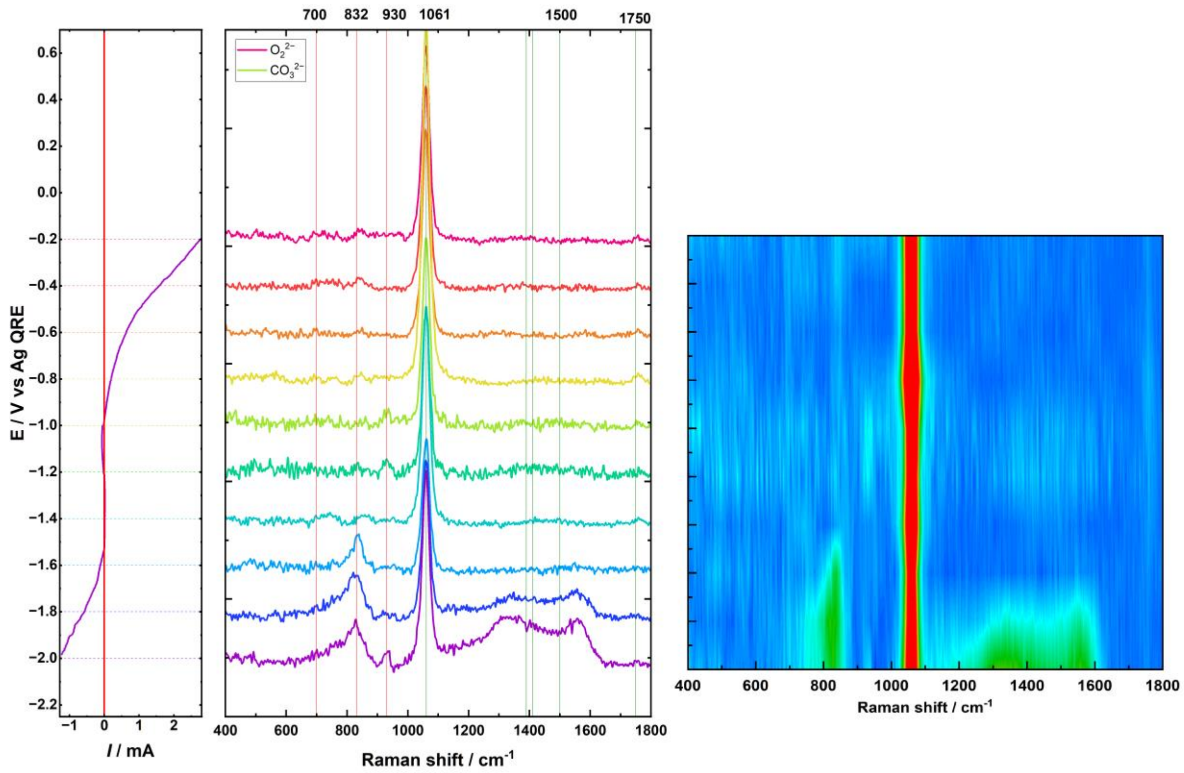

Supplementary Figure 12. Potential-dependent Raman spectra of the interface between a W electrode and the eutectic $(Li,Na,K)_2CO_3$ salt mixture during a linear sweep from −0.2 V to −2 V vs Ag QRE. The LSV is presented as-measured and is not iR-corrected.

As can be seen, the observations regarding the peroxide intermediate stay the same. Concurrently with reduction current, a feature centred at ~830 cm^{-1} appears, and as the electrode is further polarized and the reduction current increases, peaks according to carbon appear in the Raman spectrum. The overall potentials are shifted as expected, but the shape of the LSV, as well as the separation between the OER and carbon deposition remain the same.

8 Determination of tungsten content in the electrolyte after electrolysis

To confirm that the anodic reaction seen in the CV on Figure 1a is oxygen evolution and not the anodic dissolution of tungsten, a potential of 0.4 V vs W QRE was applied to the working electrode for 1440 s (Supplementary Figure 13).

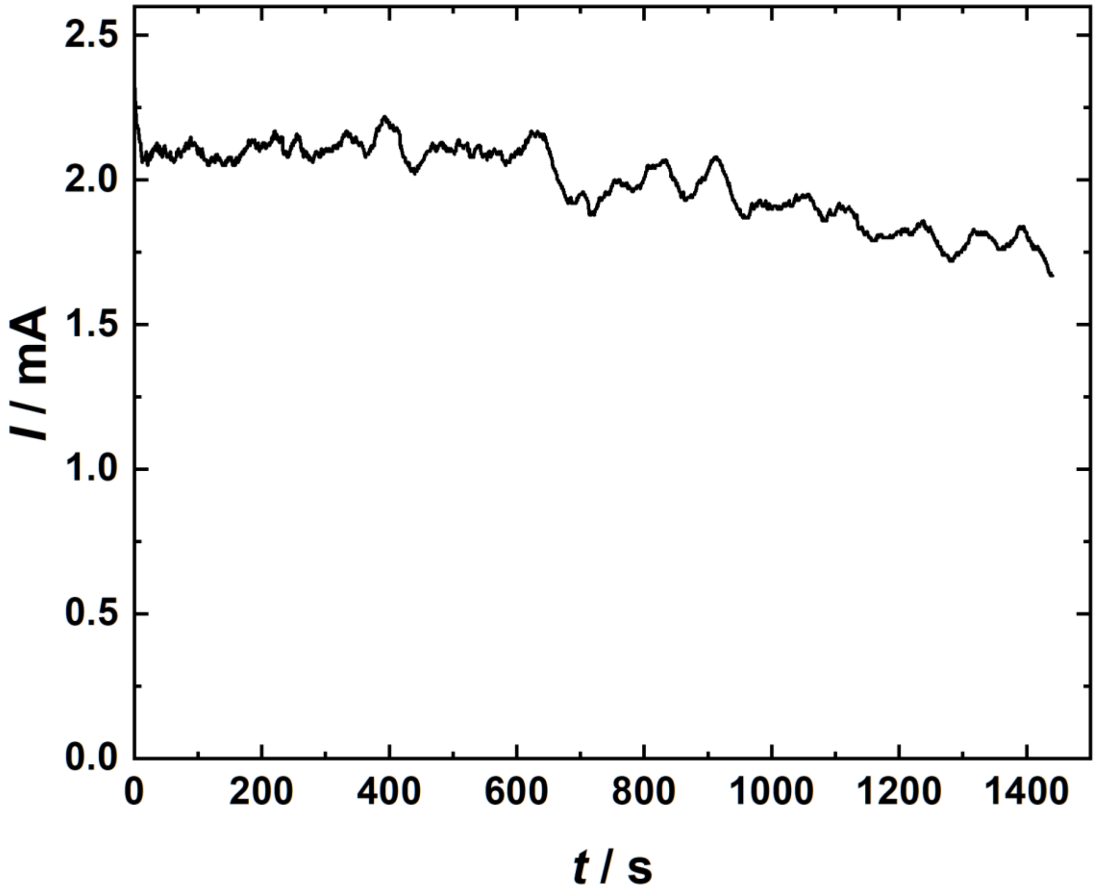

Supplementary Figure 13. Current response over 1440 s of a CO_2 electrolysis reactor with an applied potential on the working electrode of 0.4 V at 500 ºC. The working, counter and reference electrodes were W.

After this the reactor was cooled down and the electrodes removed from it, the salt was sampled by scraping it from the areas where the electrodes were during the measurement. 10 mg of this salt was then weighed into a vial and digested using a mixture of 450 µl of concentrated HCl (>37%, p.a., Fluka) and 50 µl of a 100 ppm Ga internal standard in concentrated HNO_3. After full digestion of the salt, the samples were analysed using total reflection X-ray fluorescence. For comparison, a fresh Li-Na-K carbonate mixture and a Li-Na-K carbonate mixture to which 1 wt.% of WO_3 was added (to get 1 wt.% of W in the salt) were also analysed. The results are given in Supplementary Table 5, with the digestion efficiency of W confirmed as 93.27% via digestion of the 1 wt.% W salt.

Supplementary Table 5. W content as-determined by TXRF in pristine Li-Na-K carbonate, Li-Na-K carbonate with 1.27 wt.% of WO_3 was added (which accords to 1 wt.% W).

Sample	W (ppm)
**Pristine (Li-Na-K)$_2$CO$_3$**	0
**(Li-Na-K)$_2$CO$_3$ + 1.27 wt.% WO$_3$**	9327
**(Li-Na-K)$_2$CO$_3$ after 1440 s electrolysis**	354

As can be seen, while the salt after electrolysis contains some W, the content is very low. In an oxo-basic melt at a temperature of 500 ^{o}C, formed WO_3 would be expected to form soluble Li_2WO_4 via the reaction $WO_3 + Li_2O = Li_2WO_4$. The mass of the salt prior to electrolysis was 62.3 mg. Using the total charge passed during the electrolysis (Supplementary Figure 14, 2.86 C total), and considering a 6-electron oxidation of W, we find that a mass of 0.908 mg of W would be expected to be oxidized off the electrode (considering a Faradaic efficiency of 100%), which would correspond to 14686 ppm of W in the electrolyte. This is considerably more than the 354 ppm detected, which would accord to 2.41% of the current, proving that the reaction cannot be tungsten oxidation.

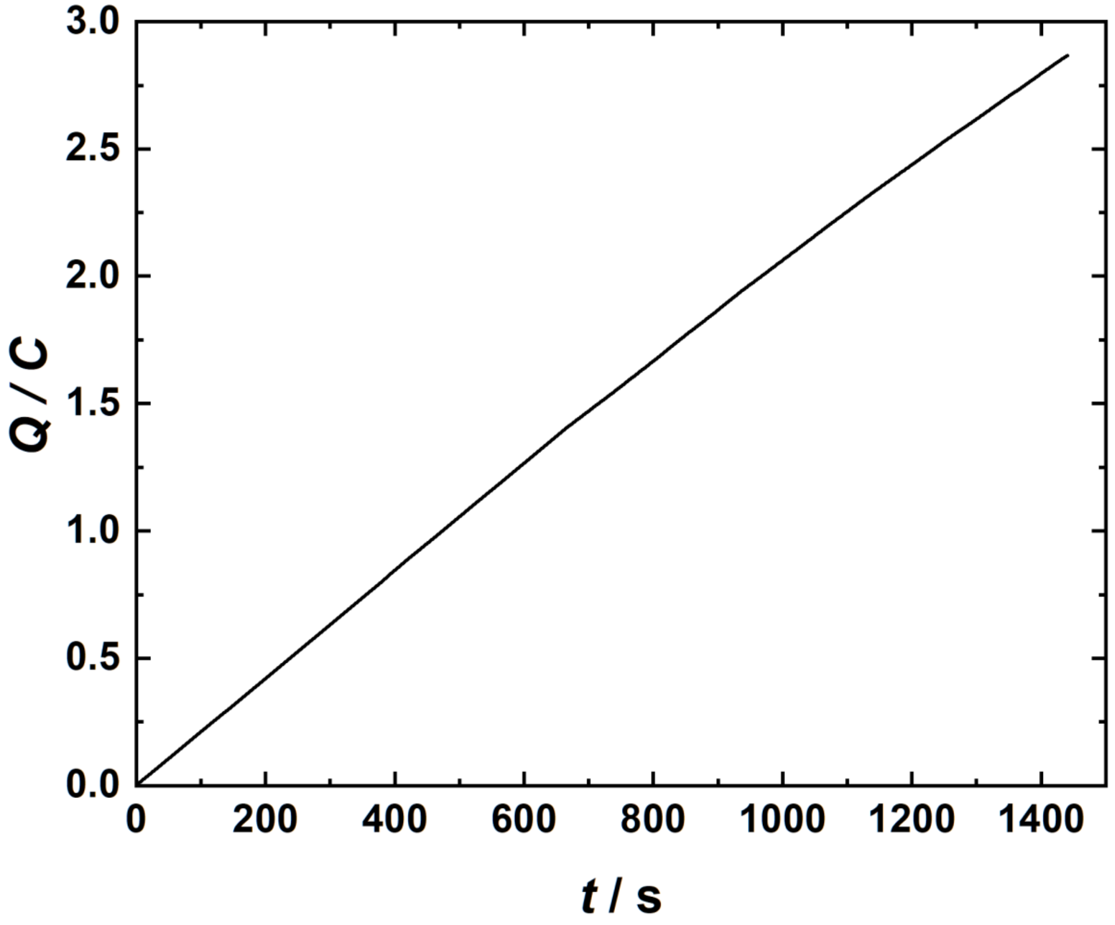

Supplementary Figure 14. Charge passed during CO_2 electrolysis calculated from the data on Supplementary Figure 13.

Thus, the anodic dissolution of W remains unlikely. A more likely explanation is the partial oxidation/dissolution of W as the salt becomes molten (explaining the low W content in the melt), and the formation of a thin, compact WO_3 film (likely the nature of the Ox_1 peak on the W cyclic voltammogram on Figure 1a), that can support OER while inhibiting further W dissolution under anodic polarization. This is consistent with both the TXRF and polarization curves.

9 References

1. Weng, W., Tang, L. & Xiao, W. Capture and electro-splitting of CO2 in molten salts. *Journal of Energy Chemistry* **28**, 128–143 (2019).
2. Ren, J., Lau, J., Lefler, M. & Licht, S. The Minimum Electrolytic Energy Needed to Convert Carbon Dioxide to Carbon by Electrolysis in Carbonate Melts. *Journal of Physical Chemistry C* **119**, 23342–23349 (2015).
3. Wang, X., Licht, G., Liu, X. & Licht, S. CO2 Utilization by Electrolytic Splitting to Carbon Nanotubes in Non-Lithiated, Cost-Effective, Molten Carbonate Electrolytes. *Advanced Sustainable Systems* **6**, 2100481 (2022).
4. Ren, J., Li, F. F., Lau, J., González-Urbina, L. & Licht, S. One-Pot Synthesis of Carbon Nanofibers from CO<inf>2</inf>. *Nano Letters* **15**, 6142–6148 (2015).
5. Li, X. *et al.* Tailoring Borate Mediator Species Enables Industrial CO Production with Improved Overall Energy Efficiency by Sustainable Molten Salt CO2 Electrolysis. *Advanced Science* **12**, 2406457 (2025).
6. Global Monitoring Laboratory. Trends in CO2 - NOAA Global Monitoring Laboratory. https://gml.noaa.gov/ccgg/trends/index.html.
7. Chen, D. *et al.* A dual-electrode oxide ion sensor for molten carbonates. *Journal of Electroanalytical Chemistry* **925**, 116900 (2022).
8. Carper, W. R., Wahlbeck, P. G. & Griffiths, T. R. DFT Models of Molecular Species in Carbonate Molten Salts. *J. Phys. Chem. B* **116**, 5559–5567 (2012).
9. Chen, L.-J., Lin, C.-J., Zuo, J., Song, L.-C. & Huang, C.-M. First Spectroscopic Observation of Peroxocarbonate/ Peroxodicarbonate in Molten Carbonate. *J. Phys. Chem. B* **108**, 7553–7556 (2004).
10. Zhang, P., Wu, T. & Huang, K. Identification of Active Surface Species in Molten Carbonates Using in situ Raman Spectroscopy. *Front. Energy Res.* **9**, (2021).
11. Chen, L.-J., Cheng, X., Lin, C.-J. & Huang, C.-M. In-situ Raman spectroscopic studies on the oxide species in molten Li/K2CO3. *Electrochimica Acta* **47**, 1475–1480 (2002).

12. Fischer, D., Zagorac, D. & Schön, J. C. The presence of superoxide ions and related dioxygen species in zinc oxide—A structural characterization by in situ Raman spectroscopy. *Journal of Raman Spectroscopy* **53**, 2137–2146 (2022).

13. Hester, R. E. & Nour, E. M. Resonance Raman studies of transition metal peroxo complexes: 4-The potassium μ-Peroxo-bis[pentacyanocobaltate(III)], K6[(CN)5CoO2Co(CN)5]·H2O and μ-superoxo-bis-[pentacyanocobaltate(III)], K5[(CN)5CoO2Co(CN)5]·H2O complexes. *Journal of Raman Spectroscopy* **11**, 43–48 (1981).

14. Zhang, L. *et al.* First spectroscopic identification of pyrocarbonate for high CO2 flux membranes containing highly interconnected three dimensional ionic channels. *Phys. Chem. Chem. Phys.* **15**, 13147–13152 (2013).

15. Hussain, S., Abbas Zaidi, S., Vikraman, D., Kim, H.-S. & Jung, J. Facile preparation of tungsten carbide nanoparticles for an efficient oxalic acid sensor via imprinting. *Microchemical Journal* **159**, 105404 (2020).

16. Sadezky, A., Muckenhuber, H., Grothe, H., Niessner, R. & Pöschl, U. Raman microspectroscopy of soot and related carbonaceous materials: Spectral analysis and structural information. *Carbon* **43**, 1731–1742 (2005).

17. Brubaker, Z. E., Langford, J. J., Kapsimalis, R. J. & Niedziela, J. L. Quantitative analysis of Raman spectral parameters for carbon fibers: practical considerations and connection to mechanical properties. *J Mater Sci* **56**, 15087–15121 (2021).

18. Schuepfer, D. B. *et al.* Assessing the structural properties of graphitic and non-graphitic carbons by Raman spectroscopy. *Carbon* **161**, 359–372 (2020).

19. Thapliyal, V., Alabdulkarim, M. E., Whelan, D. R., Mainali, B. & Maxwell, J. L. A concise review of the Raman spectra of carbon allotropes. *Diamond and Related Materials* **127**, 109180 (2022).

20. Sasaki, K., Tokura, Y. & Sogawa, T. The Origin of Raman D Band: Bonding and Antibonding Orbitals in Graphene. *Crystals* **3**, 120–140 (2013).

21. Yu, R., Du, K., Deng, B., Yin, H. & Wang, D. Unraveling the role of substrate materials in governing the carbon/carbide growth of molten carbonate electrolysis of CO 2. *Nanoscale* **15**, 18707–18715 (2023).

22. Calizo, I. *et al.* Raman nanometrology of graphene: Temperature and substrate effects. *Solid State Communications* **149**, 1132–1135 (2009).

23. Mallet-Ladeira, P. *et al.* A Raman study to obtain crystallite size of carbon materials: A better alternative to the Tuinstra–Koenig law. *Carbon* **80**, 629–639 (2014).
