# Supplementary material for: Operando spectroelectrochemical identification of peroxide intermediate in molten carbonate CO2-to-carbon electroreduction
Source: Nat Commun. 2026 Apr 21;17:5513. doi: 10.1038/s41467-026-70977-0 (PMC13287478; doi:10.1038/s41467-026-70977-0)
Supplement: Supplementary file 2 — Description of Additional Supplementary Files [file 41467_2026_70977_MOESM2_ESM.pdf]

Description of Additional Supplementary Files

File Name: Supplementary Movie 1

Description: Oxygen evolution and carbon deposition on a W electrode in a molten eutectic mixture of Li, Na and K carbonates at 500 °C.
