# Supplementary material for: Operando spectroelectrochemical identification of peroxide intermediate in molten carbonate CO2-to-carbon electroreduction
Source: Nat Commun. 2026 Apr 21;17:5513. doi: 10.1038/s41467-026-70977-0 (PMC13287478; doi:10.1038/s41467-026-70977-0)
Supplement: Supplementary file 4 — Transparent Peer Review file [file 41467_2026_70977_MOESM4_ESM.pdf]

nature portfolio

Peer Review File

Operando spectroelectrochemical identification of peroxide intermediate in molten carbonate CO_2-to-carbon electroreduction

Corresponding Author: Dr Sander Ratso

This file contains all reviewer reports in order by version, followed by all author rebuttals in order by version.

Version 0:

Reviewer comments:

Reviewer #1

(Remarks to the Author)
The reduction mechanism of CO2/carbonate in high temperature molten carbonate is significantly important for developing a value-added CO2 capture and utilsation technology, however, the mechanism study is very challenging due to the lack of high temperature opperando methods as well as the harsh and complicated reaction environment. On the aspect, the current work is an initiative of applying spectro-electrochemistry for operando observations of CO2 reduction in molten carbonate. A carbon-adsorbed peroxide intermediate was determined under the specific experimental conditions by the authors, but the redcution mechanism may be affected by various experimental parameters such as temperature, the electrolyte composition, CO2 partial pressure, cathode materials as well as applied current density. More experiments shall be done to get an universal conclusion. Of course, this work is meaningful to the field of high temperature electrochemisty and CO2 reduction in high temperature molten slats. It could be accepted for publication after addressing following concerns.
1. W was used as the reference electrode in this work, however, it is not stable enough and have to be corrected with other electrodes such as Ag+/Ag, NiO/Ni.
2. The reliability of the experimental data and their explanations have to be further checked. For example, the on-set potential for OER is very close according to the CVs on different electrodes in the molten carbonate. This might not be the truth, for Figure 1a, the anodic current (OER) shall be the anodic dissolution of W rather than oxygen evolution, although gas bubble was observed in the video. The gas bubbles might be the volatite WO3 and/or CO2 due to the reation W +3 CO3(2-) = WO3 + 3CO2.
3. More RS tests shall be conducted under different CO2 partial pressure and polarization conditions to confirm the proposed mechanism.
4. Was surface enhanced effect observed on Au electrode at the high temperature?

Reviewer #2

(Remarks to the Author)
This is an important study presenting the first real-time in-situ spectroscopic analysis of a useful decarbonization process to mitigate the ongoing buildup of the greenhouse gas CO2. There are several inconsistencies and as delineated below major revision is needed. Subsequent to the appropriate major revision, and another review, the manuscript is recommended for publication.
1) The implication in the title that operando molten carbonate measurements have not been previously made is incorrect, and it should be corrected in the title,
1a) Title: Correct the title by addition of the qualifying underlined term:
1a) “A first Spectroelectrochemical Operando Probing of Carbon Reduction from Direct Air Capture in Molten Carbonate, “
1b) On a related note, the main text should be corrected to include references to prior operando molten carbonate measurements. That is, change the following phrase to include previous operando examples by adding the underlined text and references,
Change from: “,… while progress in looking at the mechanistic details of this process remains limited due to a lack of tools for operando investigation of the process.”
to: “. Operando measurements of cyclic voltammetry, current steps, CO2 and O2 concentrations have been made (in real time) during carbon reduction in molten carbonate.a-c However, progress on the mechanistic details of this process remains

limited due to a lack of tools for spectroscopic operando investigation of the process.
a. Licht, S.; B. Wang; S. Ghosh; H. Ayub; D. Jiang; Ganley, J. New Solar Carbon Capture Process: STEP Carbon Capture. J. Phys. Chem. Lett. 2010, 1, 2363-2368.
b. Ren, J.; Johnson, M.; Singhal, R.; Licht, S. Transformation of the Greenhouse Gas CO_2 by Molten Electrolysis into a Wide Controlled Selection of Carbon Nanotubes. J. CO_2 Util. 2017, 18, 335-344.
c. Ren Wang, X.; Liu, X.; Licht, G.; Wang, B.; Licht, S. Exploration of Alkali Cation Variation on the Synthesis of Carbon Nanotubes by Electrolysis of CO_2 in Molten Carbonates. J. CO_2 Util. 2019, 18, 303-312.

2) It would be useful for the researchers to strengthen their results that peroxide, rather than oxide or superoxide, is a reaction intermediate to carbon reduction. This new study does an excellent job of correlating the presence of peroxide with carbon growth. However, is it alternatively possible that this result might be interpreted that oxide or superoxide could be a principal species in the carbon reaction?
This is because in molten salts there is a dynamic equilibrium between oxides, peroxide and superoxides:
2O2- + O2 <-> O22-; O22- + O2 <-> 2O2-
In molten carbonates, the dominate species of the three has been well studied, varies, and is a strong function of the alkali or alkali earth cation and temperature of the carbonate melt. Table 2 of the supplementary information apparently notes that peroxide, but not oxide or superoxide, can be identified by this study's Raman spectroscopy in carbonate melts. Hence, while peroxide was measured, it might only be present as a consequence of the dynamic equilibria, and is it possible instead that either oxide or superoxide is the principal oxygen intermediate, growing by equilibrium with peroxide and directly participating in the carbon reduction mechanism? This question is compelling in light of the high potential noted in the manuscript that is necessary to complete the reaction of peroxide to oxide (equation 6).

3) The phase: "At temperatures above 500 °C in oxygen-rich environments (such as air), amorphous carbon can start to be oxidized in a mixture of carbonates and solid carbon33, but" is misleading as causing a temperature measurement limitation to the present study.
The carbon product grown at the cathode is protected against oxidation by the applied electrolysis cathodic bias, and hence not subject to oxidation by the hot O2 generated by the anode. This is evidenced by the high coulombic efficiency (approaching 100%) demonstrated at 770°C in the study:
Licht G.; Peltier, E.; Gee, S.; Licht, S. Eliminating active CO2 concentration in Carbon Capture and Storage (CCUS): Molten carbonate decarbonization through an insulation/diffusion membrane, Decarbon 2025, 7, 1000944.

4) continuation of that manuscript phrase "33, but the temperature must be somewhat above the melting point of the salt to induce faster mass transfer, which is why the working temperature was set to be as high as possible without potentially oxidizing the deposited carbon." As discussed above, the protected carbon product molten carbonate working temperature is not limited to 500°C; this leads to another question, why was the spectroscopic analysis conducted only at 500°C? The more significant graphene nano-carbon allotrope CO2 reduction products are formed at a working temperature above 650°C (Liu, X.; Licht, G.; Wang, X.; Licht, S. Controlled Growth of Unusual Nanocarbon Allotropes by Molten Electrolysis of CO2. Catalysts 2022, 12, 125) and perhaps are dominated by a different carbon reduction mechanism (although this 500°C study is a very useful start). On that note, it would also be useful to include SEM and TEM in the paper or SI characterizing the carbon product grown in the study.

5a) Abstract: While, a great deal needs to be learned or reaffirmed about the underlying reaction of molten carbonate CO2 reduction, much is already known, and the qualitative terms 'ill' and 'suffer' in the abstract diminish objectivity (they are "ill"-advised). "ill" and 'suffer' qualitatively suggest that the foundation of understanding of the mechanism of molten carbonate reduction is less advanced than it actually is. Please revise that sentence in the abstract.
5b) On a related note, in the text of the main manuscript consider adding the following few sentences, to present a foundation of understanding of the underlying reaction mechanisms of the electrolysis of CO2 in molten salts.
Add: "The individual reactions occurring at the anode and cathode are still being explored.d However, a current understanding of the reduction mechanism of CO2 to carbon in molten carbonates includes (i) the rapid, exothermic reaction of gas phase CO2 with oxide in the electrolyte to form carbonate, (iia) the simultaneous reduction of cathodic carbonate to form carbon and (iib) anodic oxidation of oxide to form oxygen, for a net reaction of CO2 -> C + O2.e Transition metal nucleation at the cathode drives the formation of specific graphene nano-carbon morphologies, and root or tip growth of the carbon at the cathode have been demonstrated.f High iron or nickel drives carbide formation and magnetic CNT formation,g while high current density, and additives causing heptagonal, rather hexagonal carbon building blocks generate torsional effects causing the formation of helical graphene nano-allotropes.h Inhibition of transition metal nucleation suppresses 1D symmetry CNT growth and facilitates 0D symmetry carbon nano-onion,i 2D graphene and carbon nano-platelet,j and 3D carbon nano-scaffold growth.k
d. Ren, J.; Lau, J.; Lefler, M.; Licht, S.; The minimum electrolytic energy needed to convert carbon dioxide by electrolysis in carbonate melts. J. Phys. Chem C 2015, 119, 23342-23349
e. Ren, J.; Yu, A.; Peng,P.; Lefler, M.; Li, F.-F.; S. Licht, Recent advances in solar thermal electrochemical process (STEP) for carbon neutral products and high value nanocarbons. Acc. Chem. Res. 2019, 52, 3177–3187.
f. Johnson, M.; Ren, J.; Lefler, M.; Licht, G.; Vicini, J.; Liu, X.; Licht, S. Carbon Nanotube Wools Made Directly from CO_2 by Molten Electrolysis: Value-Driven Pathways to Carbon Dioxide Greenhouse Gas Mitigation. Mater. Today Energy 2017, 5; 230-236.
g. Wang, X.; Sharif, F.; Liu, X.; Licht, G.; Lefler, M.; Licht, S. Magnetic carbon nanotubes: carbide nucleated electrochemical growth of ferromagnetic CNTs, J. CO_2 Util. 2020, 40, 101218.
h. Liu, X.; Ren, J.; Wang, X.,; Licht, S, Carbon Nano Onions Made Directly from CO_2 by Molten Electrolysis for Greenhouse Gas Mitigation. Adv. Sustain. Syst. 2019, 3, 1900056
i. Liu, X.; Licht, G.; Wang, X.; Licht, S. (2021). The green synthesis of exceptional braided, helical carbon nanotubes and

Nanospiral Platelets Made Directly from CO_2. Mater. Today Chem. 2021, 22, 100529.
j. Liu, X.; Wang, X.; Licht, G.; Licht, S. Transformation of the Greenhouse Gas Carbon Dioxide to Graphene. J. CO_2 Util. 2020, 236, 288-294.
k. Wang, X.; Licht, G.; Liu, X.; Licht, S. One Pot Facile Transformation of CO_2 to an Unusual 3 D Nano-scaffold Morphology of Carbon. Sci. Rep. 2020, 10, 21518.

6) To emphasize the fundamental and climate change importance of the study expand the following text sentence by considering adding the underlined terms to demonstrate the varied reported nanocarbon allotropes products that have been synthesized:
"By CO2MSE the synthesis of varied nanocarbon products, including thin, wool, magnetic, helical, nano-bamboo, nano-pearl, branched and macro-assemblies of carbon nanotubes13, nano-platelets and graphene14, doped carbons,15,16,c , carbon nano-onions, carbon nano-scaffolds and even oxide-carbon composites17 have been demonstrated in separate studies by the Licht group. It is also useful to reference that prior to references 15 and 16, doped CNTs were reported at: Ren, J.; Johnson, M.; Lefler, M.; Licht, G; Vicini, J.; Licht, S. Data on SEM, TEM, and Raman Spectra of Doped, and 35,36 Carbon Nanotubes Made Directly from CO_2 by Molten Electrolysis. Data in Brief 2017, 14, 592-606.

7) Revisions needed related to Table S1:
7a) Please add rows for the deposition potentials of alkali earth metals from carbonates (Be, Mg, Ca, Sr, Ba carbonates) to the table (as stated in the table legend) and add a row with the important comparative CO2 to C +O2 carbon deposition potential, When discussing the latter refer to the calculation that the CO2 to C + O2 deposition potential is not significantly temperature dependent (reference: Ren, J.; Lau, J.; Lefler, M.; Licht, S.; The minimum electrolytic energy needed to convert carbon dioxide by electrolysis in carbonate melts. J. Phys. Chem C 2015, 119, 23342-23349.). It is unlikely that the calculated potentials are correct/precise to three digits after the decimal point, and at best 2 digits should be reported in the table.
7b) Add the following references a, b, c and l with the pertinent measured potentials in lithium carbonate,a,d,l , and the observed higher potential in both mixed sodium/lithiumd and mixed magnesium, calcium or barium /lithiumb or, mixed sodium/bariumd carbonate electrolytes, and discuss why the observed measured carbon potential in Li2CO3 is much nearer =1 V (more than 0.6 V less negative) than the calculated potential.
iii) Add two others column for (i) the listed carbonate melting point (723·°C for Li2CO3, etc), and (ii) the calculated potential not only at 500°C, but also at the carbonate melting or decomposition temperature.
l. Licht, S.; Wu, H. STEP Iron, a Chemistry of Iron Formation without CO2 Emission: Molten Carbonate Solubility and Electrochemistry of Iron Ore Impurities. J. Phys. Chem C 2011, 115, 25138-25147.

8) Regarding Figure S1, assuming that figure presented plots calculated, and not experimental, potential values explain how the carbon deposition potential CO32-/C is determined. For example, is it the lowest of the three values given in Table 1? Why it is so more negative than experimentally observed in pure Li2CO3?

9) Please remove the noise (unrelated distracting hum) from the video.

Version 1:

Reviewer comments:

Reviewer #1

(Remarks to the Author)
The authors have given a reasonable explanation to my previous concerns and made satisfactory revision in the revised manuscript. I would recommend its publication now.

Reviewer #2

(Remarks to the Author)
Reviewer 2:
The major revision to address several key points did not occur. It is recommended that the manuscript is published after revision to more fully address the following comments:
1. The main body of the text states that "most of authors agree that reaction (7) is the most probable reaction for cathodic carbon deposition in molten carbonates: CO32_ + 4e- -> C + 3O2-.
Yet the reaction the authors have used to calculate the potentials of alkali carbonate reaction, reaction (S2) is a two-carbonate reaction:
2CO32_ -> C+ 3MO2 +2CO2 +O2.
This latter reaction does not explain the low observed potentials for carbon formation. The single carbonate calculated potentials are substantially lower and are more realistic potential values for each of the alkali carbonates. For example, the simple lithium carbonate 500°C reaction potential is approximately is 0.6V less than the 1.83 V (negative sign implied) that the authors' calculate. Occam's Razor would suggest that the simpler single-carbonate, rather than two-carbonate reaction, is a better predictor of the system reaction potential. These single carbonate reaction potentials, for the carbonate, alkali reaction M2CO3 -> C +M2O + O2), have already been calculated as a function of temperature, and it is suggested that the single carbonate reaction potentials are used in the Supplementary Table 1 column 2 and 5 values, along with their appropriate references:
1a. Licht, S.; B. Wang; S. Ghosh; H. Ayub; D. Jiang; Ganley, J. New Solar Carbon Capture Process: STEP Carbon Capture.

J. Phys. Chem. Lett. 2010, 1, 2363-2368.
1b. Ren, J.; Lau, J.; Lefler, M.; Licht, S.; The minimum electrolytic energy needed to convert carbon dioxide by electrolysis in carbonate melts. J. Phys. Chem C 2015, 119, 23342-23349
Mention of the alkali earth metal carbonate section of the Supplementary Table 1, should include reference to the most recent study in beryllium carbonate, as well as the observed higher (than lithium carbonate measured potentials) potentials in magnesium and barium carbonates:
1c. Licht, G.; Hofstetter, K.; Licht, S.; Beryllium carbonate: a model compound for highest capacity carbon sequestration chemistry, RSC Advances, 14, 40133-40140 (2024); DOI: 10.1039/d4ra07753a
1d. Licht, G.; Hofstetter, K.; Xirui Wang, X.; Licht, S.; A new electrolyte for molten carbonate decarbonization," Communications Chemistry (Nature), 7, 211 (2024); https://doi.org/10.1038/s42004-024-01306-z.

2. The statement in the manuscript that "no carbon deposits have been witnessed in … pure carbonates with no lithium is misleading. This should be clarified with reference made to note the study in which extensive carbon deposits have been observed in sodium-barium carbonates, which contain no lithium, and it should be discussed and referenced:
2a. Liu, X.; Licht, G.; Wang, W.; Licht, S.; CO2 Utilization by Electrolytic Splitting to Carbon Nanotubes in Non-lithiated, Cost-Effective, Molten carbonate Electrolytes" Advanced Sustainable Systems, 2022, 2100481 (2022).

On a related note, the lower potential for sodium carbonate reduction (in the 2V domain) should be substituted in Supplementary Table 1 for the single carbonate reaction as previously discussed. This is lower than the metal deposition potential, and helps understand how the carbon deposition, rather than the competing metal deposition, occurs.

3. The discussion in the text relates to equation 8 "Additionally, it has been suggested that CO2 can also be chemically dissolved in the carbonate to form dicarbonate anions (C2O52_), which could then also take part in the reaction mechanism as an intermediate for CO2 capture: CO2 + CO32_ ⇋ C2O52_." This pathway was described in the related reference 34 as having no experimental evidence, and involves a dicarbonate product which has not been observed. It should be replaced, or at least augmented with the much more likely (again Occam's razor…) simple exogenic reaction of CO2 and oxide to make carbonate CO2 + O2_ ⇋ CO32_, which has been thermodynamically evaluated and experimentally discussed as referenced:
3a. Lau, J.; Dey, G.; Licht, S.; Thermodynamic assessment of CO2 to carbon nanofiber transformation for carbon sequestration in a combined cycle gas or a coal power plant," Energy Conservation and Management, 122, 400-410 (2016).
3b. Wang, X.; Liu, x.; Licht, G.; Wang, B.; Stuart Licht, S.: Exploration of alkali cation variation on the synthesis of carbon nanotubes by electrolysis of CO2 in molten carbonates, Journal of CO2 Utilization, 34, 303-312 (2019).
3c. Ren, J.; Licht, S.; Tracking airborne CO2 mitigation and low cost transformation into valuable carbon nanotubes," Scientific Reports, 6, 27760-1-11 (2016).

Nuances of this CO2 capture reaction have recently been observed, and relevant reactions have been discussed in the previous relevant article (and its Supplementary Materials in the section: "Oxide versus carbonate mechanism of gas evolution in separated half-cell molten carbonate electrolyses."):, which should be mentioned and referenced
3d. Licht, S.; Licht, G.; Hofstetter, K.; Large-scale electrolytic molten carbonate carbon capture and transformation to carbon nanotubes and other graphene nanocarbons" Cambridge Prisms: Carbon Technologies, 1, e6, 1-11 (2025; https://doi.org/10.1017/cat.2025.10007.

4. It is suggested that to modify the sentence in the manuscript as shown below below, with the word "allotropes" to reflect the broader study carbon nanotube allotrope study, rather than just the carbon nanotube study, and replacing the relevant reference 13:
"By CO2MSE the synthesis of varied nanocarbon products, including various types of carbon nanotube allotropes13, …"
replacement for previous reference:
13. Liu, X., Licht, G., Wang, X. & Licht, S. Controlled Growth of Unusual Nanocarbon Allotropes by Molten Electrolysis of CO2. Catalysts 2022, 12, 125 (2022).

5. The observed generation of a peroxide species during carbonate electrolysis at the cathode is a fascinating observation and should be published. However, the conclusion that it is a reactive intermediate participating in a two-electron reaction towards the formation of solid carbon remains a hypothesis. It is one of several alternative possible explanations. This question is compelling in light of the high potential noted in the manuscript for the reactive formation of peroxide from oxide (equation 12). It should discussed and reiterated in the conclusion that this is a hypothesis which needs further experimental validation, for example by suggestion further studies to examine the addition of a quenching reaction for the peroxide to check whether carbon formation than stops; by measuring the number of electrons transferred in the rate limiting step; by probing whether the peroxide species is also formed in the important higher temperature domain in which high purity graphene nano-allotropes, rather than amorphous carbon products, occurs.; or with different metal alloys, or different current densities or carbonate electrolyte compositions, some of which shave been shown to selectively efficiently produce specific carbon allotropes (and may alter local chemistry conditions), and which may be a challenge to discern through spectroscopic measurements.

Version 2:

Reviewer comments:

Reviewer #2

(Remarks to the Author)
The corrections are nearly complete. After the following suggestion (an addition to GTable S2) is considered, the manuscript is acceptable. After consideration of this correction the manuscript does not require further review by this Reviewer:

The new Supplementary Table 1 puts undo emphasis on the less likely carbon producing reactions S3 and S4 by presenting their calculated potentials at both 500°C **and** the melting point. This should be balanced in Supplementary Table 2 by adding Table 1's equivalent final column with header "Carbon deposition potential at melting point/decomposition temperature" to Table @, along with potential values appropriate for carbon produced by the S6 reaction. For example the L2CO3 value will be 1.08V. The other values (for K2CO3 and Li2CO3) can be obtained by reference S. Licht et al, "A new Solar Carbon Capture Process: Step Carbon Capture," J. Phys. Chem. Lett. 1, 2363-2368 (2010).

Open Access This Peer Review File is licensed under a Creative Commons Attribution 4.0 International License, which permits use, sharing, adaptation, distribution and reproduction in any medium or format, as long as you give appropriate credit to the original author(s) and the source, provide a link to the Creative Commons license, and indicate if changes were made.
In cases where reviewers are anonymous, credit should be given to 'Anonymous Referee' and the source.
The images or other third party material in this Peer Review File are included in the article's Creative Commons license, unless indicated otherwise in a credit line to the material. If material is not included in the article's Creative Commons license and your intended use is not permitted by statutory regulation or exceeds the permitted use, you will need to obtain permission directly from the copyright holder.
To view a copy of this license, visit https://creativecommons.org/licenses/by/4.0/

Response to the Reviewers' comments on the Manuscript:

NCOMMS-25-43100-T

"A first Operando Probing of Carbon Electroreduction from Direct Air Capture in Molten Carbonate Salt"

Dear Reviewers,

We thank you for your time and for providing insightful comments that have helped us to significantly improve the manuscript. Below, we provide a point-by-point response to the concerns raised. We have addressed all comments (in blue) and accordingly revised the manuscript (in red).

We believe the revised manuscript is now stronger and more suitable for publication in *Nature Communications*.

Reviewer #1:

The reduction mechanism of CO2/carbonate in high temperature molten carbonate is significantly important for developing a value-added CO2 capture and utilsation technology, however, the mechanism study is very challenging due to the lack of high temperature opperando methods as well as the harsh and complicated reaction environment. On the aspect, the current work is an initiative of applying spectro-electrochemistry for operando observations of CO2 reduction in molten carbonate. A carbon-adsorbed peroxide intermediate was determined under the specific experimental conditions by the authors, but the redcution mechanism may be affected by various experimental parameters such as temperature, the electrolyte composition, CO2 partial pressure, cathode materials as well as applied current density. More experiments shall be done to get an universal conclusion. Of course, this work is meaningful to the field of high temperature electrochemisty and CO2 reduction in high temperature molten slats. It could be accepted for publication after addressing following concerns.

Reply: We would like to thank Reviewer #1 for their encouraging comments. We thank them for noting the challenge in studying this topic. It is indeed true that the high-temperature molten salt environment is a complicated one to investigate, especially under electrolytic conditions. It's also a sharp observation that the reduction mechanism may be (and indeed we show some preliminary results below that it is, at least by one of them) affected by temperature, electrolyte

composition, CO_2 partial pressure, cathode materials and applied current density. For all but cathode materials, we agree that this is completely true. Different cathode materials, at least in an oxobasic melt such as the Li-Na-K eutectic surrounded by air (as in this study), seem to have little effect on the mechanism, but do affect the structure of the cathodic product, as we show here.

1. *W was used as the reference electrode in this work, however, it is not stable enough and have to be corrected with other electrodes such as Ag+/Ag, NiO/Ni.*

Reply: We thank the reviewer for this important point regarding electrochemical best practices. We agree that a tungsten quasi-reference electrode has limitations in terms of long-term potential stability compared to true reference electrodes like Ag/Ag+ or Ni/NiO. Considering the spatial constraints of the *operando* cell (the crucible has an outer diameter of 8 mm and a height of 4.5 mm) and the duration of electrolysis here (rather short at under 30 minutes), we deemed it suitable to use a convenient probe such as W as the reference electrode. While the absolute potential of the W QRE may be subject to some drift, the beginning of carbon deposition was confirmed both electrochemically via the cathodic current, visually via microscopy (as seen in Movie S1), and spectroscopically (as seen from the Raman spectra). For the potentiostatic depositions, the potential was held at −1.3 V, a value well within the carbon deposition region to ensure that the carbon deposition was proceeding regardless of minor fluctuations in the reference potential. We have performed new experiments using Ag/Ag+ and Ni/NiO, which confirm the validity of our results and added this data to the SI: To confirm the validity of the W QRE and correlate it with more stable reference systems, we performed comparative experiments using both Ni and Ag as the reference electrodes (Supplementary Figures 11 and 12).

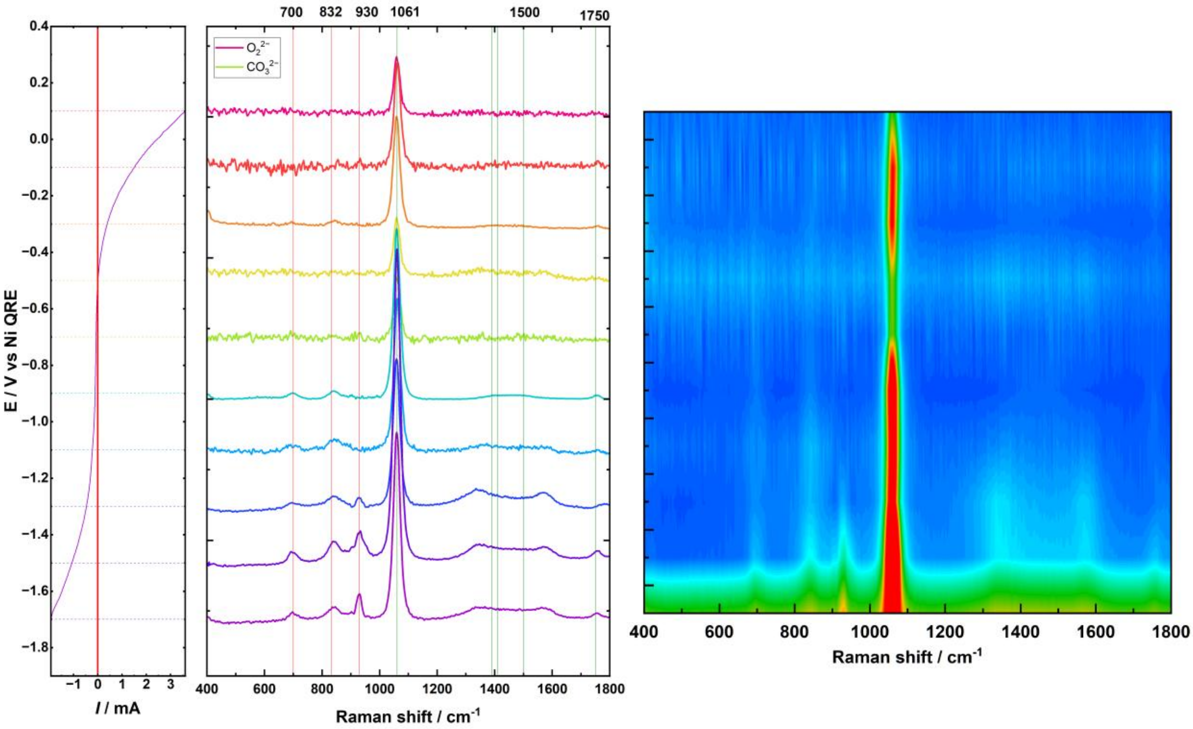

Supplementary Figure 11. Potential-dependent Raman spectra of the interface between a W electrode and the eutectic (Li,Na,K)$_2$CO$_3$ salt mixture during a linear sweep from 0.1 V to −1.7 V vs Ni QRE.

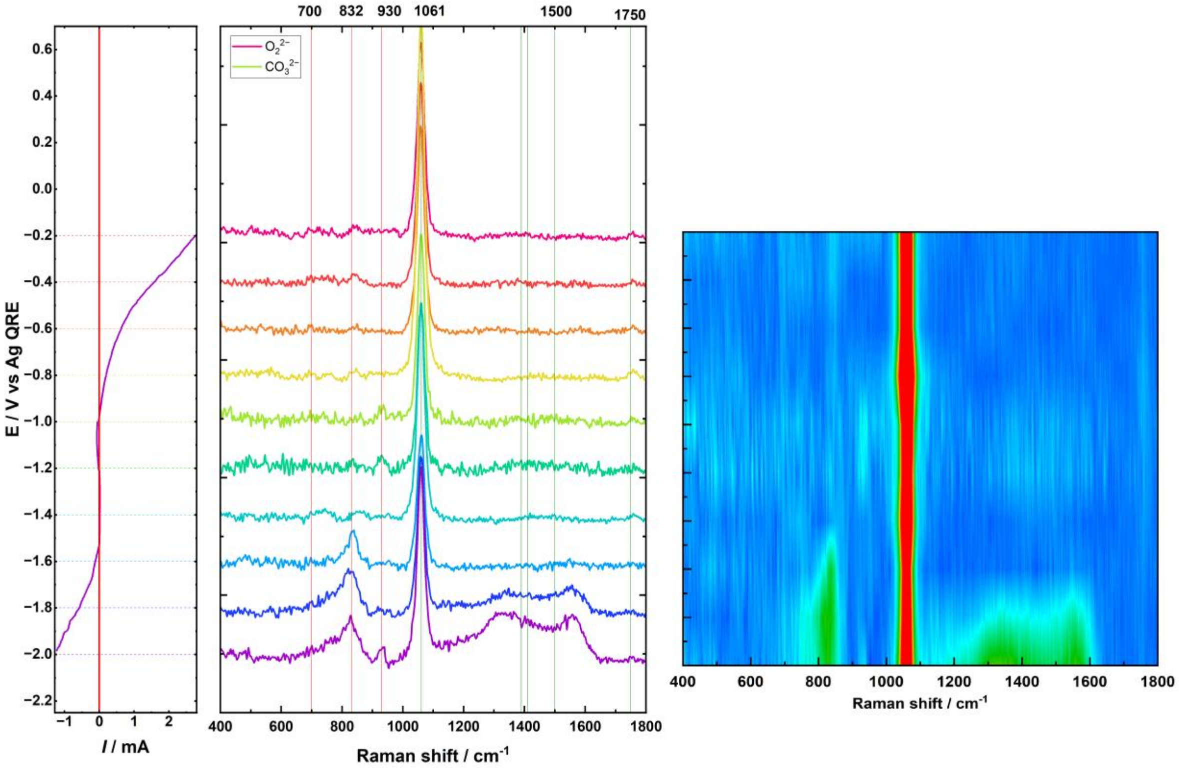

Supplementary Figure 12. Potential-dependent Raman spectra of the interface between a W electrode and the eutectic (Li,Na,K)$_2$CO$_3$ salt mixture during a linear sweep from −0.2 V to −2 V vs Ag QRE.

As can be seen, the observations regarding the peroxide intermediate stay the same. Concurrently with reduction current, a feature at centred at ~830 cm^{-1} appears, and as the electrode is further polarized and the reduction current increases, peaks according to carbon appear in the Raman spectrum. The overall potentials are shifted as expected, but the shape of the LSV, as well as the separation between the OER and carbon deposition remain the same.

2. *The reliability of the experimental data and their explanations have to be further checked. For example, the on-set potential for OER is very close according to the CVs on different electrodes in the molten carbonate. This might not be the truth, for Figure 1a, the anodic current (OER) shall be the anodic dissolution of W rather than oxygen evolution, although gas bubble was observed in the video. The gas bubbles might be the volatite WO3 and/or CO2 due to the reation W +3 CO3(2-) = WO3 + 3CO2.*

Reply: We appreciate Reviewer#1's insightful comment on this topic. Due to the proximity of anodic dissolution of W to the OER, this could seem like a plausible explanation. Volatile WO_3 is not a credible source of bubbles at 500 °C. At our temperature, WO_3 has a negligible vapor pressure, and in a strongly basic carbonate (direct air capture conditions) its activity is further suppressed by conversion to surface tungstate. Sustained, copious gas bubbles are therefore O_2, not WO_3 vapor. We carefully considered the possibility of tungsten oxidation, performed elemental analysis of the salt after anodic testing of a W electrode and compared it to pure salt and salt with a known content of W, with less than 2.5% of the expected W seen in the salt. This points to oxygen evolution on a passivated W oxide surface with low net dissolution of tungsten under our conditions (500 °C, Li-Na-K carbonate, static air, W quasi-reference). The cell was run at a potential of 0.4 V vs W QRE for 1440 s and the salt analysed after that using total reflection X-ray fluorescence as described in section 8 of the SI (added):

To confirm that the anodic reaction seen in the CV on Figure 1a is oxygen evolution and not the anodic dissolution of tungsten, a potential of 0.4 V vs W QRE was applied to the working electrode for 1440 s (Supplementary Figure 13).

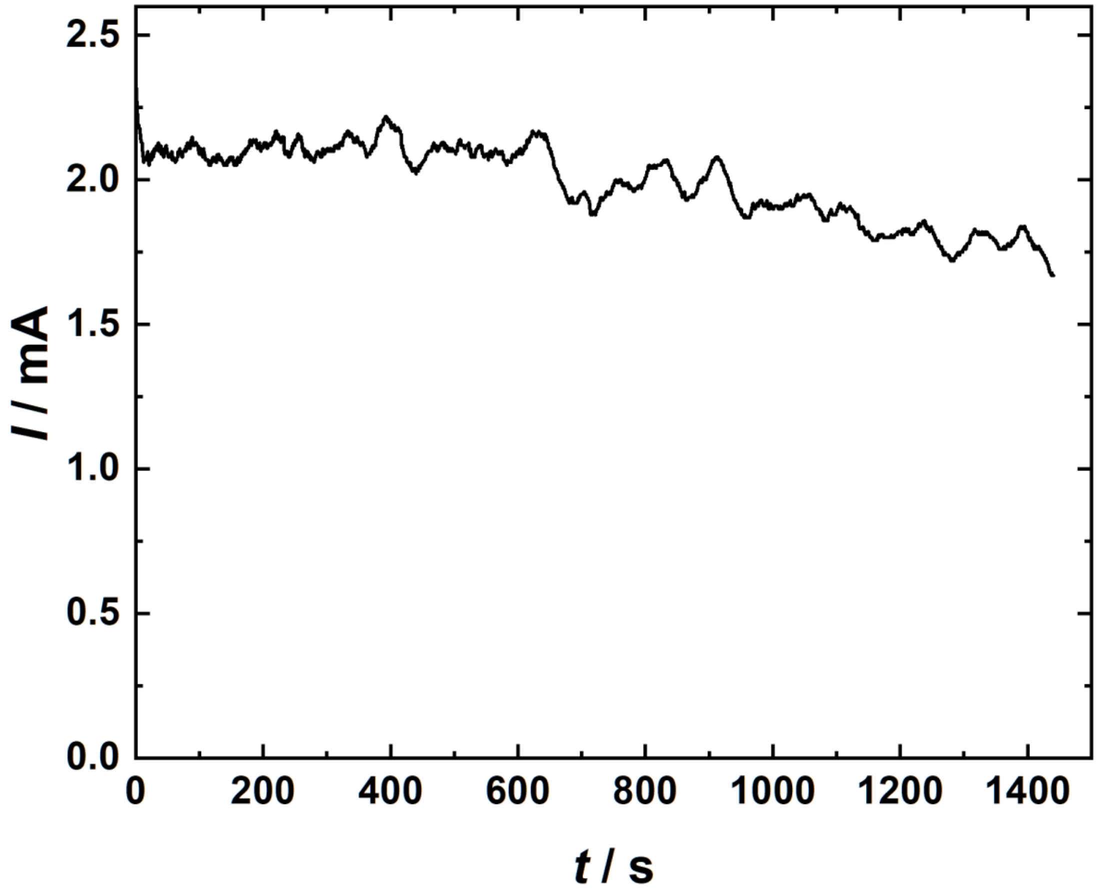

Supplementary Figure 13. Current response over 1440 s of a CO_2 electrolysis reactor with an applied potential on the working electrode of 0.4 V at 500 ºC. The working, counter and reference electrodes were W.

After this the reactor was cooled down and the electrodes removed from it, the salt was sampled by scraping it from the areas where the electrodes were during the measurement. 10 mg of this salt was then weighed into a vial and digested using a mixture of 450 µl of concentrated HCl (>37%, p.a., Fluka) and 50 µl of a 100 ppm Ga internal standard in concentrated HNO_3. After full digestion of the salt, the samples were analysed using total reflection X-ray fluorescence. For comparison, a fresh Li-Na-K carbonate mixture and a Li-Na-K carbonate mixture to which 1 wt.% of WO_3 was added (to get 1 wt.% of W in the salt) were also analysed. The results are given in Supplementary Table 4, with the digestion efficiency of W confirmed as 93.27% via digestion of the 1 wt.% W salt.

Supplementary Table 4. W content as-determined by TXRF in pristine Li-Na-K carbonate, Li-Na-K carbonate with 1.27 wt.% of WO_3 was added (which accords to 1 wt.% W).

Sample	W (ppm)
**Pristine (Li-Na-K)$_2$CO$_3$**	0
**(Li-Na-K)$_2$CO$_3$ + 1.27 wt.% WO$_3$**	9327
**(Li-Na-K)$_2$CO$_3$ after 1440 s electrolysis**	354

As can be seen, while the salt after electrolysis contains some W, the content is very low. In an oxo-basic melt at a temperature of 500 ºC, formed WO_3 would be expected to form soluble Li_2WO_4 via the reaction $WO_3 + Li_2O = Li_2WO_4$. The mass of the salt prior to electrolysis was 62.3 mg. Using the total charge passed during the electrolysis (Supplementary Figure 14, 2.86 C total), and considering a 6-electron oxidation of W, we find that a mass of 0.908 mg of W would be expected to be oxidized off the electrode (considering a Faradaic efficiency of 100%), which would correspond to 14686 ppm of W in the electrolyte. This is considerably more than the 354 ppm detected, which would accord to 2.41% of the current, proving that the reaction cannot be tungsten oxidation.

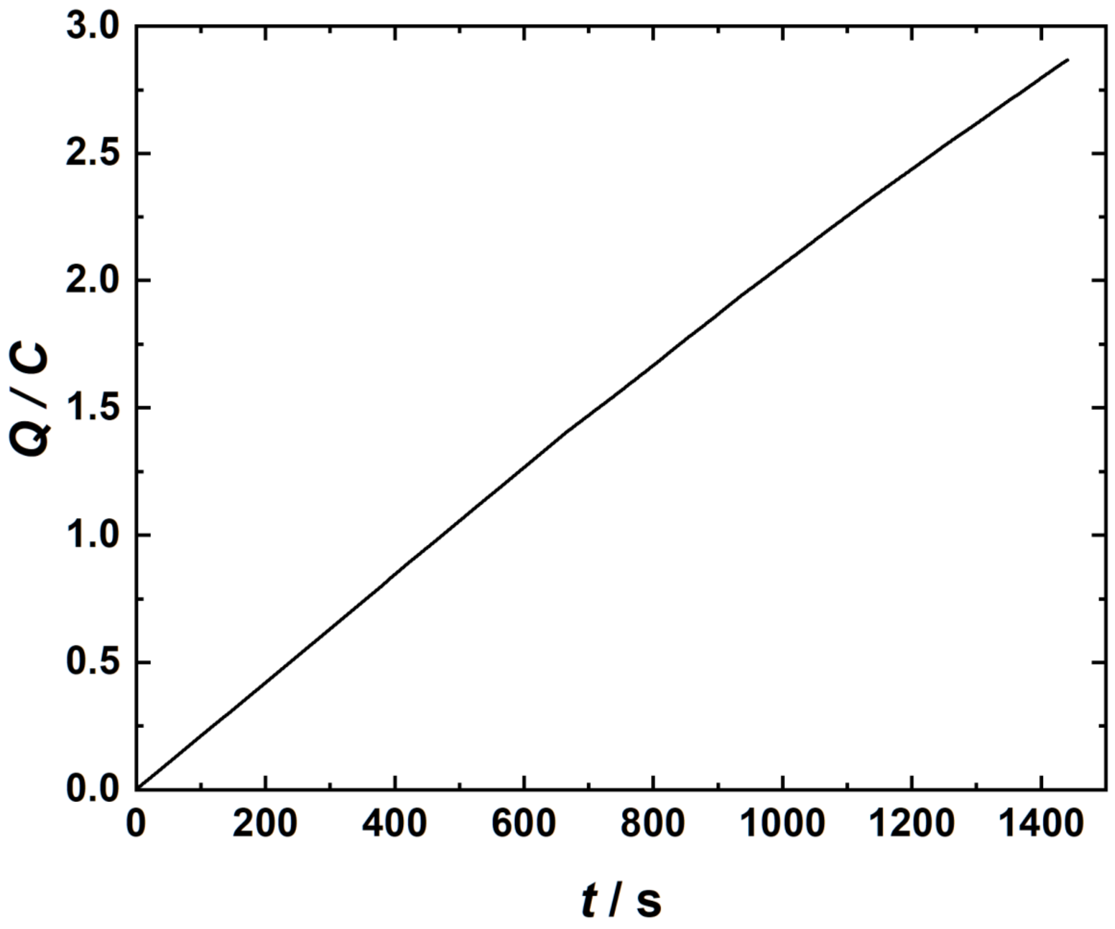

Supplementary Figure 14. Charge passed during CO_2 electrolysis calculated from the data on Supplementary Figure 13.

Thus, the anodic dissolution of W remains unlikely. A more likely explanation is the partial oxidation/dissolution of W as the salt becomes molten (explaining the low W content in the melt), and the formation of a thin, compact WO_3 film (likely the nature of the Ox_1 peak on the W cyclic voltammogram on Figure 1a), that can support OER while inhibiting further W dissolution under anodic polarization. This is consistent with both the TXRF and polarization curves.

To conclude, if substantial W dissolution had occurred during the anodic sweeps/holds, significant W would be expected in the electrolyte. Its absence rules out anodic dissolution as the dominant anodic process.

3. *More RS tests shall be conducted under different CO2 partial pressure and polarization conditions to confirm the proposed mechanism.*

Reply: We agree with the reviewer that the reaction mechanism is likely dependent on a variety of experimental parameters, including CO_2 partial pressure and applied potential. This study was designed as a foundational investigation, providing the *first* operando spectroscopic evidence for the reaction pathway under conditions relevant to **direct air capture**, which inherently involves low CO_2 partial pressures. We believe that this initial work provides a crucial benchmark and opens the door for subsequent investigations, which it is important to publish it now. A extensive parametric study of the mechanism at differing CO_2 partial pressures and potentials is, in our view, beyond the scope of one manuscript. We believe the best way to further the field is to get the information and the method out there and inspire other researchers to follow suit.

We have added a statement to the conclusion acknowledging that a systematic study of the effects of CO_2 partial pressure, temperature, and electrolyte composition is a vital and exciting direction for future research that will build upon the findings presented here: "This opens new dimensions into understanding the carbon reduction process from direct air capture and industrial waste streams, and enables design of carbon materials from CO_2 with control of structure and real-time process monitoring, but a systematic study at other CO_2 partial pressures, temperatures and electrolyte compositions is essential to widen these prospects."

4. *Was surface enhanced effect observed on Au electrode at the high temperature?*

Reply: This is an interesting question. While gold is well-known for its ability to produce a Surface-Enhanced Raman Scattering (SERS) effect, our experiments were not specifically designed to induce or rely on it as it would be rather difficult experimentally, considering the molten salt environment. No electrochemical roughening protocols were applied to the electrode surface prior to the measurements. The signal-to-noise ratio on the Au electrode was comparable to that on other electrodes and was sufficient for our analysis without needing to use SERS. While it is possible that some minor surface restructuring during electrolysis could have led to a slight enhancement, we have no direct evidence of this. We have added a sentence to the manuscript to clarify that SERS was not an intended or necessary component of our measurements:

"No surface enhancement effects were used for collecting the Raman signals."

Reviewer #2:

This is an important study presenting the first real-time in-situ spectroscopic analysis of a useful decarbonization process to mitigate the ongoing buildup of the greenhouse gas CO2. There are several inconsistencies and as delineated below major revision is needed. Subsequent to the appropriate major revision, and another review, the manuscript is recommended for publication.

Reply: We are grateful to the reviewer for appreciating the importance of our work, and we thank the reviewer for the recommendation to publish our manuscript after according revisions.

1. *The implication in the title that operando molten carbonate measurements have not been previously made is incorrect, and it should be corrected in the title,*
1a) Title: Correct the title by addition of the qualifying underlined term:
1a) "A first Spectroelectrochemical Operando Probing of Carbon Reduction from Direct Air Capture in Molten Carbonate, "
1b) On a related note, the main text should be corrected to include references to prior operando molten carbonate measurements. That is, change the following phrase to include previous operando examples by adding the underlined text and references,
Change from: ",… while progress in looking at the mechanistic details of this process remains limited due to a lack of tools for operando investigation of the process."
to: ". Operando measurements of cyclic voltammetry, current steps, CO2 and O2 concentrations have been made (in real time) during carbon reduction in molten carbonate.a-c However, progress on the mechanistic details of this process remains limited due to a lack of tools for spectroscopic operando investigation of the process.
a. Licht, S.; B. Wang; S. Ghosh; H. Ayub; D. Jiang; Ganley, J. New Solar Carbon Capture Process: STEP Carbon Capture. J. Phys. Chem. Lett. 2010, 1, 2363-2368.
b. Ren, J.; Johnson, M.; Singhal, R.; Licht, S. Transformation of the Greenhouse Gas CO_2 by Molten Electrolysis into a Wide Controlled Selection of Carbon Nanotubes. J. CO_2 Util. 2017, 18, 335-344.
c. Ren Wang, X.; Liu, X.; Licht, G.; Wang, B.; Licht, S. Exploration of Alkali Cation Variation on the Synthesis of Carbon Nanotubes by Electrolysis of CO_2 in Molten Carbonates. J. CO_2 Util. 2019, 18, 303-312.

Reply: We thank the reviewer for this important point and agree that the title and introduction should more clearly reflect the distinction between different types of operando measurements. To address this, we have revised to now read "*Operando* spectroelectrochemical identification of peroxide intermediate in molten carbonate CO_2-to-carbon electroreduction" as the title suggested by the Reviewer would be over the 15-word limit of Nature Communications. This change clarifies that our novelty lies in the *spectroelectrochemical* operando investigation, rather than in electrochemical operando studies more broadly. At the same time, we respectfully note that **cyclic voltammetry or current step measurements by themselves do not constitute operando probing in the strictest definition**, since they record the electrochemical response but not the real-time identity of products, intermediates, or changes in electrode surface characteristics. By contrast, operando gas analysis qualifies as operando, as it provides direct, time-resolved information on gaseous products. Our work extends this operando capability by introducing *spectroscopic identification* of intermediates and products (via Raman), which enables mechanistic probing not possible from electrochemistry alone.

We have revised the Introduction (Page 2) to explicitly acknowledge this previous operando study in molten carbonate electrolysis: "Previous operando studies have employed electrochemistry in tandem with gas analysis in real time to monitor CO_2 reduction in molten carbonates[23]. While these electrochemical and gas-evolution measurements provide important operando insights, the mechanistic details of intermediate species have remained limited due to the lack of spectroscopic operando investigation."

The last part of the sentence the Reviewer suggested to revise has thus been removed as this information is present in the new sentence.

As suggested, we now cite (Wang, X., Liu, X., Licht, G., Wang, B. & Licht, S. Exploration of alkali cation variation on the synthesis of carbon nanotubes by electrolysis of CO_2 in molten carbonates. *Journal of CO2 Utilization* **34**, 303–312 (2019)), which demonstrated real-time gas concentration analysis during carbon reduction. This is indeed an operando technique, in the sense that both the electrochemical and gas evolution processes were monitored during operation.

We believe these clarifications acknowledge prior operando work while making the scope of our contribution precise.

2. *It would be useful for the researchers to strengthen their results that peroxide, rather than oxide or superoxide, is a reaction intermediate to carbon reduction. This new study does an excellent job of correlating the presence of peroxide with carbon growth. However, is it*

alternatively possible that this result might be interpreted that oxide or superoxide could be a principal species in the carbon reaction?

This is because in molten salts there is a dynamic equilibrium between oxides, peroxide and superoxides:

2O2- + O2 <-> O22-; O22- + O2 <-> 2O2-

In molten carbonates, the dominate species of the three has been well studied, varies, and is a strong function of the alkali or alkali earth cation and temperature of the carbonate melt. Table 2 of the supplementary information apparently notes that peroxide, but not oxide or superoxide, can be identified by this study's Raman spectroscopy in carbonate melts. Hence, while peroxide was measured, it might only be present as a consequence of the dynamic equilibria, and is it possible instead that either oxide or superoxide is the principal oxygen intermediate, growing by equilibrium with peroxide and directly participating in the carbon reduction mechanism? This question is compelling in light of the high potential noted in the manuscript that is necessary to complete the reaction of peroxide to oxide (equation 6).

Reply: We thank the reviewer for raising this important mechanistic point. We fully agree that O^{2-}, O_2^{-}, and O_2^{2-} exist in a dynamic equilibrium in molten carbonates, and that in principle all could play roles in carbon electroreduction. Our experimental results, however, allow us to rule out oxide and superoxide as intermediates under our conditions:

1) **Superoxide (O_2^{-}):** Raman-active features of O_2^{-} are expected in the 1047–1160 cm^{-1} region (Fischer, D., Zagorac, D. & Schön, J. C. The presence of superoxide ions and related dioxygen species in zinc oxide—A structural characterization by in situ Raman spectroscopy. Journal of Raman Spectroscopy 53, 2137–2146 (2022); Hester, R. E. & Nour, E. M. Resonance Raman studies of transition metal peroxo complexes: 4-The potassium μ-Peroxo-bis[pentacyanocobaltate(III)], $K_6[(CN)_5CoO_2Co(CN)_5]\cdot H_2O$ and μ-superoxo-bis-[pentacyanocobaltate(III)], $K_5[(CN)_5CoO_2Co(CN)_5]\cdot H_2O$ complexes. *Journal of Raman Spectroscopy* **11**, 43–48 (1981)), but no such bands were observed in any operando spectrum, across all electrodes and potentials. This absence strongly indicates that superoxide does not accumulate and is not part of the deposition pathway in our system.
2) **Oxide (O^{2-}):** Oxide ions are always present in the molten carbonate even before electrolysis begins. They are present as a background species regardless of whether

carbon is forming (albeit at higher concentrations during deposition as they are the second product) and cannot explain the observed correlation between Raman features and deposition. Oxide ions themselves have no Raman signature.

3) **Peroxide (O_2^{2-}):** The only new oxygen-related Raman band to emerge during electrolysis was the ~832 cm^{-1} signal assignable to peroxide. This feature grows reproducibly in step with carbon deposition at the cathode, on all tested electrode materials, which means that the dynamic equilibrium near the cathode is shifted towards peroxide when carbon is being deposited. The most consistent explanation for this is that it takes part in the reaction mechanism.

If peroxide were merely a passive product of the **O^{2-}/O_2^{2-}/O_2^{-}** equilibrium, it would not be expected to appear selectively and reproducibly at the electrode interface exactly when carbon growth occurs. The temporal and spatial correlation strongly supports peroxide as the mechanistically relevant intermediate.

In the revised Discussion (Page 12), we make it explicit that peroxide is the only oxygen species observed operando, and that both superoxide and oxide can be excluded as intermediates in the deposition pathway: "Among the oxygen species possible in molten carbonates, peroxide (O_2^{2-}) is the only intermediate evidenced by operando Raman spectroscopy. Bands characteristic of superoxide (1047–1160 cm^{-1}) were absent under all conditions, ruling out its involvement. While the concentration of oxide ions (O^{2-}), which have no Raman signal, is expected to increase during carbon deposition as they are the second final product, this does not explain the sudden increase in peroxide content. In contrast, the peroxide band at ~830 cm^{-1} emerges reproducibly and increases concurrently with carbon deposition, providing direct evidence that O_2^{2-} is the mechanistic intermediate driving the CO_2-to-carbon pathway under our conditions."

3. *The phase: "At temperatures above 500 °C in oxygen-rich environments (such as air), amorphous carbon can start to be oxidized in a mixture of carbonates and solid carbon33, but" is misleading as causing a temperature measurement limitation to the present study. The carbon product grown at the cathode is protected against oxidation by the applied electrolysis cathodic bias, and hence not subject to oxidation by the hot O2 generated by the anode. This is evidenced by the high coulombic efficiency (approaching 100%) demonstrated at 770°C in the study:*

Licht G.; Peltier, E.; Gee, S.; Licht, S. Eliminating active CO2 concentration in Carbon Capture and Storage (CCUS): Molten carbonate decarbonization through an insulation/diffusion membrane, Decarbon 2025, 7, 1000944.

Reply: We thank the reviewer for directing our attention to this issue. We agree that during electrolysis the cathodic bias protects the freshly deposited carbon from oxidation, and we have revised the text to avoid implying otherwise. Our original intention was to highlight a practical constraint relevant to product recovery: in an industrial or laboratory harvest step the cathode (and adhered carbon/carbonate) must be removed or otherwise exposed out of bias and often to oxidizing atmospheres at high temperature. Under those conditions (hot carbon in contact with carbonate and O_2) oxidation of the carbon products, especially in the case where they are rather amorphous, such as here, can occur (see Figure S4 in reference 37 of the main text, showing the oxidation of carbon in a C/Li_2CO_3 mixture). This consideration was what we meant to highlight as one of the motivations was of our choice of 500 °C for operando work, not a claim that carbon oxidizes during biased deposition. To clarify this, the sentence has been modified in the main text to now read: Although the deposition process itself can approach coulombic efficiencies up to 100%[37], during product removal at temperatures above 500 °C in oxygen-rich environments (such as air), amorphous carbon can start to be oxidized in a mixture of carbonates and solid carbon[38]. The working temperature of the salt must be somewhat above the melting point of the salt to induce faster mass transfer, which is why the working temperature was set to be as high as possible without potentially oxidizing the deposited carbon.

4. *Continuation of that manuscript phrase "33, but the temperature must be somewhat above the melting point of the salt to induce faster mass transfer, which is why the working temperature was set to be as high as possible without potentially oxidizing the deposited carbon." As discussed above, the protected carbon product molten carbonate working temperature is not limited to 500°C; this leads to another question, why was the spectroscopic analysis conducted only at 500°C? The more significant graphene nano-carbon allotrope CO2 reduction products are formed at a working temperature above 650°C (Liu, X.; Licht, G.; Wang, X.; Licht, S. Controlled Growth of Unusual Nanocarbon Allotropes by Molten Electrolysis of CO2. Catalysts 2022, 12, 125) and perhaps are dominated by a different carbon reduction mechanism (although this 500°C study is a very*

useful start). On that note, it would also be useful to include SEM and TEM in the paper or SI characterizing the carbon product grown in the study.

Reply: We thank the reviewer for this valuable observation. We agree that higher temperatures (≥650 °C) are known to favour the formation of more crystalline nanocarbon allotropes, as shown by Liu *et al.* However, lower temperature syntheses can also form valuable products in the form of catalysts, catalyst precursors, and composite electrodes as we have demonstrated previously (see refs. 15-17 in the manuscript). The materials synthesised here have surface morphologies similar to our previously studied carbons as seen from the SEM and TEM images added to the SI, as requested, along with discussion:
The surface morphology of the carbon deposits from different cathodes can be seen in Supplementary Figures 6-9. On W cathode, the deposit was a mixture of carbon nano-onions and amorphous carbon (Supplementary Figure 6). In the case of Ni cathode (Supplementary Figure 7), carbon nanotubes and platelet-like carbon appeared in the mixture, whereas for Inconel 600 cathode (Supplementary Figure 8) carbon platelets were the primary product. Finally, for the Au cathode (Supplementary Figure 9), purely amorphous carbon morphology was seen in the deposit via SEM. TEM (Supplementary Figure 10) reveals the structure of the material deposited on the W electrode – a mixture of amorphous areas and graphitic structures (Supplementary Figure 10c-d), with the graphitic edges of the nano-onions especially prominent (Supplementary Figure 10a-b).

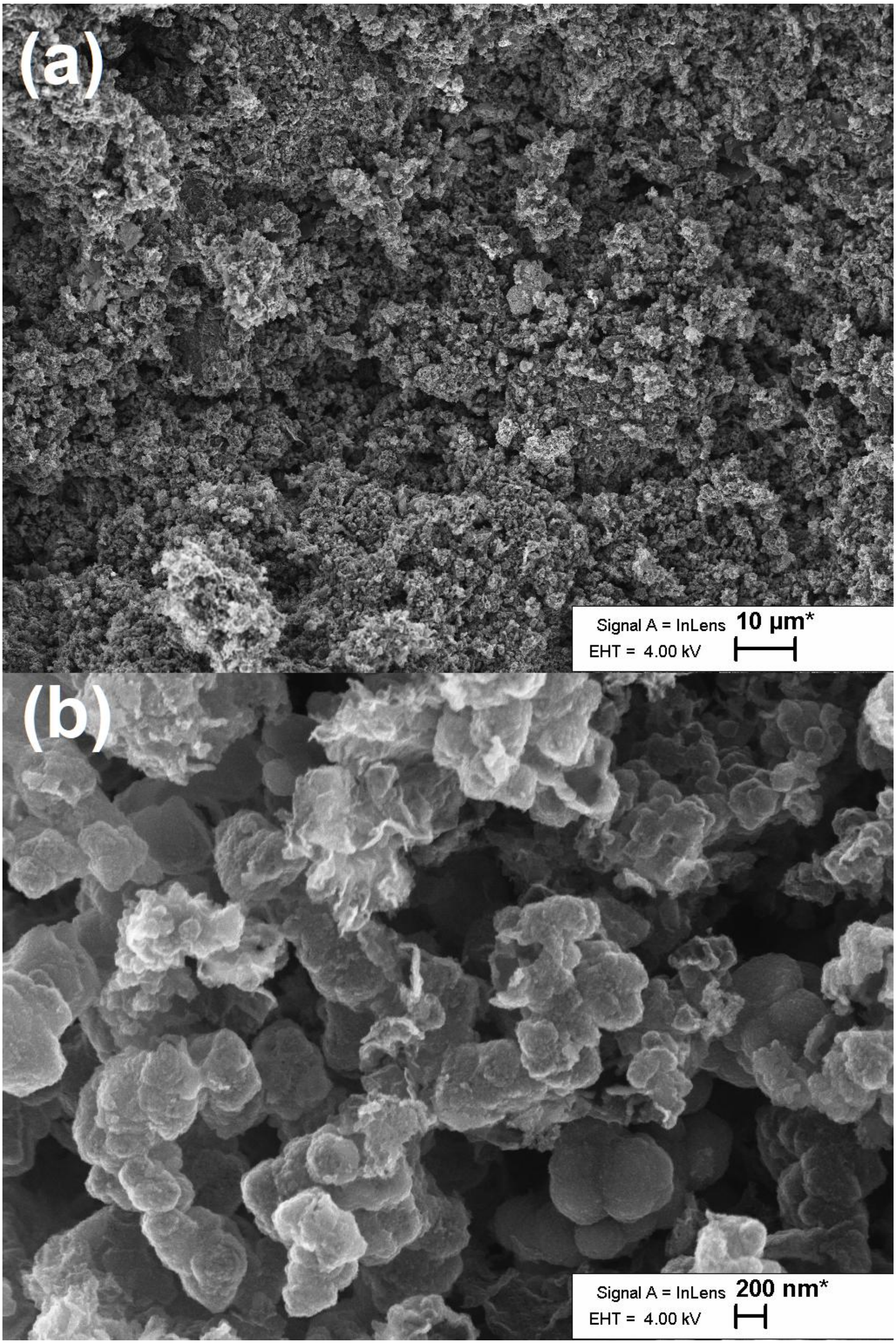

Supplementary Figure 6. Low- (a) and high-magnification (b) scanning electron microscopy images showing the surface morphology of the material deposited on a W electrode.

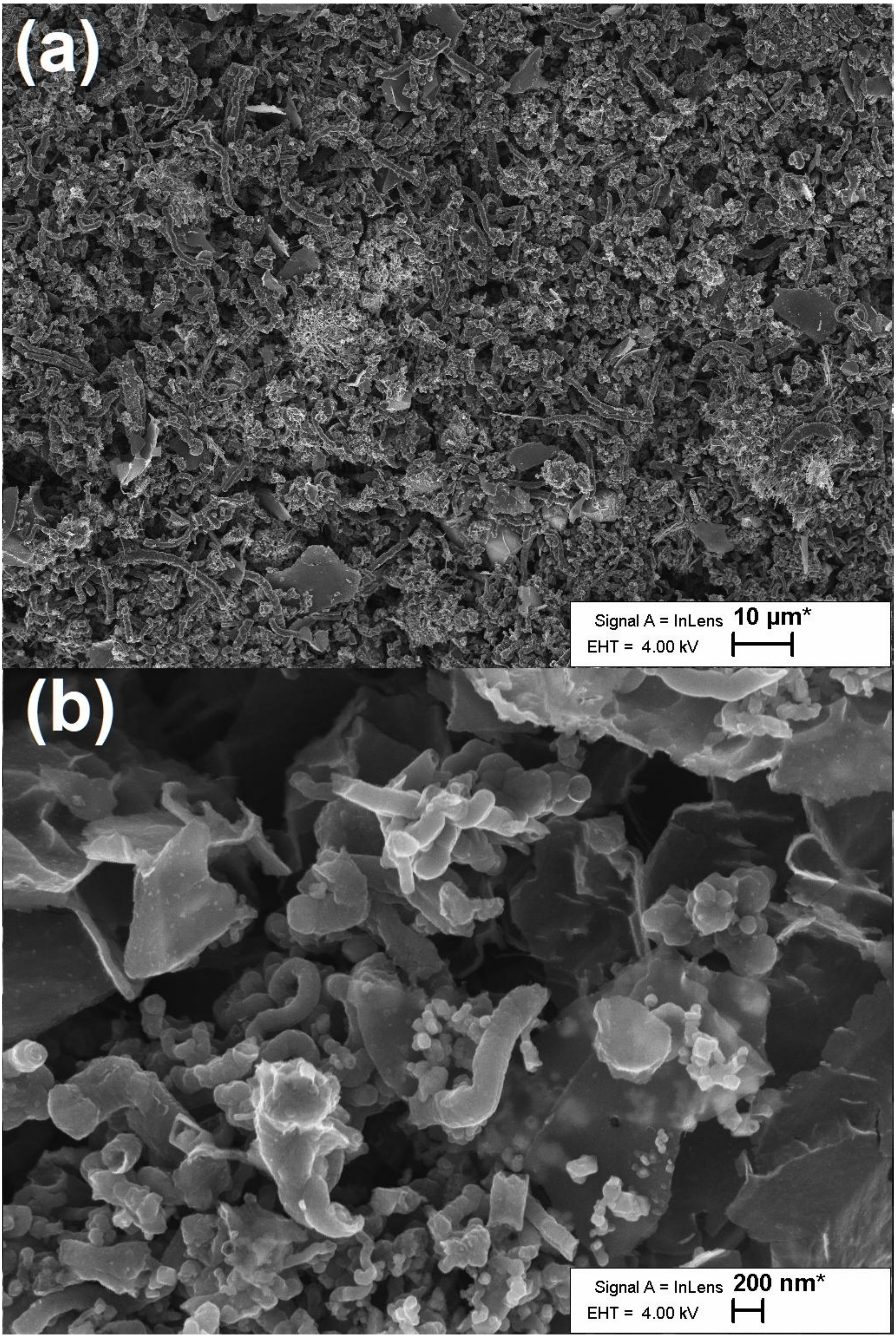

Supplementary Figure 7. Low- (a) and high-magnification (b) scanning electron microscopy images showing the surface morphology of the material deposited on a Ni electrode.

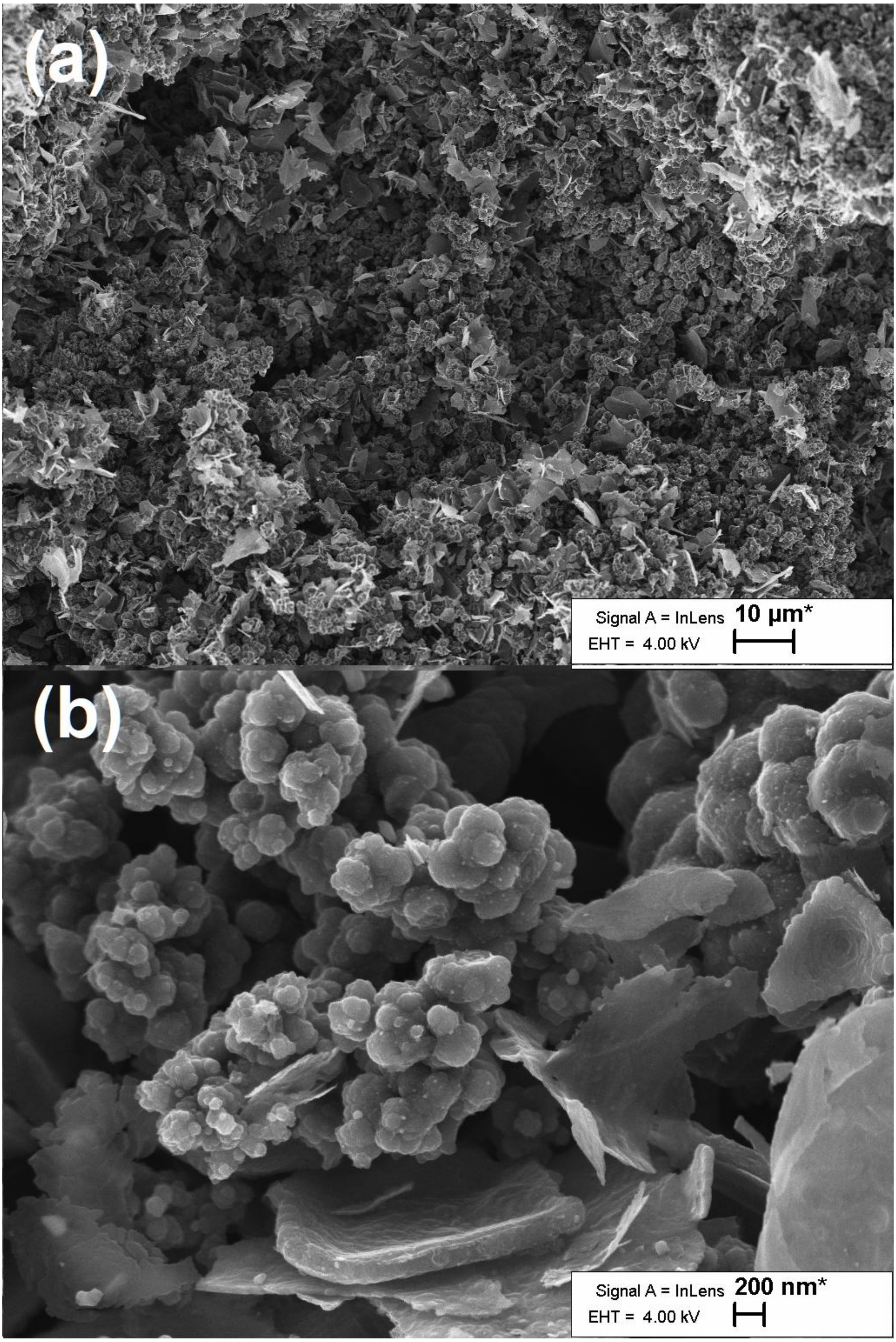

Supplementary Figure 8. Low- (a) and high-magnification (b) scanning electron microscopy images showing the surface morphology of the material deposited on an Inconel 600 electrode.

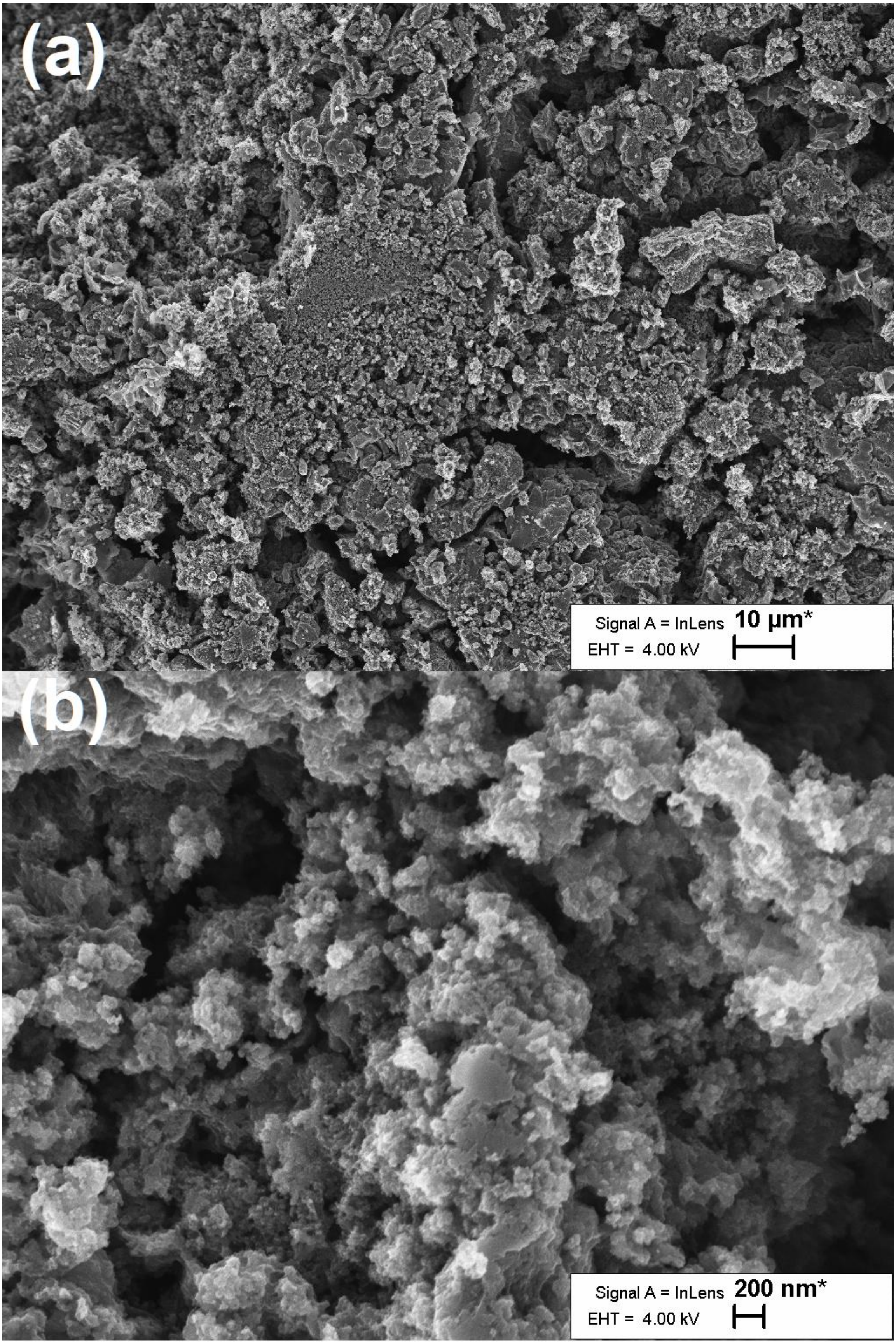

Supplementary Figure 9. Low- (a) and high-magnification (b) scanning electron microscopy images showing the surface morphology of the material deposited on an Au electrode.

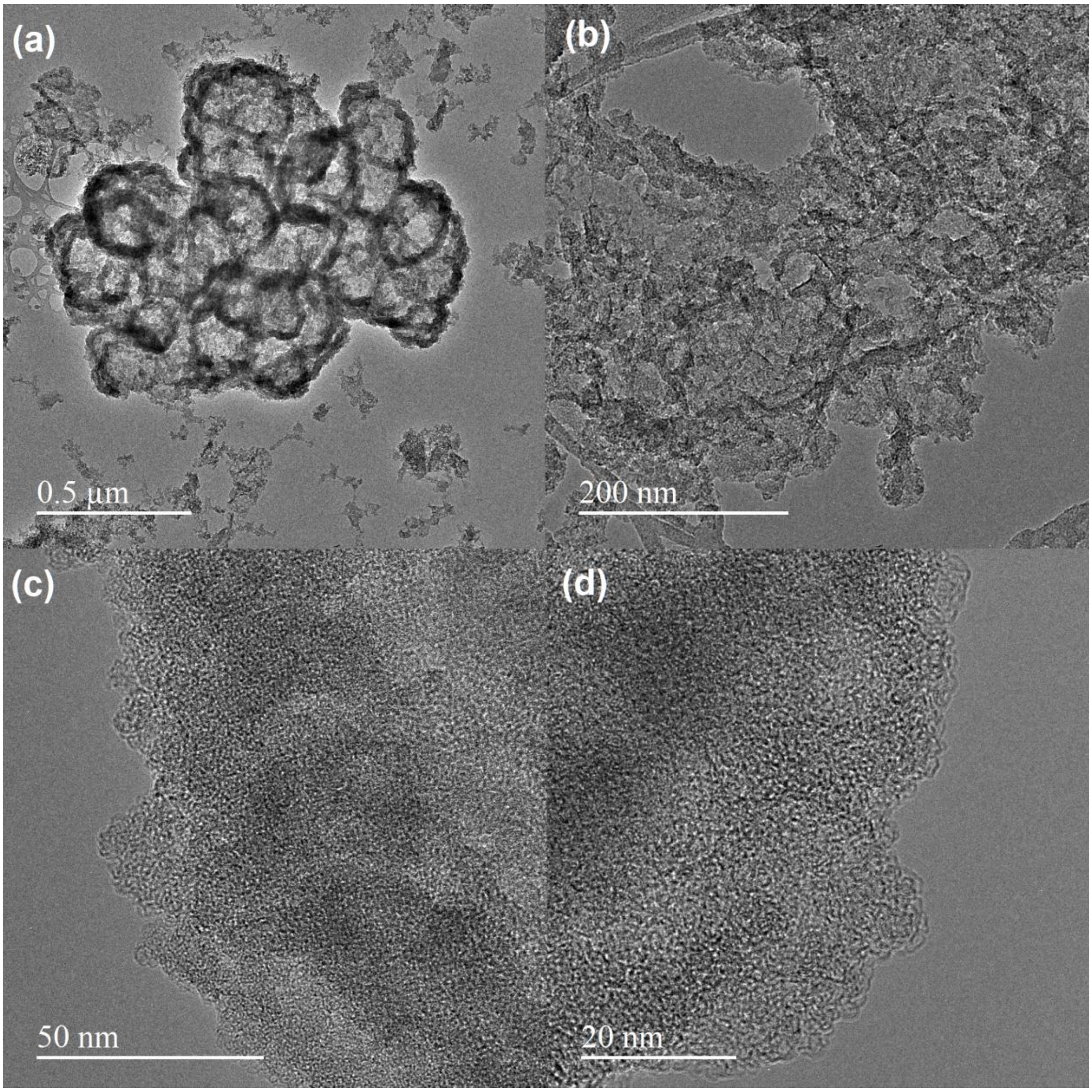

Supplementary Figure 10. Transmission electron microscopy images showing the structure of the material deposited on a W electrode.

In addition to the practical consideration on product oxidation described above, lower temperature syntheses are more efficient in terms of lower energy requirements for heating. For spectroelectrochemical studies specifically, higher temperatures also mean more intense blackbody radiation, which lowers the signal to noise ratio for Raman spectroscopy.

5. *a) Abstract: While, a great deal needs to be learned or reaffirmed about the underlying reaction of molten carbonate CO2 reduction, much is already known, and the qualitative terms 'ill' and 'suffer' in the abstract diminish objectivity (they are "ill"-advised). "ill" and 'suffer' qualitatively suggest that the foundation of understanding of the mechanism of*

molten carbonate reduction is less advanced than it actually is. Please revise that sentence in the abstract.

Reply: We thank the reviewer for this suggestion to improve the objectivity and context of our manuscript. We agree that the original wording in the abstract was imprecise. We revised the abstract to remove subjective phrasing and the sentence has been edited to read more objectively: „Electrolysis of CO_2 in molten salts promises efficient carbon capture, but the underlying reaction mechanisms remain incompletely understood, as research thus far has been limited by a lack of tools for *operando* investigations."

b) On a related note, in the text of the main manuscript consider adding the following few sentences, to present a foundation of understanding of the underlying reaction mechanisms of the electrolysis of CO2 in molten salts. Add: "The individual reactions occurring at the anode and cathode are still being explored.d However, a current understanding of the reduction mechanism of CO2 to carbon in molten carbonates includes (i) the rapid, exothermic reaction of gas phase CO2 with oxide in the electrolyte to form carbonate, (iia) the simultaneous reduction of cathodic carbonate to form carbon and (iib) anodic oxidation of oxide to form oxygen, for a net reaction of CO2 -> C + O2.e Transition metal nucleation at the cathode drives the formation of specific graphene nano-carbon morphologies, and root or tip growth of the carbon at the cathode have been demonstrated.f High iron or nickel drives carbide formation and magnetic CNT formation,g while high current density, and additives causing heptagonal, rather hexagonal carbon building blocks generate torsional effects causing the formation of helical graphene nano-allotropes.h Inhibition of transition metal nucleation suppresses 1D symmetry CNT growth and facilitates 0D symmetry carbon nano-onion,i 2D graphene and carbon nano-platelet,j and 3D carbon nano-scaffold growth.k

d. Ren, J.; Lau, J.; Lefler, M.; Licht, S.; The minimum electrolytic energy needed to convert carbon dioxide by electrolysis in carbonate melts. J. Phys. Chem C 2015, 119, 23342-23349

e. Ren, J.; Yu, A.; Peng,P.; Lefler, M.; Li, F.-F.; S. Licht, Recent advances in solar thermal electrochemical process (STEP) for carbon neutral products and high value nanocarbons. Acc. Chem. Res. 2019, 52, 3177–3187.

f. Johnson, M.; Ren, J.; Lefler, M.; Licht, G.; Vicini, J.; Liu, X.; Licht, S. Carbon Nanotube Wools Made Directly from CO_2 by Molten Electrolysis: Value-Driven Pathways to Carbon Dioxide Greenhouse Gas Mitigation. Mater. Today Energy 2017, 5; 230-236.

g. Wang, X.; Sharif, F.; Liu, X.; Licht, G.; Lefler, M.; Licht, S. Magnetic carbon nanotubes: carbide nucleated electrochemical growth of ferromagnetic CNTs, J. CO_2 Util. 2020, 40, 101218.

h. Liu, X.; Ren, J.; Wang, X.,; Licht, S, Carbon Nano Onions Made Directly from CO_2 by Molten Electrolysis for Greenhouse Gas Mitigation. Adv. Sustain. Syst. 2019, 3, 1900056

i. Liu, X.; Licht, G.; Wang, X.; Licht, S. (2021). The green synthesis of exceptional braided, helical carbon nanotubes and Nanospiral Platelets Made Directly from CO_2. Mater. Today Chem. 2021, 22, 100529.

j. Liu, X.; Wang, X.; Licht, G.; Licht, S. Transformation of the Greenhouse Gas Carbon Dioxide to Graphene. J. CO_2 Util. 2020, 236, 288-294.

k. Wang, X.; Licht, G.; Liu, X.; Licht, S. One Pot Facile Transformation of CO_2 to an Unusual 3 D Nano-scaffold Morphology of Carbon. Sci. Rep. 2020, 10, 21518.

To emphasize the fundamental and climate change importance of the study expand the following text sentence by considering adding the underlined terms to demonstrate the varied reported nanocarbon allotropes products that have been synthesized:

"By CO2MSE the synthesis of varied nanocarbon products, including thin, wool, magnetic, helical, nano-bamboo, nano-pearl, branched and macro-assemblies of carbon nanotubes13, nano-platelets and graphene14, doped carbons,15,16,c , carbon nano-onions, carbon nano-scaffolds and even oxide-carbon composites17 have been demonstrated in separate studies by the Licht group. It is also useful to reference that prior to references 15 and 16, doped CNTs were reported at: Ren, J.; Johnson, M.; Lefler, M.; Licht, G; Vicini, J.; Licht, S. Data on SEM, TEM, and Raman Spectra of Doped, and 35,36 Carbon Nanotubes Made Directly from CO_2 by Molten Electrolysis. Data in Brief 2017, 14, 592-606.

Reply: Thank you for suggesting a more detailed overview of the current understanding of the mechanism and products of the CO2MSE. We agree that this will improve the manuscript and have incorporated the substance of the reviewer's suggested paragraph and the relevant citations into the introduction. To maintain a focus on the most relevant mechanisms leading into our work, we have slightly condensed the description of the various nanocarbon morphologies.

The following sentences have been added or edited: "The current understanding of the reduction mechanism of CO_2 to carbon in molten carbonates includes the rapid, exothermic reaction of gas phase CO_2 with oxide in the electrolyte to form carbonate, with a simultaneous

reduction of cathodic carbonate to form carbon and anodic oxidation of oxide to form oxygen. By CO2MSE the synthesis of varied nanocarbon products, including various types of carbon nanotubes[13], nano-platelets and graphene[14], doped carbons[15,16] and even oxide-carbon composites[17] has been demonstrated. Transition metal nucleation at the cathode can drive the formation of specific graphene nano-carbon morphologies, with both root and tip growth mechanisms having been proposed. High concentrations of iron or nickel can drive carbide formation, leading to magnetic CNTs[18]. Conversely, inhibiting transition metal nucleation suppresses 1D CNT growth and facilitates the formation of 0D carbon nano-onions[19], 2D graphene and carbon nano-platelets[14], and 3D carbon nano-scaffolds[20].

6. *Revisions needed related to Table S1:*
   7a) Please add rows for the deposition potentials of alkali earth metals from carbonates (Be, Mg, Ca, Sr, Ba carbonates) to the table (as stated in the table legend) and add a row with the important comparative CO2 to C +O2 carbon deposition potential, When discussing the latter refer to the calculation that the CO2 to C + O2 deposition potential is not significantly temperature dependent (reference: Ren, J.; Lau, J.; Lefler, M.; Licht, S.; The minimum electrolytic energy needed to convert carbon dioxide by electrolysis in carbonate melts. J. Phys. Chem C 2015, 119, 23342-23349.). It is unlikely that the calculated potentials are correct/precise to three digits after the decimal point, and at best 2 digits should be reported in the table.
   7b) Add the following references a, b, c and l with the pertinent measured potentials in lithium carbonate,a,d,l , and the observed higher potential in both mixed sodium/lithiumd and mixed magnesium, calcium or barium /lithiumb or, mixed sodium/bariumd carbonate electrolytes, and discuss why the observed measured carbon potential in Li2CO3 is much nearer =1 V (more than 0.6 V less negative) than the calculated potential.
   iii) Add two others column for (i) the listed carbonate melting point (723·°C for Li2CO3, etc), and (ii) the calculated potential not only at 500°C, but also at the carbonate melting or decomposition temperature.
   l. Licht, S.; Wu, H. STEP Iron, a Chemistry of Iron Formation without CO2 Emission: Molten Carbonate Solubility and Electrochemistry of Iron Ore Impurities. J. Phys. Chem C 2011, 115, 25138-25147.

Reply: We are thankful to the reviewer for having raised this question. We have corrected the previous calculations, performed the new requested ones and have comprehensively revised

the table and the accompanying discussion in the Supplementary Information. Specifically, we have made the following changes: 7a) We have added rows for the calculated deposition potentials of the alkaline earth metal carbonates, as well as a row for the direct $CO_2 = C + O_2$ reaction. As suggested, we have reduced the precision of all calculated potentials to two decimal places. 7b): References added alongside discussion of the difference between calculated potentials and potentials observed by Licht et al.

For carbon to deposit via reaction mechanism 1 (Eq. S1), the metal deposition potential in the melt must be more positive than the deposition potential for carbon (otherwise, carbon will deposit directly before metal deposition happens via Eq. S2, making reaction mechanism 1 unlikely)[1].

The thermodynamic carbon deposition potentials are thus defined as the potentials for the reaction:

$$3M_2CO_3 = C + 3M_2O + 2CO_2 + O_2 \quad \text{(S1);}$$

and the deposition potentials of metals as the potentials for the reaction:

$$4M_2CO_3 = 4M + 2CO_2 + O_2 \quad \text{(S2).}$$

For alkali earth metals, the reactions are defined as follows:

$$2MCO_3 = C + 2MO + CO_2 + O_2 \quad \text{(S3);}$$

$$2MCO_3 = 2M + 2CO_2 + O_2 \quad \text{(S4).}$$

Supplementary Table 1. Deposition potentials of alkali and alkali earth metals compared to the deposition potential of carbon in pure liquid carbonate electrolytes at 500 °C and at the melting points of the pure carbonates calculated using the HSC Chemistry software as well as the direct carbon electroreduction. All the potentials are reported vs the $CO_3^{2-}/CO_2–O_2$ redox couple.

**Carbonate salt**	**Metal deposition potential at 500** °C	**Carbon deposition potential at 500** °C	**Melting point (°C)**	**Carbon deposition potential at melting point/decomposition temperature**
**Alkali metal carbonates**				
Li_2CO_3	−3.11 V	−1.83 V	723	−1.59 V
K_2CO_3	−2.61 V	−3.19 V	891	−2.74 V
Na_2CO_3	−2.69 V	−2.65 V	851	−2.30 V

**Alkali earth metal carbonates**				
$BeCO_3$	−2.30 V	−0.55 V	54	−0.95 V
$MgCO_3$	−2.52 V	−0.85 V	Decomposes < M.P. (350 ºC)	−0.98 V
$CaCO_3$	−3.16 V	−1.31 V	Decomposes < M.P. (850 ºC)	−1.05 V
$SrCO_3$	−3.23 V	−1.59 V	Decomposes < M.P. (1100 ºC)	−0.70 V
$BaCO_3$	−3.20 V	−1.75 V	Decomposes < M.P. (1740 ºC)	−0.94 V
**Direct carbon electroreduction ($CO_2 = C + O_2$)**				
CO_2	-	−1.02 V	-	−1.03 V (723 ºC)

An alternative thermodynamic explanation has been provided by Licht et al.[2,3] to explain the apparent deposition of carbon at less negative potentials then expected from eq. S1 in pure lithium carbonate melts:

$$Li_2O + 2CO_2 = C + Li_2CO_3 + O_2 \qquad (S5),$$

which was proven to be more consistent with their observed deposition potentials <1 V at increased contents of lithium oxide in the melt.

7. *Regarding Figure S1, assuming that figure presented plots calculated, and not experimental, potential values explain how the carbon deposition potential CO32-/C is determined. For example, is it the lowest of the three values given in Table 1? Why it is so more negative than experimentally observed in pure Li2CO3?*

Reply: We thank the reviewer for requesting this clarification. The carbon deposition potential is indeed defined here from the lowest value in a Li-Na-K eutectic from Supplementary Table

1 and is different from that of pure Li_2CO_3, which is a special case. The apparent discrepancy is described in the answer to the previous question.

8. *Please remove the noise (unrelated distracting hum) from the video.*

Reply: We apologize the noise. It is true that this audio does not give any meaningful information about the reaction. The audio has been removed from the video.

Response to the Referees' comments on the Manuscript:

NCOMMS-25-43100-A

"*Operando* spectroelectrochemical identification of peroxide intermediate in molten carbonate CO_2-to-carbon electroreduction"

Dear Reviewers,

We thank you for your time and for providing insightful comments that have helped us to significantly improve the manuscript. Below, we provide a point-by-point response to the concerns raised. We have addressed all comments (in blue) and accordingly revised the manuscript (in red).

We believe the revised manuscript is now stronger and more suitable for publication in *Nature Communications*.

Reviewer #1:

The authors have given a reasonable explanation to my previous concerns and made satisfactory revision in the revised manuscript. I would recommend its publication now.

Reply: We would like to thank Reviewer #1 for their positive comments and recommendation for publication.

Reviewer #2:

The major revision to address several key points did not occur. It is recommended that the manuscript is published after revision to more fully address the following comments:

1. *The main body of the text states that "most of authors agree that reaction (7) is the most probable reaction for cathodic carbon deposition in molten carbonates: CO32_ + 4e- -> C + 3O2-. Yet the reaction the authors have used to calculate the potentials of alkali carbonate reaction, reaction (S2) is a two-carbonate reaction: 2CO32_ -> C+ 3MO2 +2CO2 +O2. This latter reaction does not explain the low observed potentials for carbon formation. The single carbonate calculated potentials are substantially lower and are more realistic potential values for each of the alkali carbonates. For example, the simple lithium carbonate 500°C reaction potential is approximately is 0.6V less than the 1.83 V (negative sign implied) that the authors' calculate. Occam's Razor would suggest*

that the simpler single-carbonate, rather than two-carbonate reaction, is a better predictor of the system reaction potential. These single carbonate reaction potentials, for the carbonate, alkali reaction M2CO3 -> C +M2O + O2), have already been calculated as a function of temperature, and it is suggested that the single carbonate reaction potentials are used in the Supplementary Table 1 column 2 and 5 values, along with their appropriate references:
1a. Licht, S.; B. Wang; S. Ghosh; H. Ayub; D. Jiang; Ganley, J. New Solar Carbon Capture Process: STEP Carbon Capture. J. Phys. Chem. Lett. 2010, 1, 2363-2368.
1b. Ren, J.; Lau, J.; Lefler, M.; Licht, S.; The minimum electrolytic energy needed to convert carbon dioxide by electrolysis in carbonate melts. J. Phys. Chem C 2015, 119, 23342-23349
Mention of the alkali earth metal carbonate section of the Supplementary Table 1, should include reference to the most recent study in beryllium carbonate, as well as the observed higher (than lithium carbonate measured potentials) potentials in magnesium and barium carbonates:
1c. Licht, G.; Hofstetter, K.; Licht, S.; Beryllium carbonate: a model compound for highest capacity carbon sequestration chemistry, RSC Advances, 14, 40133-40140 (2024); DOI: 10.1039/d4ra07753a
1d. Licht, G.; Hofstetter, K.; Xirui Wang, X.; Licht, S.; A new electrolyte for molten carbonate decarbonization," Communications Chemistry (Nature), 7, 211 (2024); https://doi.org/10.1038/s42004-024-01306-z

Reply: We thank Reviewer #2 for this comment. We have added Supplementary Table 2 to include the single-carbonate reactions, edited the discussion around Supplementary Table 1 and 2 and added references to include the possibility of single-carbonate deposition and non-lithiated systems (as suggested below):

For carbon to deposit via reaction mechanism 1 (Eq. S1), the metal deposition potential in the melt must be more positive than the deposition potential for carbon (otherwise, carbon will deposit directly before metal deposition happens via Eq. S2, making reaction 1 unlikely)[1].
The thermodynamic carbon deposition potentials are thus defined as the potentials for the reaction:

$$3M_2CO_3 = C + 3M_2O + 2CO_2 + O_2 \quad \text{(S1);}$$

and the deposition potentials of metals as the potentials for the reaction:

$$4M_2CO_3 = 4M + 2CO_2 + O_2 \quad \text{(S2).}$$

For alkali earth metals, the reactions are defined as follows:

$$2MCO_3 = C + 2MO + CO_2 + O_2 \qquad (S3);$$

$$2MCO_3 = 2M + 2CO_2 + O_2 \qquad (S4).$$

Supplementary Table 1. Deposition potentials of alkali and alkali earth metals compared to the deposition potential of carbon in pure liquid carbonate electrolytes at 500 °C (according to reactions S1-S4), the melting points of the pure carbonates as well as the potential of direct carbon electroreduction calculated using the HSC Chemistry software. All the potentials are reported vs the $CO_3^{2-}/CO_2–O_2$ redox couple.

**Carbonate salt**	**Metal deposition potential at 500 °C**	**Carbon deposition potential at 500 °C**	**Melting point (°C)**	**Carbon deposition potential at melting point/decomposition temperature**
**Alkali metal carbonates**				
Li_2CO_3	−3.11 V	−1.83 V	723	−1.59 V
K_2CO_3	−2.61 V	−3.19 V	891	−2.74 V
Na_2CO_3	−2.69 V	−2.65 V	851	−2.30 V
**Alkali earth metal carbonates**				
$BeCO_3$	−2.30 V	−0.55 V	54	−0.95 V
$MgCO_3$	−2.52 V	−0.85 V	Decomposes < M.P. (350 °C)	−0.98 V
$CaCO_3$	−3.16 V	−1.31 V	Decomposes < M.P. (850 °C)	−1.05 V
$SrCO_3$	−3.23 V	−1.59 V	Decomposes < M.P. (1100 °C)	−0.70 V
$BaCO_3$	−3.20 V	−1.75 V	Decomposes < M.P. (1740 °C)	−0.94 V
**Direct carbon electroreduction ($CO_2 = C + O_2$)**				
CO_2	-	−1.02 V	-	−1.03 V (723 °C)

The second requirement is that the oxide being formed in the carbon deposition reaction needs to be soluble (for example, successful deposition has been reported in Na-Ba melts, where Ba facilitates the deposition)[2,3]. An alternative reaction has been provided by Licht et al.[4] to explain the apparent deposition of carbon at less negative potentials than expected from eq. S1 in pure lithium carbonate melts:

$$Li_2O + 2CO_2 = C + Li_2CO_3 + O_2 \quad \text{(S5)},$$

which was proven to be more consistent with their observed deposition potentials <1 V at increased contents of lithium oxide in the melt.

The third option for carbon deposition is a direct single-carbonate reduction reaction:

$$M_2CO_3 = C + M_2O + O_2 \quad \text{(S6)},$$

Supplementary Table 2. Deposition potentials of carbon in pure liquid carbonate electrolytes at 500 °C (according to reaction S6) calculated using the HSC Chemistry software. All the potentials are reported vs the $CO_3^{2-}/CO_2–O_2$ redox couple.

**Carbonate salt**	**Metal deposition potential at 500 °C**	**Carbon deposition potential at 500 °C**
**Alkali metal carbonates**		
Li_2CO_3	−3.11 V	−1.29 V
K_2CO_3	−2.61 V	−1.75 V
Na_2CO_3	−2.69 V	−1.57 V

1. *The statement in the manuscript that "no carbon deposits have been witnessed in ... pure carbonates with no lithium is misleading. This should be clarified with reference made to note the study in which extensive carbon deposits have been observed in sodium-barium carbonates, which contain no lithium, and it should be discussed and referenced:*
2a. Liu, X.; Licht, G.; Wang, W.; Licht, S.; CO2 Utilization by Electrolytic Splitting to Carbon Nanotubes in Non-lithiated, Cost-Effective, Molten carbonate Electrolytes" Advanced Sustainable Systems, 2022, 2100481 (2022).

On a related note, the lower potential for sodium carbonate reduction (in the 2V domain) should be substituted in Supplementary Table 1 for the single carbonate reaction as previously discussed. This is lower than the metal deposition potential, and helps understand how the carbon deposition, rather than the competing metal deposition, occurs.

Reply: We thank Reviewer #2 for pointing this out and apologize for not including the Na-Ba system, which has indeed shown carbon deposits as the reviewer has pointed out. However, this discussion was in the context of this manuscript, which deals with Li, Na, and K carbonates. This is why the original sentence reads "**However, no carbon deposits have been witnessed in pure Na-K carbonates with no lithium[21]**." We did not study alkaline-earth-containing mixtures like Na-Ba, which are chemically distinct and outside the scope of this Article. However, it is important to point out that other metals forming soluble oxides are capable of replacing lithium, as the reviewer has pointed out. To make this clear to the reader, we have edited the discussion around Supplementary Tables 1 and 2 as described above.

2. *The discussion in the text relates to equation 8 "Additionally, it has been suggested that CO2 can also be chemically dissolved in the carbonate to form dicarbonate anions (C2O52_), which could then also take part in the reaction mechanism as an intermediate for CO2 capture: CO2 + CO32_ ⇋ C2O52_." This pathway was described in the related reference 34 as having no experimental evidence, and involves a dicarbonate product which has not been observed. It should be replaced, or at least augmented with the much more likely (again Occam's razor…) simple exogenic reaction of CO2 and oxide to make carbonate CO2 + O2_ ⇋ CO32_, which has been thermodynamically evaluated and experimentally discussed as referenced:*
3a. Lau, J.; Dey, G.; Licht, S.; Thermodynamic assessment of CO2 to carbon nanofiber transformation for carbon sequestration in a combined cycle gas or a coal power plant," Energy Conservation and Management, 122, 400-410 (2016).
3b. Wang, X.; Liu, x.; Licht, G.; Wang, B.; Stuart Licht, S.: Exploration of alkali cation variation on the synthesis of carbon nanotubes by electrolysis of CO2 in molten carbonates, Journal of CO2 Utilization, 34, 303-312 (2019).
3c. Ren, J.; Licht, S.; Tracking airborne CO2 mitigation and low cost transformation into valuable carbon nanotubes," Scientific Reports, 6, 27760-1-11 (2016). Nuances of

this CO2 capture reaction have recently been observed, and relevant reactions have been discussed in the previous relevant article (and its Supplementary Materials in the section: "Oxide versus carbonate mechanism of gas evolution in separated half-cell molten carbonate electrolyses."):, which should be mentioned and referenced 3d. Licht, S.; Licht, G.; Hofstetter, K.; Large-scale electrolytic molten carbonate carbon capture and transformation to carbon nanotubes and other graphene nanocarbons" Cambridge Prisms: Carbon Technologies, 1, e6, 1-11 (2025; [https://doi.org/10.1017/cat.2025.10007.](https://doi.org/10.1017/cat.2025.10007)

Reply: Thank you for pointing this out. Our intention here was to be comprehensive in covering proposed reaction mechanisms, in discussion of which this species has been mentioned numerous times, especially in earlier literature. We agree with the reviewer that in high oxide activity melts (such as the direct air capture system here) the simpler reaction of CO_2 capture by the oxide anion is prevalent, and have made this clearer in the manuscript by adding the following sentence: In conditions of high oxide activity (and thus low pCO_2), however, the primary mechanism for CO_2 uptake is the direct, exothermic reaction with oxide ions. This simpler pathway is thermodynamically favored and serves as the fundamental regeneration step for the carbonate electrolyte in low pCO_2 environments such as air[35].

3. *It is suggested that to modify the sentence in the manuscript as shown below below, with the word "allotropes" to reflect the broader study carbon nanotube allotrope study, rather than just the carbon nanotube study, and replacing the relevant reference 13:*
   "By CO2MSE the synthesis of varied nanocarbon products, including various types of carbon nanotube allotropes13, ..."
   replacement for previous reference:
   13. Liu, X., Licht, G., Wang, X. & Licht, S. Controlled Growth of Unusual Nanocarbon Allotropes by Molten Electrolysis of CO2. Catalysts 2022, 12, 125 (2022).

Reply: Thank you for suggesting this. It does indeed make sense to cover other allotropes than CNTs to widen the perspective for the reader. We have added the reference and edited the discussion to include a wider range of allotropes: By CO2MSE the synthesis of varied nanocarbon products, including various types of carbon nanotubes[13], nano-platelets and

graphene[14], doped carbons[15,16], other allotropes[17] and even oxide-carbon composites[18] has been demonstrated.

4. *The observed generation of a peroxide species during carbonate electrolysis at the cathode is a fascinating observation and should be published. However, the conclusion that it is a reactive intermediate participating in a two-electron reaction towards the formation of solid carbon remains a hypothesis. It is one of several alternative possible explanations. This question is compelling in light of the high potential noted in the manuscript for the reactive formation of peroxide from oxide (equation 12). It should discussed and reiterated in the conclusion that this is a hypothesis which needs further experimental validation, for example by suggestion further studies to examine the addition of a quenching reaction for the peroxide to check whether carbon formation than stops; by measuring the number of electrons transferred in the rate limiting step; by probing whether the peroxide species is also formed in the important higher temperature domain in which high purity graphene nano-allotropes, rather than amorphous carbon products, occurs.; or with different metal alloys, or different current densities or carbonate electrolyte compositions, some of which shave been shown to selectively efficiently produce specific carbon allotropes (and may alter local chemistry conditions), and which may be a challenge to discern through spectroscopic measurements.*

Reply: We thank the Reviewer for this discussion, suggestions and finding an important mistake: the high negative potential that we note is necessary for reaction 11 (the first step of forming adsorbed peroxide), not 12, of course. We apologize for this misnumbering. As the discussion and Figure 5 after that explains, this high potential can be lowered by interaction between the peroxide and carbon substrate, making it feasible. While electrochemical kinetic studies in molten salt systems remain challenging due to the high stability required from any experimental system, we agree that inviting further studies to confirm the peroxide pathway proposed here, including more realistic quenching experiments, is an excellent idea and have added the following discussion to the conclusions: We emphasize that further proof, including kinetic studies determining the electron transfer number during the rate-limiting step and chemical quenching experiments of peroxide are necessary to definitively confirm this mechanism.

Systematic studies exploring different CO_2 partial pressures, temperatures, electrodes and electrolyte compositions than used here were already suggested in the last sentence of the Discussion section, but we have added electrode materials and current density as variables, as the Reviewer has suggested and it now reads: This opens new dimensions into understanding the carbon reduction process from direct air capture and industrial waste streams, and enables design of carbon materials from CO_2 with control of structure and real-time process monitoring, but systematic studies at other CO_2 partial pressures, current densities, temperatures, electrodes and electrolyte compositions are essential to widen these prospects.

Response to the Reviewers' comments on the Manuscript:

NCOMMS-25-43100-C

"*Operando* spectroelectrochemical identification of peroxide intermediate in molten carbonate CO_2-to-carbon electroreduction"

Dear Reviewers,

We thank you for your time and for providing insightful comments that have helped us to significantly improve the manuscript. Below, we provide a point-by-point response to the concern raised. We have addressed all comments (in blue) and accordingly revised the manuscript (in red).

We believe the revised manuscript is now stronger and more suitable for publication in *Nature Communications*.

Reviewer #2:

The corrections are nearly complete. After the following suggestion (an addition to GTable S2) is considered, the manuscript is acceptable. After consideration of this correction the manuscript does not require further review by this Reviewer:

The new Supplementary Table 1 puts undo emphasis on the less likely carbon producing reactions S3 and S4 by presenting their calculated potentials at both 500°C and the melting point. This should be balanced in Supplementary Table 2 by adding Table 1's equivalent final column with header "Carbon deposition potential at melting point/decomposition temperature" to Table @, along with potential values appropriate for carbon produced by the S6 reaction. For example the L2CO3 value will be 1.08V. The other values (for K2CO3 and Li2CO3) can be obtained by reference S. Licht et al, "A new Solar Carbon Capture Process: Step Carbon Capture," J. Phys. Chem. Lett. 1, 2363-2368 (2010).

Reply: We thank Reviewer #2 for this comment. Supplementary Table 2 has been edited to include the data suggested:

Supplementary Table 2. Deposition potentials of carbon in pure liquid carbonate electrolytes at 500 °C (according to reaction S6) calculated using the HSC Chemistry software. All the potentials are reported vs the $CO_3^{2-}/CO_2–O_2$ redox couple.

**Carbonate salt**	**Metal deposition potential at 500 °C**	**Carbon deposition potential at 500 °C**	**Carbon deposition potential at melting point/decomposition temperature**
**Alkali metal carbonates**			
Li_2CO_3	−3.11 V	−1.29 V	−1.21 V
K_2CO_3	−2.61 V	−1.75 V	−1.59 V
Na_2CO_3	−2.69 V	−1.57 V	−1.45 V
